# Genetic stock identification of Atlantic salmon (*Salmo salar*) populations in the southern part of the European range

**DOI:** 10.1186/1471-2156-11-31

**Published:** 2010-04-29

**Authors:** Andrew M Griffiths, Gonzalo Machado-Schiaffino, Eileen Dillane, Jamie Coughlan, Jose L Horreo, Andrew E Bowkett, Peter Minting, Simon Toms, Willie Roche, Paddy Gargan, Philip McGinnity, Tom Cross, Dylan Bright, Eva Garcia-Vazquez, Jamie R Stevens

**Affiliations:** 1 Hatherly Laboratories, School of Biosciences, University of Exeter, Prince of Wales Road, Exeter EX4 4PS, UK; 2 Departamento Biologia Funcional, Area de Genética, Universidad de Oviedo, C/Julian Claveria s/n, 33006 Oviedo, Spain; 3 Department of Zoology, Ecology and Plant Science/Aquaculture and Fisheries Development Centre, University College Cork, Cork, Ireland; 4 Ayrshire Rivers Trust, Donald Hendrie Building, Auchincruive Estate, Ayr KA6 5HW, UK; 5 Environment Agency, Cornwall Area Office, Sir John Moore House, Victoria Square, Bodmin PL31 1EB, UK; 6 Central Fisheries Board, Swords Business Campus, Balheary Road, Swords, Co. Dublin, Ireland; 7 Westcountry Rivers Trust, Rain-Charm House, Kyl Cober Parc, Stoke Climsland, Callington, Cornwall PL17 8PH, UK; 8 Marine Biological Association of the UK, The Laboratory, Citadel Hill, Plymouth PL1 2PB, UK; 9 Institute of Zoology, Zoological Society of London, Regent's Park, London NW1 4RY, UK

## Abstract

**Background:**

Anadromous migratory fish species such as Atlantic salmon (*Salmo salar*) have significant economic, cultural and ecological importance, but present a complex case for management and conservation due to the range of their migration. Atlantic salmon exist in rivers across the North Atlantic, returning to their river of birth with a high degree of accuracy; however, despite continuing efforts and improvements in in-river conservation, they are in steep decline across their range. Salmon from rivers across Europe migrate along similar routes, where they have, historically, been subject to commercial netting. This mixed stock exploitation has the potential to devastate weak and declining populations where they are exploited indiscriminately. Despite various tagging and marking studies, the effect of marine exploitation and the marine element of the salmon lifecycle in general, remain the "black-box" of salmon management. In a number of Pacific salmonid species and in several regions within the range of the Atlantic salmon, genetic stock identification and mixed stock analysis have been used successfully to quantify exploitation rates and identify the natal origins of fish outside their home waters - to date this has not been attempted for Atlantic salmon in the south of their European range.

**Results:**

To facilitate mixed stock analysis (MSA) of Atlantic salmon, we have produced a baseline of genetic data for salmon populations originating from the largest rivers from Spain to northern Scotland, a region in which declines have been particularly marked. Using 12 microsatellites, 3,730 individual fish from 57 river catchments have been genotyped. Detailed patterns of population genetic diversity of Atlantic salmon at a sub-continent-wide level have been evaluated, demonstrating the existence of regional genetic signatures. Critically, these appear to be independent of more commonly recognised terrestrial biogeographical and political boundaries, allowing reporting regions to be defined. The implications of these results on the accuracy of MSA are evaluated and indicate that the success of MSA is not uniform across the range studied; our findings indicate large differences in the relative accuracy of stock composition estimates and MSA apportioning across the geographical range of the study, with a much higher degree of accuracy achieved when assigning and apportioning to populations in the south of the area studied. This result probably reflects the more genetically distinct nature of populations in the database from Spain, northwest France and southern England. Genetic stock identification has been undertaken and validation of the baseline microsatellite dataset with rod-and-line and estuary net fisheries of known origin has produced realistic estimates of stock composition at a regional scale.

**Conclusions:**

This southern European database and supporting phylogeographic and mixed-stock analyses of net samples provide a unique tool for Atlantic salmon research and management, in both their natal rivers and the marine environment. However, the success of MSA is not uniform across the area studied, with large differences in the relative accuracy of stock composition estimates and MSA apportioning, with a much higher degree of accuracy achieved when assigning and apportioning to populations in the south of the region. More broadly, this study provides a basis for long-term salmon management across the region and confirms the value of this genetic approach for fisheries management of anadromous species.

## Background

The ability to differentiate between stocks in fisheries is of vital significance for management and conservation of a fishery [1]. It is important to understand how mortality is divided between different components of a fishery, i.e. differential exploitation, so that fisheries can be effectively managed. Without this ability it is difficult to predict the outcomes of conservation plans, to implement effective stock rebuilding programmes, and it is impossible to quantify the contribution and exploitation of each stock to a fishery, such that ultimately they cannot be sustainably managed. Indiscriminate fishing may also lead to the over-exploitation of weak or less productive stocks, threatening their continued existence [2,3].

The case of the Atlantic salmon (*Salmo salar*) is a prime example of the difficulties that managers face in trying to identify differentially exploited stocks in fisheries, as the species is widespread and exploitation can occur sequentially, at a variety of scales (from anglers within catchments, to estuarine or coastal netting and high-seas fisheries). This is a result of the complicated lifecycle of Atlantic salmon, whereby individuals leave their natal rivers, which are spread along the coasts of western Europe and the Baltic sea, and eastern North America, to migrate to feeding grounds off the west coast of Greenland and the Faeroe Islands [4]. They home very accurately to their natal rivers to spawn and the high fidelity of returning salmon provides a behavioural stock isolating mechanism, potentially allowing groups of fish to be reproductively isolated over relatively short geographic distances [5]. The isolated nature of river catchments (and in some cases tributaries) and the sometimes disjunctive nature of suitable spawning/nursery habitat within them, provide an additional, physical mechanism for stock isolation. This appears to have generated widespread genetic differentiation within and between river systems [6-10].

For the last 200 years the Atlantic salmon has been in decline across its native range (e.g. [11]). This can be tied to environmental changes occurring within rivers resulting from a variety of human activities, primarily the exploitation of land and water resources without due care for the health of aquatic ecosystems [4]. In particular, since the 1960s that there has been a steep fall in the numbers of salmon returning to natal rivers to spawn [12] and the commercial exploitation of the species, not only in oceanic fisheries but also in coastal waters, is now viewed as a factor of international importance in terms of its effect on the numbers of fish in spawning runs [4]. Concern over declines have meant most fisheries are now closed or strictly managed by, for example, restrictions on rod catches and buy-outs or closures of near-shore commercial fisheries, but most notably curtailing the high-seas fishery off west Greenland and the Faroe Islands. However, despite fisheries regulation, in general salmon numbers have further declined over this period suggesting that more than over-fishing is responsible for continuing declines [11]. So, it appears the reasons for salmon declines are multi-factorial, and the actual relationship between over-exploitation and other factors requires clarification [2,3]. Therefore, the quantification of mortality due to exploitation has important implications in working out the reasons behind salmon declines, as well as for current management and conservation.

Historically, tagging has generally been employed to identify the specific population of origin for Atlantic salmon [12]. Whilst this method provides one hundred percent accuracy for the very small proportion (e.g. [13]) of marked individuals that are successfully recaptured, no data are available for unmarked individuals. Genetic markers (or tags) are innate, so have the advantage that all fish from a population are inherently marked. Allozyme markers have been successfully employed in studies of Pacific salmonids for decades [14-17] and have provided information on conserving weak stocks, allocating catches among users and elucidating patterns of migratory behaviour in a variety of species [18]. However, levels of variation at allozyme markers in Atlantic salmon have generally been viewed as too low for their successful application in fine-scale/high resolution programmes of genetic stock identification (GSI) [7,19,20].

The development of large numbers of highly polymorphic markers (in particular, variable number repeats) has opened the way for applications of GSI (e.g. [21]) at varying scales: catchment level [22], within country [23], within a region, e.g. the Baltic sea [24,25], and at the continental scale [26-29]. However, the present study represents the first attempt to undertake a detailed and stratified programme of sample collection and genotype analysis of Atlantic salmon from across the entire southern part of the European range of the species. With this aim, samples were collected from rivers in northern Spain, France, England, Wales, Scotland and Ireland, together with a number of estuary net samples, to build a genetic baseline (the ASAP database) for salmon within this region. The success of previous similar studies [22-29] confirms the utility of a microsatellite/GSI-based approach. Accordingly, it is anticipated that this information will provide a robust baseline with which to explore the effects of commercial exploitation of salmon within the region, an area which includes populations at the southern limit of the species range (Spain and southwest France), including those that have undergone some of the steepest recorded declines [11] and those facing the greatest threat from global warming, and possible extinction [30,31]. Not only do salmon in this area face many potentially serious threats to their continued persistence, they have until recently been exploited by a number of mixed stock fisheries, including the Irish drift net fishery (the last major offshore salmon fishery in the North Atlantic, which was suspended in November 2006), which have indiscriminately intercepted adults returning to their natal rivers within the study region, including those from weak and declining populations. However, very few data exist with which to quantify this exploitation [32].

Accordingly, this study presents a genetic baseline for salmon (*Salmo salar*) in the southern part of the eastern Atlantic region, which can be used to identify the origins of salmon sampled from the marine environment. The baseline comprises genetic profiles from 117 putative populations of predominantly juvenile (pre-migratory) salmon sampled from 57 rivers across the region, typed using a suite of 12 microsatellite loci. To validate this genetic baseline, reporting regions were defined; samples of returning adult fish collected from estuary nets or by in-river rod-and-line fishermen were characterised and compared with the baseline using simulations, mixed stock analysis (MSA) and individual assignment analysis. Finally, the importance of these reporting regions and their associated regional genetic signatures, which appear to be independent of more commonly recognised terrestrial biogeographical and political boundaries, and the implications of these findings on the accuracy of MSA are evaluated.

## Methods

### Baseline Sample Collection

**Figure 1:**
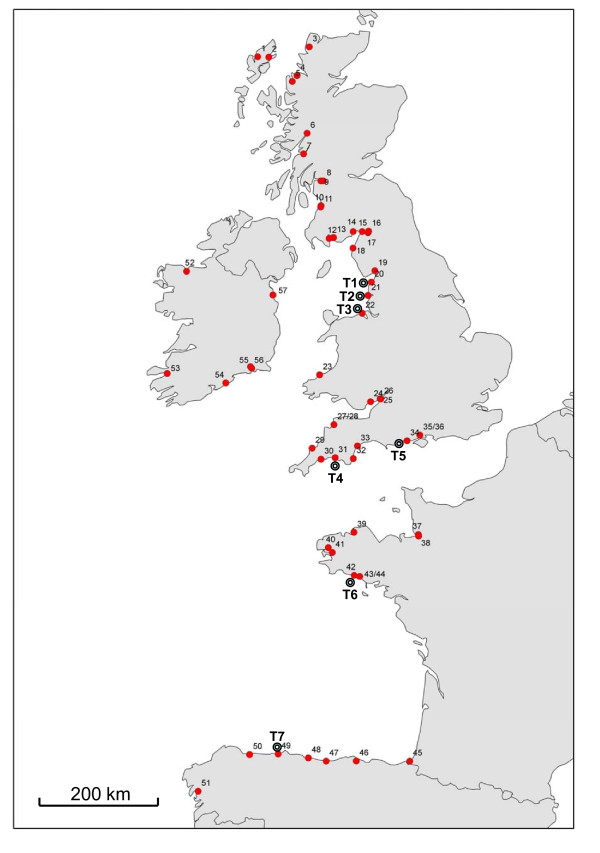
**Map of the river mouth locations for rivers included in the project**. Key to catchments analysed: (1 - 15, Scotland) 1: Blackwater; 2: Creed; 3: Laxford; 4: Gruinard; 5: Ewe; 6: Loch Lochy; 7: Awe; 8: Loch Lomond; 9: Clyde; 10: Ayr; 11: Doon; 12: Cree; 13: Fleet; 14: Nith; 15: Annan; (16 - 36, England and Wales) 16: Esk; 17: Eden; 18: Derwent; 19: Kent; 20: Lune; 21: Ribble; 22: Dee; 23: Teifi; 24: Usk; 25: Wye; 26: Severn; 27: Taw; 28: Torridge; 29: Camal; 30: Fowey; 31: Tamar; 32: Dart; 33: Exe; 34: Avon; 35: Itchen; 36: Test; (37 - 45, France) 37: Sée; 38: Sélune; 39: Léguer; 40: Élorn; 41: Aulne; 42: Ellé; 43: Scorff; 44: Blavet; 45: Nivelle; (46 - 49, Spain) 46: Asón; 47: Cares; 48: Sella; 49: Narcea; 50: Eo; 51: Ulla; (52 - 57, Ireland) 52: Moy; 53: Laune; 54: Cork Blackwater; 55: Barrow; 56: Suir; 57: Boyne. Full details of catchments studied are given in Additional File 1.

Specimens of 3730 Atlantic salmon were collected from 57 rivers across 117 sample sites that drain into the eastern Atlantic Ocean, the English Channel, the Irish Sea and the Bay of Biscay to form the baseline for GSI. Rivers with a combination of the largest catchment area and rod-catch were preferentially targeted; a full list of sample sites included in the survey is given in Additional File 1. A map summarising the rivers included in the study is given in Fig. 1. The majority of sampling was carried out in 2004 and 2005 during routine juvenile salmon abundance surveys and targeted 1+ parr, thus reducing fishing effort and in-river disturbance to salmon. Specimens originating from northwest France are an exception; for these samples, scales from rod-caught adult salmon were utilized. All tissues (fin clips and scales) were collected in the field as part of routine national fisheries monitoring and management programmes; all sampling conformed to national agency ethical guidelines.

### Test Sample Collection

Specimens of returning adult salmon, independent of the baseline, were also analysed in order to assess the accuracy of the GSI analyses. These were collected from commercial estuary nets or rod-and-line fishermen from seven different rivers in Spain, France, England and Wales (for details see Table 1). For all rivers from which returning adult salmon were analysed, baseline juvenile samples were also collected; the one exception to this was the river Aven in northwest France, from which a sample of adult salmon was analysed, but no sample of juvenile fish was collected for inclusion in the baseline.

**Table 1:** Details of test samples.

Test Sample ID	Name, Location	Latitude/Longitude (Grid Ref)	Source	Year	Sample Size
T1	Lune, N.W. England	N 53:59:54/53.9984 W 02:51:02/-02.8504	Estuary nets	2004-05	49

T2	Ribble, N.W. England	N 53:41:00/53.6833 W 02:50:00/-02.8333	Estuary nets	2004	50

T3	Dee, N. Wales	N 53:22:31, 53.3754 W 03:13:30/-03.2175	Estuary nets	1984-88	47

T4	Tamar, S.W. England	N 50:21:30/50.3583 W 04:10:00/-4.1667	Estuary nets	1987	62

T5	Mudeford (close to mouth of the Avon), S. England	N 50:43:21/50.7224 W 01:44:55/-01.7485	Estuary nets	2006	47

T6	Aven, N.W. France (not included in the baseline)	N 47:48:06/47.8017; W 03:44:07/-03.7353	Rod-caught from across the catchment	2005	37

T7	Narcea, N. Spain	N 43:28:00/43.4670 W 06:07:00/-06.1170	Rod-caught from across the catchment	2006	112

Tissue samples from adult salmon were collected from novel sites, independent of the baseline data; see Fig 1. for locations of tests samples.

The high fidelity with which adult Atlantic salmon return to natal rivers would suggest that the majority of adult fish analysed in the test samples are native to the catchments in which they were caught. As such, they provide an independent opportunity (over and above self- assignment) to assess the suitability of the baseline data for applications of genetic stock identification; however, allocation of a proportion of a sample to a neighbouring river (or rivers) may also provide insight into the extent of mixed stock exploitation occurring within these commercial catches, particularly in the case of estuary net fisheries, e.g. [14,33].

### Microsatellite Analysis

Genomic DNA was extracted from scales or fin clips of Atlantic salmon according to a chelex resin protocol [34]. Variation was determined at 12 microsatellite loci (Table 2): Ssa157a [35], SsaD 144b [35], Ssa197 [36], Ssa202 [36], Ssa171 [36], SSsp 2210 [37], SSsp 1605 [37], SSsp 2201 [37], SSsp G7 [37], Ssa289 [38], Ssosl417 [39], Ssosl85 [39]. Genotypes were assayed through polymerase chain reaction (PCR) and polyacrylamide gel electrophoresis with fluorescently labelled primers. PCR amplifications were performed on reaction mixtures containing approximately 50 ng of extracted Atlantic salmon DNA template, 10 nM Tris-HCl pH 8.8, 1.5-2.5 mM MgCl_2_, 50 mM KCl, 0.1% Triton ×-100, 0.35 μM of fluorescently labeled primers, 0.5 Units of DNA Taq Polymerase (PROMEGA, Madison, WI, USA) and 250 μM of each dNTP in a final volume of 20 μL. Full details of PCR conditions for each microsatellite used in this study are reported in a previous publication from this research programme [40].

**Table 2:** Characteristics of the 12 microsatellite loci used in this study.

Locus	Size Range	No. Alleles	H_E_	H_O_	Global F_ST_
SSsp2201	220-376	38	0.9525	0.9357	0.02699

Ssosl85	177-233	29	0.8663	0.8220	0.04346

Ssa171	194-272	31	0.9026	0.8563	0.05203

Ssa157a	246-450	40	0.9454	0.9270	0.02526

SsaD144b	113-273	40	0.9540	0.9324	0.02931

Ssa289	112-132	8	0.6615	0.6342	0.04992

SSsp G7	88-212	32	0.8994	0.8085	0.04315

SSOSL 417	141-209	33	0.9132	0.8506	0.05567

Ssa197	151-275	32	0.9142	0.8142	0.03479

Ssa202	196-320	26	0.8836	0.8402	0.03898

SSsp1605	214-266	13	0.8238	0.7741	0.04883

SSsp 2210	104-172	18	0.8165	0.7590	0.04687

Expected heterozygosity (H_E_) and observed heterozygosity (H_O_).

The microsatellite analysis was carried out in two laboratories; each used an exclusive set of six loci to keep identification of alleles as consistent as possible (Exeter: Ssa157a, SsaD144b, Ssa171, SSsp 2201, Ssa289, Ssosl85; Oviedo: Ssa197, Ssa202, SSsp 2210, SSsp 1605, SSsp G7, Ssosl417), whilst also avoiding the need for cross-laboratory standardisation. The size determination of the labelled PCR products was performed using both a Beckman-Coulter CEQ8000 automatic DNA sequencer with the associated fragment analysis software (Beckman-Coulter) at the University of Exeter and an ABI 3100 with the GENEMAPPER V.3.5 (Applied Biosystems) software at the DNA Sequencing Unit of the University of Oviedo.

### Statistical Treatment

Each baseline sample at each locus was tested for conformity to Hardy-Weinberg equilibrium (HWE) [41], using GENEPOP 3.4 [42]. In tests for departures from HWE, temporal samples were pooled unless significant differences in allele frequencies were detected in multiple loci. Critical levels of significance for simultaneous tests were adjusted using the sequential Bonferroni procedure for multiple tests [43]. Scoring errors, large allele dropout and the presence of null alleles were tested for using the program MICRO-CHECKER [44]. Temporal stability of frequencies (for the rivers with repeated samples: Ayr, Exe, Itchen, Dart, Cares, Sella and Narcea) was also tested with the program GENEPOP (Fisher's exact tests). In subsequent analyses, temporal samples from an individual location were combined to estimate population allele frequencies, as recommended by Waples [45].

Pair-wise and global F_ST _values [46] and estimation of the variance components in allele frequencies among countries (Spain, France, England, Wales, Scotland and Ireland), regions and years [47] were calculated using ARLEQUIN v.3 [48]. In addition, ARLEQUIN was also used to determine the significance of the F_ST _values through permutation tests (10,000 permutations were used). The D_A _distance [49] was used to quantify genetic differentiation between samples. Neighbour-joining phylograms were constructed and confidence estimates of tree topology were calculated by bootstrap re-sampling of loci 1000 times, utilizing the programs Powermarker [50] and Consense (from PHYLIP 3.6 [51]). Genetic distances between samples were also visualized using multi-dimensional scaling (MDS) with Primer 6 [52] and the Bayesian clustering package STRUCTURE v.2.3 [53] was used to identify the most likely number of clusters (*K*) present in the data set, by pursuing solutions that are, as far as possible, in Hardy-Weinberg and linkage equilibrium.

### Estimation of Stock Composition

The statistical package ONCOR [54]), which employs a maximum-likelihood approach, was used to assess the suitability and accuracy of the baseline data for MSA. ONCOR is reportedly [54] less biased and less prone to over-estimation of the predicted accuracy of MSA (particularly with smaller baseline sample sizes) than some previously used software packages, e.g. SPAM [55]. The program determines genotypic frequencies for each locus in each baseline sample and uses the re-sampling method of Andersson et al. [56], which is based on leave-one-out cross validation, to simulate mixture genotypes and to estimate their probability of occurring in the baseline samples. The mean and variance estimates were produced from 100 simulations; the aim of this procedure was to simulate the random variation involved in the collection of baseline and mixture samples.

#### Simulated single-sample mixtures

Simulations of mixtures of fish originating from a single baseline sample (i.e. 100% from one sample) have frequently been used to assess the accuracy of MSA [22,57]. While such an approach may be unrealistic, it provides an initial benchmark for assessment of the accuracy of the estimates of stock composition. It also affords the opportunity to experiment with the exclusion/inclusion of problematic data and the grouping or pooling of baseline samples. Accordingly, simulations were first conducted on data sets comprising 10 and 12 loci; the 10 loci data set excluded two loci (Ssa197 and SSspG7) identified using MICRO-CHECKER [44] as containing a large number of null alleles (see Results section). Comparison of relative apportionment levels between the two data sets allowed the potential benefits (or otherwise) of including loci with a large number of null alleles for MSA to be assessed. Secondly, simulations were run apportioning the simulated mixtures at three levels: to individual sample sites, to river catchments (often incorporating multiple sample sites) and to geographically broader reporting regions (hereafter referred to as reporting regions, which incorporate multiple catchments and reflect inter-relationships between samples). Stock proportions were estimated for each of the individual baseline samples and then summed within groups, catchment or region (the allocate and sum method [58]). In addition, for catchments where multiple sites had been sampled, the allele frequencies were also pooled across sites before running the analysis (the pool and allocate method). While this latter approach is generally applied only when allele frequencies between samples are similar, the approach can also be employed - as in this study - as a potential method of overcoming small baseline and test sample sizes [20,58]. Additionally, to facilitate comparison of our data with a wide range of previous studies, e.g. those focusing on Pacific salmon [18], simulated mixtures were produced for each of the baseline samples with SPAM (version 3.7 [55]; see Additional File 2).

#### Simulated multi-sample mixtures

Two sets of four additional simulations, comprising mixtures of fish originating from a variety of baseline samples, were also evaluated. In the first set, each simulation comprised eight baseline samples, with each sample contributing 12.5% of the overall mixture sample (Table 3). In the second set, each simulation again comprised eight baseline samples, but in these simulations the proportion of each stock was unequal and ranged between 5 - 30% of the overall mixture sample (Additional File 3). Obviously, in a real mixed fishery sample, it is highly unlikely that the proportions of all stocks present would be equal; thus, together, these mixtures provide an opportunity to test the accuracy and precision of the MSA using more complex and diverse fisheries proportions. Estimated stock compositions were determined on the basis of both individual sample sites and at the level of reporting region, using the 'allocate and sum' method [58].

**Table 3:** Multi-sample simulations in ONCOR using equal proportions of baseline samples.

Baseline sample	Proportion	Sample estimate (SD)	Regional estimate (SD)
**Mixture 1**			

CARES (2002)	0.125	0.0831 (0.0197)	0.1199 (0.0097)

CORK BLACKWATER (Clydagh)	0.125	0.0381 (0.0255)	0.0958 (0.0290)

ELORN	0.125	0.0714 (0.0230)	0.1216 (0.0134)

FOWEY (Treverbyn)	0.125	0.0572 (0.0232)	0.1303 (0.0268)

GRUINARD (Ghiubhsachain)	0.125	0.0593 (0.0193)	0.1042 (0.0279)

LOCH LOMOND (Fruin)	0.125	0.1109 (0.0115)	0.1464 (0.0235)

NITH (River Cairn)	0.125	0.0289 (0.0218)	0.1640 (0.0317)

TEST	0.125	0.1130 (0.0085)	0.1179 (0.0040)

**Mixture 2**			

AVON (Bugmoor Hatches)	0.125	0.0761 (0.0186)	0.1183 (0.0037)

CORK BLACKWATER (Awnaskirtaun)	0.125	0.0349 (0.0201)	0.1112 (0.0304)

DOON (Ness Glen)	0.125	0.0601 (0.0191)	0.0981 (0.0244)

EO	0.125	0.1134 (0.0086)	0.1182 (0.0069)

LOCH LOCHY (Lundy Tributary)	0.125	0.0489 (0.0205)	0.1014 (0.0265)

EDEN (Swindale Beck)	0.125	0.0614 (0.0232)	0.1805 (0.0347)

SEE	0.125	0.0775 (0.0192)	0.1126 (0.0134)

WYE	0.125	0.0502 (0.0204)	0.1597 (0.0244)

**Mixture 3**			

CARES (Casano)	0.125	0.1042 (0.0107)	0.2370 (0.0067)
	
NARCEA (2002)	0.125	0.0749 (0.0199)	

SCORFF	0.125	0.0604 (0.0226)	0.2298 (0.0158)
	
SELUNE	0.125	0.0694 (0.0253)	

AVON (Avon Bridge)	0.125	0.0941 (0.0180)	0.2387 (0.0036)
	
ITCHEN	0.125	0.1262 (0.0153)	

DEE (Abbey Brook)	0.125	0.0549 (0.0199)	0.1960 (0.0239)
	
EXE (Fernyball, Sherdon Water)	0.125	0.0516 (0.0166)	

**Mixture 4**			

SUIR (Beakstown)	0.125	0.0175 (0.0170)	0.2247 (0.0419)
	
CORK BLACKWATER (Clydagh)	0.125	0.0457 (0.0250)	

ESK (Boyken Burn)	0.125	0.0315 (0.0238)	0.2713 (0.0394)
	
RIBBLE (Hammerton Hall, River Hodder)	0.125	0.0595 (0.0218)	

BOYNE (Skane Lwr)	0.125	0.0730 (0.0213)	0.2455 (0.0344)
	
AYR (Lugar Water)	0.125	0.1102 (0.0251)	

EWE (Talladale, Grudie Bay)	0.125	0.0447 (0.0211)	0.1977 (0.0381)
	
LAUNE (Cottoners)	0.125	0.0716 (0.0284)	

Four simulated mixtures are presented; each mixture comprised eight samples (each making up 12.5% of a 100% individual mixture) with mixtures 1 and 2 including a river from each reporting region, mixture 3 a combination of southern rivers (incorporating two rivers from each region) and mixture 4 a combination of northern rivers (incorporating two rivers from each region). Stock compositions were estimated at the level of individual sample sites (3rd column, 'Sample Estimate') and reporting regions (4^th ^column, 'Regional Estimate') via the allocate and sum method. One-hundred fish were used in the mixture sample, with 100 simulations. Individual samples are identified by tributary name; see Additional File 1 for details of latitude and longitude, collection date and individual sample size.

Finally, it should be noted that simulations provide an optimistic measure of the accuracy of estimates as they assume that the baseline samples are representative of the populations present in the mixed stock fisheries and, therefore, do not take account of unrepresentative baseline sampling or omitted baseline stocks. Further validation of the baseline data set with samples of known origin is therefore required to fully assess the validity of the above assumption.

#### Test samples

Apportionment of adult samples of known origin was conducted with both ONCOR [54] and cBAYES, of which the latter carries out MSA using a Bayesian algorithm and has been shown in comparison studies to out-perform some maximum likelihood methods [24,57]. In the MSA of test samples with cBAYES, eight 20,000-iteration Monte Carlo Markov chains (MCMC) of estimated stock composition were produced; the starting values for each chain were set at 0.90 for the different samples used to initialize each of the chains. The estimates of stock composition from the test samples were considered to have converged once the shrink factor was less than 1.2 for the eight chains [21]. The last 1,000 iterations from each of the eight 20,000-iteration Monte Carlo Markov Chains were combined and used to obtain the mean and standard deviation of the estimated stock composition.

### Individual Assignment

Both ONCOR and cBAYES were used to test the suitability of the baseline data for applications of assigning individual salmon to rivers or regions of origin. As with the validation of the MSA, the adult salmon samples from the estuary nets or rod-and-line fisheries provided a set of samples of known origin (assuming they originated from the catchment of capture) that were assigned to catchment and region using the summed and pooled baseline datasets. Assignment was limited to those individuals genotyped successfully at nine or more loci and the catchment of origin was determined as that with the highest probability of assignment.

## Results

### Microsatellite Variability

All 12 microsatellites examined were polymorphic in all samples surveyed. The level of heterozygosity was generally very high (Additional File 4, summarized in Table 2), with observed heterozygosity of each locus over all samples as follows: SSsp2201 0.9357 (sample range 0.7500-1.0000), SSOSL85 0.8220 (0.6000-1.0000), Ssa171 0.8563 (0.5870-1.0000), Ssa157a, 0.9270 (0.75000-1.0000), SsaD 144b 0.9324 (0.7333-1.0000), Ssa289 0.6342 (0.4081-0.8787), Ssa197 0.8142 (0.4286-1.0000), Ssa202 0.8402 (0.6176-1.0000), SSsp1605 0.7741 (0.5294-1.0000), SSsp2210 0.7590 (0.4490-1.0000), SSspG7 0.8085 (0.5918-1.0000), Ssosl417 0.8506 (0.6000-1.0000).

In order to assess levels of genotyping error in the dataset, 340 individuals were genotyped for a second time by each laboratory and the proportion of alleles that were scored inconsistently between runs was used to estimate the error rate in the dataset. This revealed an average allelic error rate per locus of 0.022, the lowest rate was 0.014 (associated with locus SSOSL85), and the highest rate was 0.027 (associated with locus Ssa197).

Analysis of microsatellite data with the program MICRO-CHECKER (at the 95% confidence level) highlighted the existence of null alleles at some loci (Additional File 5; a total of 85 significant cases were identified, which compared to an expected value of 65 with Bonferroni corrections applied; α = 0.05). Over half these significant results were associated with loci Ssa197 and SSspG7, strongly suggesting null alleles at these loci. Accordingly, simulations were undertaken both including and excluding these two loci. However, despite the presence of null alleles, their inclusion generally improved overall levels of assignment/apportionment and, as the primary objective of this study was assignment testing and MSA, these loci were included in the final analysis. This result is akin to the findings of Beacham *et al*. [59,60], who demonstrated that inclusion of loci out of HWE provided more accurate results in simulations. A similar issue has been addressed previously by Carlsson [61] who demonstrated that, while the bias in assignment tests caused by null alleles may lead to a slight reduction in assignment power and overestimation of F_ST_, these factors probably do not otherwise alter the overall outcome of assignment testing. Accordingly, affected loci may be included in this type study.

The four samples from the Narcea, Asón, Sella (Spain) and Nivelle (Spain/France) that were collected in 2004 all deviated significantly from conformity to HWE at multiple loci (P < 0.05, corrected across populations) and were excluded from further analysis. Deviation from the expectations of HWE may be due to the steep decline in salmon numbers that is known to have occurred in this region, e.g. [62], and/or may be the result of past stocking and supportive breeding practices. The remaining 16 significant departures from expectations of HWE were spread across different samples and there was no evidence of a consistent departure from HWE at any particular marker, except locus Ssa197 that accounted for half of the significant results (probably due to null alleles; see above). Further testing revealed these deviations were generally the result of a deficiency of heterozygotes (Additional File 5). This may have been the result of 'allelic dropout', i.e. a failure to amplify the larger allele in heterozygote individuals. While the sampling of juvenile fish for the genetic baseline used only 1+ parr (rather than fry), departures from the expectations of HWE may also be due to the effects of non-representative sampling or 'family sampling' [63,64]; in such a case, however, affected populations should be out of HWE at all loci.

Following the removal of four of the 2004 temporal replicates from Spanish/French rivers (see above) temporal samples were available for five sites in the baseline. Testing for temporal changes in allele frequencies at these sites revealed that significant changes had occurred at four loci (P < 0.05, corrected across loci) between the 2005 and 2006 samples from the Postbridge site on the River Dart, southwest England (Additional File 1, DART_(32) samples). However, all other temporal samples showed significant changes at only a single locus, generally not the same locus across different sample sites.

### Use of Genetic Distance Analysis in Defining Reporting Regions

**Figure 2:**
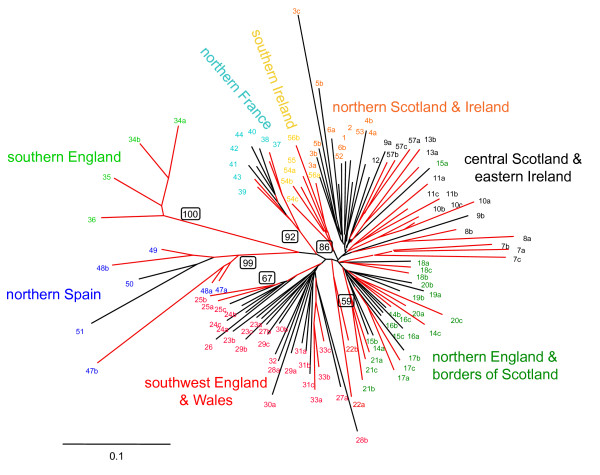
**Neighbour-joining dendrogram of relationships defined between samples of Atlantic salmon analysed in this study**. The D_A _distance measure of Nei *et al*. [49] was used and nodes highlighted in red were supported by bootstrap support of greater than 50% in 1000 pseudoreplicates; bootstrap values for key clades are given in a box on the relevant node. Colours and labels denote reporting region as follows: northern England & borders of Scotland (dark green), southwest England & Wales (red), northern Spain (blue), southern England (light green), northern France (light blue), southern Ireland (yellow), northern Scotland and Ireland (orange), central Scotland and eastern Ireland (black). Sample abbreviations follow those given in Additional File 1.

**Figure 3:**
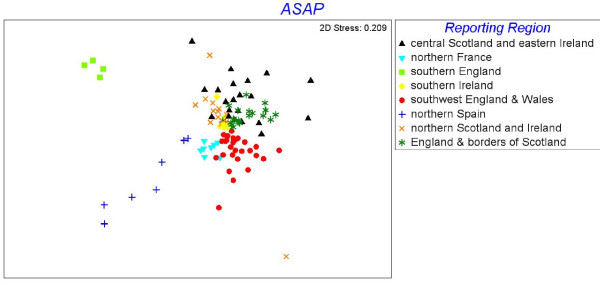
**Multi-dimensional scaling plot of genetic distances between the samples of Atlantic salmon analysed in this study**. The D_A _distance measure of Nei *et al*. [49] was used. Colours denote reporting region and follow Fig. 2: northern England & borders of Scotland (dark green), southwest England & Wales (red), northern Spain (blue), southern England (light green), northern France (light blue), southern Ireland (yellow), northern Scotland and Ireland (orange), central Scotland and eastern Ireland (black).

Pair-wise genetic distances between samples (Fig. 2) show that even between geographically proximate samples genetic distances remained relatively high, but the greatest genetic distances were generally observed between samples in different regions and the lowest genetic distances between samples within catchments or regions. Furthermore, values for bootstrap support generally only exceeded 50% in the most radial nodes/branches in the tree, suggesting some clustering of samples by catchment or neighbouring catchment. There are some notable exceptions to the generally low bootstrap values observed towards the centre of the tree; three distinct clusters of samples from Spain, northern France, and southern England all had bootstrap support greater than 90%. Strong bootstrap support was also observed between samples from the south of Ireland, which also form a distinct group in the tree. Otherwise, while regional structure is evident in the dendrogram (Fig. 2), particularly in the separation of samples collected in northern versus southern areas, the low bootstrap support evident at this broad level makes it difficult to piece together the relationships between salmon in different areas with any certainty. Similarly, the MDS plot (Fig. 3) demonstrates relatively distinct clusters of samples from the southern regions, but failed to distinguish clearly between samples collected across northern England, Scotland and Ireland (a pattern also repeated in the STRUCTURE analysis of the data, Additional file 6). Analysis and presentation of the data using two complementary methods (MDS and phylogeny) allowed reporting regions to be defined; from a practical perspective, use of two different presentation methods allowed the samples included in each group to be readily visualised.

Nevertheless, a lack of strong support for genetic relationships between samples of salmon in the northernmost areas analysed made the definition of reporting regions for the MSA problematic across some parts of the study area. Strong regional population structure is of critical significance in the application of molecular markers to MSA as it allows the assumption to be made that the portion of a fishery derived from un-sampled populations will be allocated to sampled populations in the same area. Despite some variation in the robustness of genetic differentiation between populations from across the study area, we were able to group the baseline samples into eight candidate reporting regions that reflected the shape and relationships supported within the dendrogram (Fig. 2) and MDS plot (Fig. 3), and catch proportions were subsequently calculated for these eight groups. A small number of samples, e.g. Annan and Awe, grouped outside their geographic area or occupied an intermediate position in the tree, and their placement into reporting regions reflects the geographic relationship between samples.

Analysis of F_ST _showed that 4.00% of the observed variation could be accounted for by inter-sample differences (p < 0.00001; locus specific F_ST _values are summarised in Table 2). The pair-wise F_ST _values are summarised in Additional File 7, and demonstrate a range of 0.00 (WYE_Edw - WYE_Garth_Dulas) to 0.15 (ULLA - LAXFORD_Thull). The majority of comparisons are significant at the 0.05% level (5455 out of 5778), and many of the non-significant results occurred between samples collected within the same catchment (38 pair-wise tests). Quantitative estimates of hierarchical gene diversity (Table 4) also revealed that significant genetic differentiation was present at every level tested, with the greatest amount due to within-sample variation. The results showed that 1.09% of genetic variation occurred between temporal samples compared to 4.70% due to variation between sample sites. The differentiation observed between countries (Spain, France, England, Wales, Scotland and Ireland) accounted for 1.42% of the genetic variation, compared to 2.87% among samples within countries, whereas 1.90% of the genetic variation occurred between river catchments, compared with 1.54% due to differentiation among collections within each catchment. Hierarchical analysis of the reporting regions defined for the MSA showed that differentiation between regions accounted for 1.99% of the genetic variation, but 2.30% was also attributed to differentiation within regions.

**Table 4:** Hierarchical genetic diversity analysis.

Source of Variation	Total Variation	Percent of Total	F_CT_	F_SC_	F_ST_
Total	5.202	100			
Between countries	0.074	1.42	0.014		
Among samples within countries	0.149	2.87		0.029	
Within samples	4.978	95.70			0.043

Total	5.201	100			
Between reporting regions	0.103	1.99	0.020		
Among samples within reporting regions	0.120	2.30		0.022	
Within Samples	4.978	95.71			0.043

Total	5.204	100			
Between catchments	0.099	1.90	0.019		
Among samples within catchments	0.080	1.54		0.016	
Within samples	5.025	96.56			0.034

Total	5.225	100			
Between sample sites	0.246	4.70	0.047		
Among temporal samples	0.057	1.09		0.011	
Within samples	4.922	94.21			0.058

The analysis of sources of variation when grouping samples according to country or reporting region utilised the entire data set, encompassing all 108 sample sites. Remaining diversity analyses were restricted to those sampling sites with temporal replicates (10 samples) or those catchments with multiple sites within them (87 samples). All diversity estimates were statistically significant at the p < 0.0001 level, based on probabilities derived from 10,000 permutations.

### Analysis of Simulated Mixtures

#### Analysis of Simulated Single Sample Mixtures

Analysis of simulated mixtures is generally considered the first step in evaluating the effectiveness of a baseline for MSA, affording the opportunity to experiment with assembly of baseline data. Initially, the effect of removing loci SSspG7 and Ssa197 (that were associated with null alleles) from the baseline, was assessed by examining the average apportionment to correct sample with simulated single sample mixtures across all 108 samples (Table 5). The inclusion of these loci led to an average level of apportionment to correct sample of 0.55, compared to a level of 0.50 when they were removed. This result is consistent with previous work suggesting loci with null alleles may still provide useful information in MSA [60,61]; therefore, these loci were incorporated into all subsequent analyses.

**Table 5:** Estimated proportion (with standard deviation) of the simulated single-population mixtures that is apportioned back to the correct baseline sample using ONCOR.

Catchment	Sample Code	Sample Size	a) Sample Allocation (10 loci)	b) Sample Allocation (12 loci)	c) Catchment Allocation	d) Pooled Catchment Allocation	e) Regional Allocation
**ANNAN**	ANN.Bk	30	0.5791 (0.0612)	0.5541 (0.0531)	0.5908 (0.0558)		0.7673 (0.0454)
							
	ANN.Ev	35	0.2918 (0.0558)	0.3317 (0.0635)	0.4073 (0.0593)	0.5682 (0.0706)	0.8931 (0.0303)
							
	ANN.Whi	31	0.2939 (0.0535)	0.3210 (0.0636)	0.4062 (0.0611)		0.7882 (0.0497)

**AULNE**	AULNE	39	0.3475 (0.0597)	0.4081 (0.0621)	0.4081 (0.0621)	0.3932 (0.0645)	0.8717 (0.0347)

**AVON**	AVON.Brd	23	0.7956 (0.0509)	0.8068 (0.0418)	0.9590 (0.0258)	0.9830 (0.0163)	0.9993 (0.0023)
							
	AVON.Bug	20	0.6040 (0.0666)	0.6657 (0.0541)	0.9042 (0.0325)		0.9935 (0.0072)

**AWE**	AWE.Bra	35	0.9551 (0.0210)	0.9673 (0.0192)	0.9703 (0.0189)		0.9751 (0.0152)
							
	AWE.Cla	35	0.9203 (0.0293)	0.9553 (0.0202)	0.9575 (0.0195)	0.9516 (0.0223)	0.9619 (0.0178)
							
	AWE.Mai	35	0.8611 (0.0393)	0.8929 (0.0314)	0.8953 (0.0306)		0.9127 (0.0353)

**AYR**	AYR.Gle	30	0.4368 (0.0639)	0.4197 (0.0609)	0.9756 (0.0164)		0.9786 (0.0124)
							
	AYR.How	30	0.3265 (0.0616)	0.4122 (0.0590)	0.7047 (0.0620)	0.9524 (0.0227)	0.7748 (0.0551)
							
	AYR.Lug	68	0.7924 (0.0523)	0.8418 (0.0490)	0.9173 (0.0327)		0.9381 (0.0248)

**BARROW**	BAR.Bal	40	0.5409 (0.0530)	0.5224 (0.0600)	0.5224 (0.0600)	0.4629 (0.0617)	0.7475 (0.0463)

**BLAVET**	BLAVET	49	0.3544 (0.0640)	0.5474 (0.0601)	0.5474 (0.0601)	0.5415 (0.0649)	0.9445 (0.0245)

**BLACKWATER**	ROAG.Tar	33	0.4980 (0.0580)	0.5202 (0.0616)	0.5202 (0.0616)	0.4621 (0.0547)	0.7290 (0.0486)

**BOYNE**	BOY.Dee	36	0.7222 (0.0575)	0.7764 (0.0492)	0.8785 (0.0389)		0.9070 (0.0294)
							
	BOY.Moy	35	0.6724 (0.0592)	0.7129 (0.0528)	0.8041 (0.0424)	0.9169 (0.0305)	0.8372 (0.0434)
							
	BOY.Ska	35	0.5359 (0.0552)	0.6703 (0.0596)	0.7859 (0.0478)		0.8135 0.0417)

**CAMAL**	CAM.Del	30	0.4496 (0.0693)	0.5280 (0.0611)	0.5735 (0.0610)		0.8119 (0.0496)
							
	CAM.Gam	30	0.5369 (0.0638)	0.6511 (0.0559)	0.6977 (0.0562)	0.7511 (0.0495)	0.8668 (0.0405)
							
	CAM.Ken	30	0.3952 (0.0570)	0.4270 (0.0613)	0.5024 (0.0612)		0.8318 (0.0492)

**CARES**	CARES02	72	0.6363 (0.0615)	0.6855 (0.0547)	0.6866 (0.0548)	0.7858 (0.0525)	0.9831 (0.0120)
							
	CAR.Cas	24	0.9140 (0.0361)	0.9113 (0.0316)	0.9692 (0.0239)		0.9981 (0.0038)

**CLYDE**	CLY.Boc	30	0.4885 (0.0571)	0.5693 (0.0579)	0.5712 (0.0576)	0.7160 (0.0505)	0.6497 (0.0552)
							
	CLY.Cal	25	0.7475 (0.0494)	0.7987 (0.0460)	0.8014 (0.0460)		0.8456 (0.0395)

**CORK BLACKWATER**	COR.Awn	35	0.3076 (0.0596)	0.3599 (0.0577)	0.6915 (0.0516)		0.7493 (0.0441)
		
	COR.Cly	35	0.3581 (0.0629)	0.3624 (0.0599)	0.6443 (0.0521)	0.7640 (0.0468)	0.7193 (0.0585)
							
	COR.Gle	35	0.2661 (0.0574)	0.3763 (0.0596)	0.5453 (0.0553)		0.5836 (0.0591)

**CREE**	CREE.Whi	39	0.4748 (0.0641)	0.5826 (0.0603)	0.5826 (0.0603)	0.5111 (0.0612)	0.6371 (0.0589)

**CREED**	CREED.All	36	0.6212 (0.0545)	0.6257 (0.0551)	0.6257 (0.0551)	0.5744 (0.0575)	0.7530 (0.0592)

**DART**	DAR.Pos	84	0.9141 (0.0328)	0.9423 (0.0287)	0.9423 (0.0287)	0.9375 (0.0260)	0.9793 (0.0159)

**DEE**	DEE.Abb	24	0.4976 (0.0582)	0.5313 (0.0521)	0.5701 (0.0511)	0.7180 (0.0526)	0.7101 (0.0484)
							
	DEE.Cer	39	0.6529 (0.0536)	0.6540 (0.0598)	0.6593 (0.0595)		0.7393 (0.0569)

**DERWENT**	DERW.Dash	25	0.0444 (0.0301)	0.0916 (0.0399)	0.2118 (0.0583)		0.4926 (0.0613)
							
	DERW.Mar	30	0.2230 (0.0506)	0.2617 (0.0571)	0.4020 (0.0610)	0.6065 (0.0651)	0.6299 (0.0616)
							
	DERW.New	32	0.4599 (0.0579)	0.4550 (0.0563)	0.5733 (0.0642)		0.6801 (0.0572)

**DOON**	DOON.Muc	30	0.6977 (0.0479)	0.6867 (0.0564)	0.7515 (0.0460)		0.7861 (0.0464)
							
	DOON.Nes	27	0.5924 (0.0575)	0.5767 (0.0580)	0.7433 (0.0481)	0.7978 (0.0435)	0.7612 (0.0444)
							
	DOON.Ske	29	0.2733 (0.0548)	0.3583 (0.0594)	0.4359 (0.0607)		0.5441 (0.0578)

**EDEN**	EDEN.Dac	30	0.5034 (0.0631)	0.5582 (0.0543)	0.7146 (0.0496)		0.9642 (0.0178)
							
	EDEN.Sca	31	0.5468 (0.0564)	0.4726 (0.0554)	0.5167 (0.0553)	0.7984 (0.0592)	0.8917 (0.0378)
							
	EDEN.Swin	30	0.4540 (0.0631)	0.5860 (0.0619)	0.7200 (0.0570)		0.9156 (0.0299)

**ELLE**	ELLE	50	0.3690 (0.0685)	0.4589 (0.0623)	0.4589 (0.0623)	0.4562 (0.0718)	0.9604 (0.0205)

**ELORN**	ELORN	49	0.5774 (0.0654)	0.6068 (0.0592)	0.6068 (0.0592)	0.6152 (0.0627)	0.9809 (0.0124)

**EO**	EO	46	0.9593 (0.0219)	0.9704 (0.0168)	0.9704 (0.0168)	0.9714 (0.0205)	0.9897 (0.0093)

**ESK (Border)**	ESKB.Boy	30	0.1651 (0.0531)	0.2192 (0.0477)	0.3084 (0.0581)		0.7579 (0.0506)
							
	ESKB.Ewe	30	0.1758 (0.0525)	0.1523 (0.0476)	0.2305 (0.0525)	0.5600 (0.0703)	0.7846 (0.0449)
							
	ESKB.Lid	35	0.6293 (0.0581)	0.6615 (0.0481)	0.6958 (0.0476)		0.9187 (0.0296)

**EWE**	EWE.Gru	26	0.3999 (0.0619)	0.4459 (0.0618)	0.4479 (0.0615)	0.6913 (0.0500)	0.8044 (0.0454)
							
	EWE.Kem	23	0.6749 (0.0537)	0.7443 (0.0471)	0.7510 (0.0475)		0.8560 (0.0358)

**EXE**	EXE.Dan	43	0.7485 (0.0528)	0.7621 (0.0487)	0.9266 (0.0282)		0.9808 (0.0138)
							
	EXE.Sher	31	0.3760 (0.0632)	0.4859 (0.0655)	0.7901 (0.0454)	0.9017 (0.0330)	0.9325 (0.0296)
							
	EXE.Sim	68	0.7348 (0.0576)	0.8304 (0.0443)	0.8720 (0.0393)		0.9513 (0.0254)

**FLEET**	FLE.Big	21	0.2120 (0.0499)	0.3837 (0.0589)	0.4322 (0.0593)	0.7521 (0.0532)	0.5670 (0.0615)
							
	FLE.Lit	26	0.7316 (0.0547)	0.7677 (0.0489)	0.8106 (0.0429)		0.8337 (0.0401)

**FOWEY**	FOW.Mar	20	0.5447 (0.0565)	0.6016 (0.0536)	0.6146 (0.0554)	0.6367 (0.0534)	0.8273 (0.0389)
							
	FOW.Tre	36	0.5255 (0.0570)	0.5423 (0.0548)	0.5448 (0.0539)		0.8574 (0.0412)

**GRUINARD**	GRU.Abh	27	0.1807 (0.0537)	0.2700 (0.0559)	0.2859 (0.0560)	0.6262 (0.0591)	0.6265 (0.0613)
							
	GRU.Ghi	27	0.5741 (0.0587)	0.5889 (0.0543)	0.6450 (0.0512)		0.7815 (0.0503)

**ITCHEN**	ITC.Bis	53	0.9215 (0.0381)	0.9644 (0.0232)	0.9644 (0.0232)	0.9608 (0.0222)	0.9975 (0.0047)

**KENT**	KENT.SpA	41	0.5339 (0.0607)	0.5975 (0.0638)	0.6246 (0.0634)	0.5048 (0.0669)	0.8821 (0.0344)
							
	KENT.Sto	20	0.0645 (0.0337)	0.0499 (0.0297)	0.2331 (0.0518)		0.7921 (0.0495)

**LAUNE**	LAU.Cot	47	0.5335 (0.0556)	0.6026 (0.0621)	0.6026 (0.0621)	0.5624 (0.0591)	0.7022 (0.0539)

**LAXFORD**	LAX.Ach	32	0.6540 (0.0598)	0.6882 (0.0572)	0.7423 (0.0553)		0.8579 (0.0420)
							
	LAX.Mai	30	0.2647 (0.0529)	0.3172 (0.0517)	0.3582 (0.0539)	0.8735 (0.0375)	0.6014 (0.0685)
							
	LAX.Thu	32	0.9983 (0.0039)	0.9978 (0.0045)	0.9978 (0.0045)		0.9986 (0.0035)

**LEUGER**	LEUGER	48	0.5531 (0.0588)	0.6086 (0.0652)	0.6086 (0.0652)	0.6205 (0.0666)	0.9235 (0.0287)

**LOCH LOCHY**	LOC.loc	46	0.5521 (0.0623)	0.6493 (0.0584)	0.6621 (0.0559)	0.7290 (0.0508)	0.7789 (0.0491)
							
	LOC.lun	26	0.4907 (0.0656)	0.4937 (0.0694)	0.6243 (0.0645)		0.7567 (0.0525)

**LOCH**	LOM.End	25	0.7578 (0.0490)	0.8568 (0.0397)	0.9377 (0.0239)	0.9631 (0.0192)	0.9478 (0.0240)

**LOMOND**	LOM.Fru	51	0.9356 (0.0262)	0.9613 (0.0184)	0.9639 (0.0182)		0.9712 (0.0169)

**LUNE**	LUNE.Birk	29	0.2499 (0.0577)	0.3227 (0.0517)	0.4072 (0.0622)		0.7938 (0.0471)
							
	LUNE.Cha	28	0.3762 (0.0573)	0.3617 (0.0677)	0.4067 (0.0650)	0.6959 (0.0593)	0.8240 (0.0396)
							
	LUNE.Ger	30	0.5511 (0.0525)	0.6270 (0.0512)	0.7186 (0.0568)		0.9407 (0.0276)

**MOY**	MOY.TriL	42	0.6995 (0.0537)	0.7281 (0.0564)	0.7281 (0.0564)	0.6681 (0.0511)	0.8209 (0.0461)

**NARCEA**	NARCEA02	40	0.5757 (0.0666)	0.6291 (0.0568)	0.6291 (0.0568)	0.5309 (0.0561)	0.9872 (0.0107)

**NITH**	NITH.RC	30	0.2519 (0.0578)	0.2875 (0.0613)	0.3588 (0.0656)		0.8590 (0.0347)
							
	NITH.NM	30	0.1596 (0.0478)	0.1928 (0.0521)	0.2553 (0.0558)	0.4859 (0.0658)	0.7314 (0.0608)
							
	NITH.NSC	30	0.6190 (0.0596)	0.6766 (0.0491)	0.6994 (0.0469)		0.9542 (0.0229)

**RIBBLE**	RIB.Bro	28	0.3664 (0.0503)	0.3255 (0.0517)	0.4399 (0.0593)		0.6874 (0.0577)
							
	RIB.Cra	29	0.5675 (0.0550)	0.5534 (0.0580)	0.7010 (0.0498)	0.7816 (0.0478)	0.9421 (0.0273)
							
	RIB.Ham	31	0.5400 (0.0624)	0.5979 (0.0623)	0.7223 (0.0484)		0.9008 (0.0355)

**SCORFF**	SCORFF	47	0.4018 (0.0710)	0.5002 (0.0710)	0.5002 (0.0710)	0.5077 (0.0700)	0.9778 (0.0137)

**SEE**	SEE	49	0.5916 (0.0623)	0.6705 (0.0633)	0.6705 (0.0633)	0.6607 (0.0613)	0.9613 (0.0177)

**SELLA**	SELLA.Pig	34	0.6253 (0.0576)	0.6967 (0.0538)	0.7406 (0.0510)	0.6630 (0.0643)	0.9934 (0.0088)
							
	SELLA02	48	0.3433 (0.0736)	0.4001 (0.0568)	0.4048 (0.0561)		0.9727 (0.0155)

**SELUNE**	SELUNE	50	0.5384 (0.0547)	0.6437 (0.0526)	0.6437 (0.0526)	0.6398 (0.0546)	0.9437 (0.0261)

**SEVERN**	SEV.CinA	22	0.5538 (0.0624)	0.5591 (0.0635)	0.5591 (0.0635)	0.4827 (0.0640)	0.9147 (0.0307)

**SUIR**	SUIR.Clo	28	0.2048 (0.0562)	0.1644 (0.0404)	0.2490 (0.0529)	0.5407 (0.0611)	0.5215 (0.0596)
							
	SUIR.MCb	20	0.2218 (0.0554)	0.2102 (0.0415)	0.6235 (0.0579)		0.8203 (0.0491)

**TAMAR**	TAM.Gat	33	0.3341 (0.0546)	0.3357 (0.0572)	0.5546 (0.0558)		0.8327 (0.0455)
							
	TAM.Ott	33	0.4564 (0.0619)	0.4843 (0.0534)	0.6979 (0.0541)	0.8110 (0.0544)	0.8377 (0.0409)
							
	TAM.Tre	30	0.6582 (0.0589)	0.6171 (0.0598)	0.7382 (0.0477)		0.8688 (0.0381)

**TAW**	TAW.Bra	25	0.5186 (0.0537)	0.6695 (0.0580)	0.6897 (0.0562)	0.5992 (0.0635)	0.9141 (0.0299)
							
	TAW.Twi	32	0.3199 (0.0651)	0.4196 (0.0648)	0.4226 (0.0655)		0.8506 (0.0408)

**TEIFI**	TEIFI.Cle	27	0.2314 (0.0515)	0.2581 (0.0529)	0.4018 (0.0586)		0.6037 (0.0577)
							
	TEIFI.Egn	24	0.3088 (0.0575)	0.4052 (0.0556)	0.6015 (0.0559)	0.6292 (0.0653)	0.8136 (0.0523)
							
	TEIFI.Lam	27	0.2229 (0.0485)	0.2310 (0.0557)	0.4185 (0.0600)		0.7051 (0.0566)

**TEST**	TEST	49	0.8644 (0.0432)	0.9642 (0.0240)	0.9642 (0.0240)	0.9676 (0.0232)	0.9962 (0.0060)

**TORRIDGE**	TOR.Eoak	21	0.2131 (0.0599)	0.3046 (0.0559)	0.3064 (0.0558)	0.8098 (0.0417)	0.7273 (0.0613)
							
	TOR.Woak	29	0.9248 (0.0274)	0.9362 (0.0237)	0.9384 (0.0234)		0.9765 (0.0162)

**ULLA**	ULLA	46	0.9350 (0.0308)	0.9641 (0.0195)	0.9641 (0.0195)	0.9610 (0.0245)	0.9996 (0.0018)

**USK**	USK.Bra	29	0.1643 (0.0497)	0.2160 (0.0544)	0.6023 (0.0651)		0.8644 (0.0357)
							
	USK.Gir	30	0.1840 (0.0467)	0.2293 (0.0501)	0.6600 (0.0558)	0.7371 (0.0609)	0.8865 (0.0412)
							
	USK.Grw	29	0.3297 (0.0646)	0.2726 (0.0531)	0.6865 (0.0629)		0.9321 (0.0276)

**WYE**	WYE.Lly(Wye)	30	0.4398 (0.0621)	0.4951 (0.0573)	0.7486 (0.0578)		0.9671 (0.0206)
							
	WYE.Edw	30	0.2290 (0.0517)	0.3178 (0.0664)	0.5506 (0.0666)	0.7413 (0.0622)	0.9389 (0.0274)
							
	WYE.Gar.	27	0.1242 (0.0497)	0.1532 (0.0521)	0.3548 (0.0624)		0.9280 (0.0304)

**Mean**			0.5029	0.5470	0.6366	0.7006	0.8441

The table shows: a) correct apportionment to individual sample sites within catchments with 10 loci (i.e. SSspG7 and Ssa197 removed); b) correct apportionment to individual sample sites within catchments with 12 loci; c) the sum of the apportionment to all samples in a catchment; d) the apportionment to catchment when all samples from within a catchment are pooled (i.e. the pool and allocate method); and e) the sum of apportionment to all samples in a reporting region. In the case of the individual sample simulations, a 108-sample baseline was used. One-hundred fish were used in the mixture sample, with 100 simulations of the mixture and baseline samples; an apportionment score of 1 = 100% correct. Individual samples are identified by tributary name; see Additional File 1 for details of latitude and longitude, collection date and individual sample size.

The key assumption in using simulations is that the baseline samples are representative of the populations present in the mixed stock fishery samples. Accordingly, the analysis of simulated single sample mixtures with ONCOR (Table 5) showed that estimates of stock composition were least accurate when samples were treated individually in construction of the baseline. The average allocation across all 108 samples to correct baseline sample was 0.55 and ranged between 0.05 with KENT_Stockdate_Beck to 1.0 with LAXFORD_Thull (1 being absolutely correct). To try and improve the accuracy, multiple samples within catchments, where present, were grouped together and the allocation to them was summed after mixture analysis (i.e. the allocate then sum method). This improved the estimates of stock composition and the average accuracy across all simulations increased to 0.64 (ranging from 0.21 with DERWENT_Dash_Beck to 1.0 with LAXFORD_Thull). The alternative strategy - pooling of allele frequencies from samples within the same catchment before allocation (i.e. the pool then allocate method) - also increased the average accuracy of estimates at the level of individual catchments to 0.70 (ranging between 0.39 for the Aulne to 1.0 for the Avon). Part of the improvement may be related to the reduction in the number of baseline samples that occurs when pooling samples in this way. However, the greatest improvement occurred where estimates were summed by reporting region (as defined in Fig. 2), which increased the average accuracy to 0.84 (ranging from 0.49 with the DERWENT_Dash_Beck sample to 1.0 with ULLA).

Simulated single sample mixtures were also produced for each of the baseline samples and analysed with SPAM (v. 3.7 [55]) using data from 12 loci (Additional File 2). Assignment success in SPAM was considerably higher than that demonstrated in ONCOR, which is consistent with reports that ONCOR is less prone to the over-estimation of predicted accuracy of MSA [54]. The SPAM results are presented here to facilitate comparison of our data and findings with a range of previously published studies, e.g. those focusing on Pacific salmon [18] and the west Greenland Atlantic salmon mixed stock fishery [29], but are not discussed further in the context of this paper.

### Analysis of Simulated Multi-Sample Mixtures

Four "fishery" mixture samples were simulated, and stock compositions were estimated at the level of individual sample sites and reporting regions (via the allocate and sum method). The accuracy of the estimated stock compositions for the specific samples sites were generally quite variable (Table 3), and there was a consistent tendency to underestimate the proportion each sample contributed to the mixture (the average estimate across all mixtures was 0.068, compared to an expected value of 0.125). It may also be noted that baseline samples from the northern areas tended to have the lowest levels of correct apportionment in the simulations; for example, mixture 4 (Table 3), which comprised only northern samples, produced the least accurate result (estimated stock compositions were on average 0.0683 away from the expected value for sample site and 0.0259 away from the expected value for reporting region). Despite these regional differences in the success of estimating stock compositions, the grouping of samples into broader reporting regions (Table 3, fourth column: 'Regional Estimate') improved the level accuracy of in almost every case. A similar pattern of results was also demonstrated when the proportion that each contributing stock made to the mixture was varied (Additional File 3), with a strong tendency to underestimate the true proportion of a contributing stock, but a much more reliable estimation at the level of reporting region.

### Analysis of Test Samples

Presuming that the test samples consist mainly of salmon originating from the catchments in which they were caught, provides a challenge for MSA with samples of known origin that are independent of the baseline. This allows the assumptions under which simulations were carried out to be assessed, namely that the baseline will be representative of all populations contributing to a mixture and that stocks omitted from the baseline will have genetic characteristics most similar to geographically proximate samples.

#### MSA of Test Samples with ONCOR

The results of the MSA run on the test samples in ONCOR are summarized in Table 6, and demonstrate a similar pattern of accuracy to that observed in the simulations. Attempts to apportion test samples to the level of individual river catchments were generally poor, regardless of the methods employed to pool baseline samples (Table 6, columns a and c), although the "pool and allocate" approach gave consistently higher estimates of stock contributions back to the river of capture (the average allocation across all seven test samples was 36.98% with the allocate and sum method and 45.69% with the pool and allocate method). The higher accuracy of the "pool and allocate" method probably reflects the small sample sizes of the baseline samples.

**Table 6:** Estimated percentage of the samples of returning adults of known origin that are apportioned back to proximate river and region of capture using ONCOR.

Test Sample ID	Test Sample	Capture Method	Sample Size	a) Allocation to river of capture	b) Assignment to river of capture	c) Allocation to pooled river of capture	d) Assignment to pooled river of capture	e) Allocation to region of capture	f) Assignment to region of origin
T1	Lune (2004-05)	Estuary Net	49	26.70 (2.17, 34.35)	28.57 (0.79, 0.48-0.98)	49.41 (12.8, 55.9)	53.01 (0.87, 0.51-1.00)	77.72 (45.53, 81.91)	81.63 (0.93, 0.53-1.00)

T2	Ribble 2004	Estuary Net	50	20.96 (1.64, 29.30)	22.00 (0.86, 0.59-1.00)	37.94 (3.2, 42.3)	40.00 (0.83, 0.48-1.00)	80.39 (45.86, 83.39)	82.00 (0.96, 0.56-1.00)

T3	Dee (1984-88)	Estuary Net	47	8.30 (0.00, 18.47)	8.51 (0.84, 0.55-1.00)	15.02 (0.0, 27.2)	17.02 (0.72, 0.34-1.00)	34.81 (18.11, 58.01)	34.04 (0.89, 0.55-1.00)

T4	Tamar 1987	Estuary Net	62	41.66 (11.03, 47.89)	41.94 (0.85, 0.28-1.00)	57.08 (25.1, 64.7)	65.08 (0.85, 0.39-1.00)	77.22 (48.23, 81.76)	80.65 (0.93, 0.53-1.00)

T5	Mudeford 2006	Estuary Net	47	81.24 (42.15, 89.13)	93.30 (0.79, 0.52-1.00)	88.50 (57.2, 95.9)	89.36 (0.96, 0.57-1.00)	98.47 (91.61, 1.00)	97.87 (1.00, 0.96-1.00)

T6	Aven+ 2005	Rod Caught	37	42.78 (8.0, 61.0)	40.54 (0.90, 0.52-1.00)	43.12 (8.3, 60.0)	40.54 (0.89, 0.52-1.00)	92.00 (75.18, 98.60)	91.89 (1.00, 0.98-1.00)

T7	Narcea 2006	Rod Caught	112	37.25 (22.7, 51.9)	39.29 (0.86, 0.51-1.00)	28.73 (14.3, 42.0)	28.57 (0.85, 0.47-0.99)	97.04 (91.02, 99.55)	96.43 (1.00, 0.97-1.00)

Mean				36.98	39.16	45.69	47.65	79.66	80.64

Estimates are given as: a) apportionment to correct river of capture, allocate and sum method (95% confidence intervals); b) assignment to river of capture (and, for those fish successfully assigned back to river of capture, the average and range for the probability of individual assignment is given in brackets); c) apportionment to pooled river of capture, i.e. the pool and allocate method (95% confidence intervals); d) assignment to pooled river of capture (and, for those fish successfully assigned back to river of capture, the average and range for the probability of individual assignment is given in brackets); e) as the sum of apportionment to all samples within a reporting region (95% confidence intervals); and f) as the sum of assignment to all samples within a reporting region (average and range for the probability of individual assignment). The geographical location of each test sample is given in Fig. 1; see Table 1 for full sample details (incl. latitude and longitude).

+A sample from the Aven (47°48'6" N, 3°44'7" W) was not included in the baseline, therefore values relate not to river of capture, but to another proximate river in Brittany, NW France, the Scorff.

The accuracy of the estimates improved most when summing allocations at the level of the reporting regions (Table 6, column e). Estimating stock composition of the net and rod catches against a regional baseline demonstrated levels of accuracy that, except for the Dee estuary sample, all exceeded 77% (with an average of 79.66%). This result is particularly significant in the case of the Aven rod-and-line sample as it is not represented in the baseline, yet the estimate of stock composition allocates the majority (92%) of the catch to the correct region of capture (in this case, northern France).

#### MSA of Test Samples with cBAYES

The estimated allocation back to catchment and reporting region of capture for the adult samples of known origin, using cBAYES, are detailed in Table 7. The application of the various methods for summing or pooling estimates at different hierarchical levels produced a similar pattern of outcomes with cBAYES as those obtained with ONCOR; however, levels of accuracy obtained with cBAYES were generally much higher (see Tables 6 and 7). The least accurate approach was to allocate and sum estimates at the level of river catchments; on average 34% of the test samples were apportioned to their river of capture, although the accuracy of apportionment was quite high with test samples from southern England, France and Spain (>87%), but the MCMC failed to converge with estimates for the other test samples and accuracy was generally much lower. The pooling of baseline samples from the same catchment, before allocation, dramatically increased the accuracy of estimates from the Dee (northern Wales) and Lune (northern England) estuary net samples, where correct apportionment rose to 46% and 74%, respectively (although the shrink factors still remained above 1.2 for the MCMC). Accordingly, the average level of correct apportionment rose to 56.44%. The most successful method was (as with the analysis using ONCOR) to allocate and sum estimates at the level of reporting regions; this approach indicated that salmon from the region of capture made up the predominant portion of a sample in all stock composition estimates (on average 93.24%). Thus, the principal allocation from each fishery sample to region of origin ranged from an apportionment of 99% of rod caught salmon from the river Narcea to northern Spain, to an apportionment of 77% of net caught salmon from the river Dee to the southwest England and Wales region. These results also support the outcomes from the simulations, namely that estimates of stock composition are most accurate at the level of reporting regions, and that pooling not summing samples within different catchments may produce more accurate estimates at this finer geographic scale.

**Table 7:** Estimated percentage of the test samples of returning adults that is apportioned back to proximate river and region of capture using cBAYES.

Test Sample ID	Test Sample	Allocation to River of Capture	Assignment to River of Capture	Allocation to Pooled River of Capture	Assignment to Pooled River of Capture	Allocation to Region of Capture	Assignment to Region of Origin
T1	Lune (2005-05)	15.751* (8.101)	13.725 (0.747, 0.4343-0.999)	74.391 (13.783)	88.235 (0.793, 0.440-0.999)	92.320 (6.200)	100

T2	Ribble (2004)	56.282* (24.379)	74.000 (0.658, 0.197-0.997)	66.488* (9.371)	76.000 (0.759, 0.279-1.000)	93.389 (5.153)	100

T3	Dee (1984-88)	3.563* (4.820)	2.041 (0.751, 0.290-0.998)	45.607* (15.734)	46.000 (0.668, 0.2682-0.998)	77.427 (9.189)	87.76

T4	Tamar (1987)	95.070* (3.860)	100.000 (0.857, 0.475-1.000)	84.513* (7.008)	90.000 (0.930, 0.670-1.000)	96.356 (3.472)	100

T5	Mudeford (2006)	97.601 (2.163)	100.000 (0.999, 0.991-1.000)	97.527 (2.172)	100 (0.999, 0.978-1.000)	97.802 (2.127)	100

T6	Aven+ (2005)	87.172 (5.885)	91.892 (0.961, 0.616-1.000)	86.438 (6.621)	91.892 (0.956, 0.506-1.000)	96.347 (3.260)	100

T7	Narcea (2006)	83.287 (5.207)	87.387 (0.919, 0.502-1.000)	85.819 (6.294)	91.892 (0.900, 0.515-1.000)	99.068 (0.875)	100

Mean		34.00	56.44	85.82	91.89	93.24	98.25

Estimates are given as the sum of apportionment to all samples within a catchment, the apportionment to catchment when all samples from within a catchment are pooled (i.e. the 'pool and allocate' method), and as the sum of apportionment to all samples within a reporting region (SDs of estimates are given in brackets). Individual assignment was also used to estimate the percent of test samples that would assign back to catchment and reporting region of capture (and from those fish successfully assigned back to river of capture the average and range for the probability of individual assignment is given in brackets). The geographical location of each test sample is given in Fig. 1; see Table 1 for full sample details (incl. latitude and longitude).

*Shrink factor exceeded 1.2 for estimates of stock allocation to one or more rivers.

+A sample from the Aven (47°48'6" N, 3°44'7" W) was not included in the baseline, therefore values relate not to river of capture, but to another proximate river in Brittany, NW France, the Scorff.

The use of cBAYES for estimating the stock proportions in some of the unpooled test fishery samples proved to be problematic as the MCMC would not converge when apportioning to individual sample sites or catchments (estimates were inconsistent between the chains and shrink factor exceeded 1.2, even when the chain length was increased to 2,000,000 iterations). This problem occurred with the Tamar, Dee, Ribble and Lune adult samples, but was not a problem with test samples from the southern regions included in the study. Despite this issue, the estimates of stock composition when utilizing cBAYES were generally more accurate, i.e. were more similar to the real composition of each test sample, than those generated with ONCOR, and failure of the MCMC to converge was generally not a problem for regional estimates of stock composition.

### Individual Assignment of Test Samples

The results from individual assignment of the fisheries test samples to baseline samples and catchments are given in Table 6 for analysis in ONCOR, and Table 7 for analysis in cBAYES. They show a similar pattern to the results of the MSA. Firstly, the sum and allocate to each catchment approach gave the least accurate results; across all seven test samples, the average assignment to river of capture was 36% in ONCOR and 56% in cBAYES. Secondly, the sum and allocate to region approach gave the most accurate results; average assignment was 81% in ONCOR and 98% in cBAYES, while the proportion of test samples assigned to catchment/region of capture was slightly higher than that estimated with MSA. Once again, the Bayesian method employed in cBAYES produced generally higher estimated allocation back to catchment and reporting region of capture. Finally, the level of assignment to catchment of capture and the average probability of assignment were much lower in fish from fisheries samples originating in the north of the study area compared to those sampled from the south.

## Discussion

The results of this work confirm the utility of MSA for the management and conservation of Atlantic salmon in Europe. Using twelve microsatellite loci and baseline samples of modest size, relatively accurate estimates of stock composition and apportioning of both simulated mixtures and net-fishery samples to region of origin have been achieved. Our findings also indicate large differences in the relative accuracy of stock composition estimates and MSA apportioning across the geographical range of the study, with a much higher degree of accuracy achieved when assigning and apportioning to populations in the south of the area studied. This result probably reflects the more genetically distinct nature of populations in the database from Spain, northwest France and southern England.

In recent work applying MSA to chinook salmon (*Oncorhynchus tshawytscha*) fisheries, Beacham *et al*. [60] were frequently able to demonstrate >90% accuracy of stock composition estimation in simulated single baseline sample mixtures using SPAM. Levels of accuracy obtained in the present study with SPAM and the reportedly more conservative ONCOR program were generally not as high as those of Beacham *et al*., and probably reflect the more extensive surveying and larger samples sizes in the baseline data collected for chinook salmon. Collection of samples in the current study was generally undertaken as part of routine in-river juvenile surveying. This helped to minimize disruption to wild Atlantic salmon populations, but made it difficult to increase sample sizes. Nevertheless, despite the generally smaller baseline sample sizes used in the current study, overall accuracy of MSA apportionment in SPAM was generally quite high (the lowest level of accuracy reported here is 78%, compared to <50% in the chinook salmon study [60]). This may reflect the bias SPAM demonstrates with small baseline sample size [56] and/or a greater degree of divergence between Atlantic salmon populations [65]. The application of ONCOR to the allocation of simulated mixtures generally produced much lower levels of accuracy. This suggests that bias in SPAM is indeed playing an important part in inflating accuracy in the current simulations and that this baseline is likely to give robust estimates for MSA only at broader geographic levels.

Assignment success, whether in terms of individual assignment or MSA apportionment is influenced by a range of interacting factors, including: genetic differentiation among populations, the number of baseline populations to be assigned to, the degree of polymorphism at each locus, the number of loci analysed, sample sizes; see Hansen *et al*. [66] for full details. In particular, the relationship between degree of divergence between populations and assignment success, as found in our study, has long been recognised and has been demonstrated in a range of empirical studies, e.g. [67-69], and in several landmark simulation/modelling papers [70-72]. Nevertheless, as work by Beacham *et al*. [60] demonstrates, the relationship is far from simplistic and, depending on the characteristics of the particular system being analysed, other factors, e.g. differences in number of alleles per locus, may be equally or indeed more important in determining accuracy of assignment.

Previous work has demonstrated the importance of baseline sample size to MSA; Beacham *et al*. [60] showed that a rapid increase in the accuracy of estimated stock composition occurred for samples sizes up to approximately 75 individuals. Similarly, Wood *et al*. [58] suggested that critical baseline sample size is around 40 individuals, below which the reliability of estimates is greatly reduced. In the current study, the majority of baseline sample sizes were below this recommended level (Additional File 1); therefore, pooling baseline samples that were collected from within the same catchment was undertaken as a method of increasing sample sizes. Pooling samples that demonstrate significant differences in allele frequencies could potentially introduce bias into a baseline, altering baseline allele frequencies and causing deviations from HWE, meaning that allele frequencies observed may not be truly representative of a population. For these reasons, samples were pooled within catchment, but not at the level of reporting regions; this ensured that within-catchment pooled sample sizes exceeded the critical levels of 40 to 75 individuals. In the current study, the effect of pooling samples generally increased the accuracy of the results and should be considered as a potential method for overcoming issues of small baseline samples in future studies. In part, the often small sample sizes reflect the difficulties and practicalities of undertaking such a wide-ranging, multi-agency study. Without doubt this has impacted negatively on the robustness of the database in its current form and has almost certainly played a large part in reducing the robustness of some of the statistical analyses undertaken; this effect was particularly marked in more northerly regions of the study area, where genetic differentiation between samples is already less distinct.

Generation of simulated baseline mixtures using ONCOR and SPAM facilitated comparison of our data with similar work on other salmonid species, whilst also allowing evaluation of strategies for pooling baseline samples. However, the accuracy of estimates of stock composition obtained with these two methods for a series of test samples was well below that achieved with a Bayesian algorithm in cBAYES. This was demonstrated by the increased allocation to catchment or region of capture in the majority of test samples when analysed with cBAYES, and supports similar findings in other studies [60]. The increased accuracy demonstrated by cBAYES did not, however, overcome the failure of the MCMC to converge when estimating the stock composition of some of the test samples. When attempting to apportion the test samples collected from many of the estuary nets in England to sample site or catchment the shrink factors in cBAYES exceeded 1.2, meaning the individual chains were producing differing estimates of stock composition. Failure of the MCMC to converge may reflect the inclusion of small baseline samples and/or the fact that some proximate samples were not sufficiently distinct from each other, having been sampled from the same population at different points in a river drainage. Issues of convergence were not a problem when estimating stock composition at the level of reporting regions, or with test samples from southern areas, which also tended to correspond to a higher levels of accuracy demonstrated in the simulations carried out in ONCOR and SPAM. Therefore, the results from simulations and test samples suggest there is insufficient power in the baseline data collected to date to reliably allocate fisheries samples to the level of individual sample sites or river catchments across the entire range of the study area.

It is interesting to note that whilst individual assignment is one of the most demanding tasks for stock identification methodologies, its application to the test samples was generally more accurate than the MSA (the proportion of the test samples allocated to the catchment/region of capture was generally slightly higher than that estimated in the MSA). This could reflect a bias in largely selecting test samples from rivers included in the baseline and may be related to the fact that in the MSA a small portion of each test sample was allocated to every one of the baseline samples, meaning that a 100% apportionment to a single catchment was never made. It could also be due to the fact that, whilst some individuals would be assigned to the catchment/region of capture, their probability of assignment could be low and this uncertainty is not necessarily reflected in the result.

The formation of reporting regions with the baseline samples also deserves further consideration. In the southern-most areas included in this study (i.e. Spain, France, southern England), well-supported clusters of samples are present in the dendrogram (Fig. 2), the MDS plot (Fig. 3) and the STRUCTURE analysis (Additional File 6). Similarly, in southwest England, Wales and southern Ireland, some distinct groups of samples can be identified, even if the support for these groups is not overly robust. This may be due in part to the somewhat discontinuous collection of samples in these regions, but still contrasts sharply with the population structure observed in more northern areas, where the grouping of samples into well-defined geographic reporting regions is less obvious and bootstrap support >50% generally only occurs in the most radial braches on the tree, i.e. supporting the grouping of samples collected from within an individual catchment. A lack of strong regional population structure in more northerly areas makes the grouping of samples difficult; specifically, the assumption that samples which are included in the baseline can act as surrogates for populations/catchments which have not been included, is called into question. Despite this, however, analysis of test samples from these areas was generally accurate to the reporting regions defined in the study (Tables 6 and 7). Failure of the MCMC to converge when running cBayes was also chiefly associated with samples in the northern area of the study, further emphasizing the importance of strong regional population structuring for genetic stock identification. At present, the regional groups identified in this study remain tentative and should be reviewed as and when additional data become available to supplement the baseline.

A reduced ability to evidence strong regional relationships between the northernmost samples included in the study (Scotland, Ireland, northwest England and Wales) could be due to a range of factors: insufficient sample size, the number of markers employed, a long history of stock transfers and salmon farming, or could reflect the underlying phylogeography of salmon in the region. Previous work into the phylogeography of Atlantic salmon in western Europe utilizing mitochondrial DNA found little association between geographic and genetic distance outside the Baltic Sea [73] and, more recently, the area of highest nucleotide diversity for the species has been located around the British Isles, prompting the suggestion that the area is acting as a zone of secondary contact between salmon recolonising from multiple glacial refugia [74]. If true, salmon from differentially colonized rivers, or even tributaries, could belong to different phylogeographic lineages accounting for the complex population structure around the British Isles [75,76]. The results of analysis of salmon in other parts of their range suggest that the phylogeographic origin of populations can have an important effect on the patterns of genetic diversity they exhibit [77-81]. Therefore, further study into the phylogeography of Atlantic salmon in Britain and Ireland could be extremely illuminating, particularly in areas that remained ice-free during the last glacial maximum, e.g. southern England and Ireland.

The temporal stability of the markers used in the analysis also need to be addressed; this factor is of critical importance in determining the length of time a baseline dataset remains useful for GSI. In the samples analysed for temporal stability in this study, none remained completely free of significant changes in allele frequencies at all loci, although most showed only a single locus with significant change and hierarchical analysis showed variation between sample sites was approximately four times greater than variation between temporal samples. Moreover, in the case of the estuary net test samples from the Dee and Tamar, where adult test fish were collected approximately 20 years before the baseline samples, MSA still showed predominant allocation back to the region of capture. Overall, these results suggest that genetic information within the baseline should remain useful for many years. Previous studies of temporal stability/instability of allele frequencies have produced conflicting results, including uncertainty as to how short-term stability translates into the longer-term [9,82-85]. Nevertheless, several detailed studies on Atlantic salmon [86-88] and brown trout [89] suggest that in general, variation between year classes is not significant [88] and that salmonid genetic population structure may remain stable over at least several decades [86,87,89]. This is obviously an area that will require further assessment and validation in the future. Accordingly, we anticipate that the length of time data remains useful will be river- and/or region-specific. In Pacific salmonids, where MSA has been carried out for decades, collection of samples across years is regarded as an important element in the on-going validation of such programmes [22,90].

Lastly, it is important to recognise that stocking, escapes from fish farms [91] and interchange of breeders between rivers may also account for some of the inability to assign individuals to a river or tributary. Repeated and intense stock transfers are known to have diluted between-river differentiation in Spanish rivers [92], and levels of introgression of alleles from northern regions as high as 11% have been reported for southern French salmon [93]. Thus, while the primary objective of this study was not to infer population structure, stock transfers may be an important contributing factor in reducing population differentiation and broader-scale patterns of isolation by distance, especially when stocking has occurred from very remote locations. As such, some inconsistencies and reductions in assignment power are almost certainly attributable to this source of genetic noise in the data. Nevertheless, while we have not formally addressed the issue of stocking within the current study, research conducted to date [94-96] indicates that due to a range of factors, including reduced fitness of stocked fish, introductions may have had little long-term effect in terms of contributing genes to extant populations. Moreover, while debate regarding the value of conserving populations whose genetic make-up has been compromised by introgression of alleles from stocked fish is important, from the perspective of this study, the samples collected are the best broad representation of the populations that currently produce marine migratory salmon from the southern part of the species' European range.

## Conclusions

This study represents the first time that Atlantic salmon from many of the rivers included in the baseline have been characterized genetically, and that salmon from a broad area across the south of the species European range have been analysed with a consistent set of microsatellite markers.

This sub-continental level of geographical coverage has shown the existence of regional genetic signatures in salmon, which appear to be independent of more commonly recognised terrestrial biogeographical and political boundaries. It is apparent that these regional genetic differences can affect the accuracy of MSA and indicate that to some degree the success of MSA will be region dependent. Specifically, our findings highlight large differences in the relative accuracy of stock composition estimates and MSA apportioning across the geographical range of the study, with a much higher degree of accuracy achieved when assigning and apportioning to populations in the south of the area studied. This result probably reflects the more genetically distinct nature of populations in the database from Spain, northwest France and southern England.

Validation of the ASAP baseline dataset for MSA of Atlantic salmon in southern Europe has proven successful, and the application of this methodology to rod-and-line and estuary net fisheries has produced realistic estimates of stock composition at a regional scale. However, it is clear that with the baseline assembled there is still potential for bias in estimates of stock composition, which can arise if a significant proportion of an analysed fishery originates from omitted or inadequately represented stocks. Therefore, additional sampling to increase the numbers of fish in each baseline sample, coupled with a broadening of the baseline to include more salmon rivers, particularly for example in Scotland, Ireland and Wales, will increase the accuracy and precision of analysis, while the inclusion of additional temporal samples will allow questions concerning the useful lifetime of baseline data for MSA to be addressed.

Broader questions concerning the individual origins of migratory salmon sampled in other regions, e.g. west Greenland [29], will require much broader baseline collections (or a radical re-thinking of how to identify baseline stocks outside of the Pacific salmonid/MSA model, perhaps through the identification of diagnostic markers [97]). However, this work reinforces the conclusion of Koljonen and coauthors in the Baltic Sea [24] that MSA is possible at a broad regional scale for Atlantic salmon and builds upon previous, more geographically limited, catchment-level applications of nuclear markers, which have also demonstrated accurate estimates of stock composition of salmon and trout catches in Europe [22,23]. Ultimately, the findings of the present study on Atlantic salmon, coupled with previous work on Pacific salmonids, reiterate the invaluable role of molecular markers in fisheries management.

## Authors' contributions

AMG carried out microsatellite optimisation and analysis, MSA and statistical genetic analysis, helped coordinate UK sample collection and drafted the manuscript. GM-S carried out microsatellite optimisation and analysis, MSA and statistical genetic analysis and helped draft the manuscript. ED and JC helped with MSA and statistical genetic analysis. JLH carried out microsatellite analysis. ED and JC helped with MSA and statistical genetic analysis AEB carried out MDS plot analyses. PM coordinated and directed sampling of specimens from rivers in Scotland. ST helped coordinate and direct sampling of specimens from rivers in southwest England, and coordinated collection of estuary and rod-and-line test fishery samples. WR and PG coordinated and undertook sampling of specimens from rivers in Ireland. TC and PMCG coordinated a parallel, intensive Irish MSA programme and TC contributed to the interpretation of the results and drafting of the manuscript. DB planned and wrote the project, undertook overall project coordination, and helped draft the manuscript. EG-V helped with technical planning of microsatellite analysis, project coordination, coordinated and directed sampling of specimens from rivers in Spain and helped draft the manuscript. JRS directed sample collection, technical planning of microsatellite analysis, project coordination and drafted the manuscript. All authors read and approved the final manuscript.

## Supplementary Material

Additional file 1 **Sample information**. Full details of all samples analysed in the study. Click here for file

Additional file 2 **Estimated proportion (plus S.E.) of the simulated single-population mixtures that is apportioned back to the correct baseline sample using SPAM**. Table shows (a) correct apportionment to individual sample sites within catchments, (b) the sum of the apportionment to all samples in a catchment, (c) the apportionment to catchment when all samples from within a catchment are pooled (i.e. the pool and allocate method), and (d) the sum of apportionment to all samples in a reporting region. In the individual sample simulations a 108 sample baseline was used. Click here for file

Additional file 3 **Multi-sample simulations in ONCOR using unequal proportions of baseline samples**. Four simulated mixtures are presented; each mixture comprised eight samples with a combination of unequal proportions making up each individual mixture, with mixtures 1 and 2 including a river from each reporting region, mixture 3 a combination of southern rivers and mixture 4 a combination of northern rivers. Stock compositions were estimated at the level of individual sample site and reporting region via the allocate and sum method. One-hundred fish were used in the mixture sample, with 100 simulations. Click here for file

Additional file 4 **Summary of population genetic statistics**. Summary statisitcs for all popualtions, including: sample size, number of alleles, observed and expected heterozygosities, and probability of conformance to Hardy-Weinberg equilibrium. Click here for file

Additional file 5 **Results of the analysis by MICROCHECKER**. MICRO-CHECKER analysis of every population (108 populations) at all 12 loci. Click here for file

Additional file 6 **STRUCTURE analysis of baseline samples**. STRUCTURE analysis [53], demonstrating clustering of baseline samples into regional groupings. Click here for file

Additional file 7 **Pairwise F_ST _values for all samples**. Pairwise comparison and calculation of F_ST _values between all samples. Tablewide significance levels were applied using the sequential Bonferroni procedure. Click here for file
