# Supplementary material for: Genetic stock identification of Atlantic salmon (Salmo salar) populations in the southern part of the European range
Source: BMC Genet. 2010 Apr 29;11:31. doi: 10.1186/1471-2156-11-31 (PMC2882343; doi:10.1186/1471-2156-11-31)
Supplement: Additional file 1 — Sample information. Full details of all samples analysed in the study. [file 1471-2156-11-31-S1.DOC]

**Additional File 1. Details of samples genotyped**

| **Catchment (Map Label)** | **Sample Site / Tributary Details** | **Latitude and Longitude** | **Sample Date** | **Sample Size** |
| --- | --- | --- | --- | --- |
| **Scotland** |  |  |  |  |
| BLACKWATER  (1) | Tarbert Burn, Blackwater | N 58:10:53 / 58.181436  W 06:39:56 / -6.665458 | 09/08/2005 | 33 |
| CREED  (2) | Allt Ruadh | N 58:11:56 / 58.198811  W 06:30:27 / -6.507374 | 12/08/2005 | 36 |
| LAXFORD  (3) | Achfary, Laxford | N 58:18:46 / 58.312655  W 04:54:43 / -4.912066 | 07/07/2005 | 32 |
| LAXFORD  (3) | Laxford | N 58:20:07 / 58.335313  W 04:54:13 / -4.903679 | 01/07/2005 | 30 |
| LAXFORD  (3) | Thull | N 58:24:59 / 58.416318  W 05:00:53 / -5.014819 | 27/07/2005 | 32 |
| GRUINARD  (4) | Abhainn,  Glen na Muice | N 57:45:46 / 57.762834  W 05:16:23 / -5.272942 | 24/10/2005 | 27 |
| GRUINARD  (4) | Ghiubhsachain Burn | N 57:48:34 / 57.809552  W 05:22:36 / -5.376533 | 24/10/2005 | 27 |
| EWE  (5) | Talladale,  Grudie bay, small tributaries at Loch Maree shore | N 57:40:34 / 57.676087  W 05:29:30 / -5.491621 | 20/09/2005 | 26 |
| EWE  (5) | Kemsary,  Loch Maree | N 57:45:17 / 57.754596  W 05:32:44 / -5.545441 | 07/09/2005 | 23 |
| LOCH LOCHY  (6) | Lochy  (below Loch Lochy) | N 56:54:38 / 56.910455  W 04:59:45 / -4.995820 | September 2005 | 46 |
| LOCH LOCHY  (6) | Lundy Tributary | N 56:50:50 / 56.847327  W 05:02:30 / -5.041619 | September 2005 | 26 |
| AWE  (7) | Clachan Dubh Tributary | N 56:08:34 / 56.142704  W 05:24:56 / -5.415619 | 30/08/2005 | 35 |
| AWE  (7) | Awe | N 56:26:02 / 56.433892  W 05:13:04 / -5.217796 | 21/10/2005 | 35 |
| AWE  (7) | Braevallich Tributary | N 56:13:01 / 56.216882  W 05:17:59 / -5.299601 | 08/08/2005 | 35 |
| LOCH LOMOND  (8) | Endrick | N 56:03:42 / 56.061598  W 04:20:47 / -4.346439 | Summer 2004 | 25 |
| LOCH LOMOND  (8) | Fruin | N 56:03:07 / 56.051878  W 04:43:53 / -4.731307 | Summer 2004 | 51 |
| CLYDE  (9) | Boclair Bridge, Allander Water, River Kelvin | N 55:55:40 / 55.927766 W 04:16:56 / -4.282311 | 17/10/2005 & 20/10/2005 | 30 |
| CLYDE  (9) | Cart, Loch Winnoch  River Calder, | N 55:47:47 / 55.796453  W 04:38:07 / -4.635204 | 19/08/2005 | 25 |
| AYR  (10) | Dalblair, Glenmuir Water | N 55:26:54 / 55.448276  W 04:08:44 / -4.145426 | 11/10/2004 | 30 |
| AYR  (10) | Howford Bridge | N 55:32:06 / 55.535095  W 04:05:18 / -4.088347 | 13/10/2004 | 30 |
| AYR  (10) | Lugar Water  (Near council) | N 55:27:52 / 55.464363  W 04:13:51 / -4.230934 | 22/07/2005 | 34 |
| AYR  (10) | Lugar Water  (Near council) | N 55:27:52 / 55.464363  W 04:13:51 / -4.230934 | October 2006 | 34 |
| DOON  (11) | Muck Water | N 55:19:25 / 55.323728  W 04:24:25 / -4.407004 | 08/09/2005 | 30 |
| DOON  (11) | Ness Glen | N 55:17:41 / 55.294857  W 04:24:00 / -4.400135 | 11/07/2005 | 27 |
| DOON  (11) | Skeldon Mills | N 55:23:37 / 55.393641  W 04:32:51 / -4.547524 | 08/09/2005 | 29 |
| CREE  (12) | Whitehills | N 55:01:04 / 55.017724  W 04:32:35 / -4.543173 | 20/07/2004 | 39 |
| FLEET  (13) | Big Water of Fleet | N 54:53:01/54.883587  W 04:11:02 / -4.183903 | 13/07/2004 | 21 |
| FLEET  (13) | Little Water of Fleet | N 54:53:01/54.883587  W 04:11:02 / -4.183903 | 13/07/2004 | 26 |
| NITH  (14) | River Cairn | N 55:09:08 / 55.152348  W 03:48:16 / -3.804450 | 05/10/2005 | 30 |
| NITH  (14) | Main river, Buccleuch Estate | N 55:06:48 / 55.113415  W 03:37:54 / -3.631716 | 06/10/2005 | 30 |
| NITH  (14) | Scaur Water | N 55:17:09 / 55.285815  W 03:55:46 / -3.929315 | 05/10/2005 | 30 |
| ANNAN  (15) | Birnock Water | N 55:19:56 / 55.332343  W 03:26:27 / -3.440743 | Summer 2005 | 30 |
| ANNAN  (15) | Evan Water | N55:23:48 / 55.396743  W3:32:22 / -3.539406 | Summer 2005 | 35 |
| ANNAN  (15) | Wamphray | N 55:14:48 / 55.246564  W 03:23:54 / -3.398312 | Summer 2005 | 31 |
| **England & Wales** |  |  |  |  |
| ESK (Border)  (16) | Liddel Water | N 55:16:36 / 55.276593  W 02:39:43 / -2.661872 | 31/08/2004 | 35 |
| ESK (Border)  (16) | Boyken Burn | N 55:11:24 / 55.190122  W 03:04:13 / -3.070396 | 20/08/2004 | 30 |
| ESK (Border)  (16) | Ewes Water | N 55:14:42 / 55.244895  W 02:58:05 / -2.968056 | 25/08/2004 | 30 |
| EDEN  (17) | Darce Beck | N 54:37:48 / 54.63003  W 02:52:22 / -2.872841 | 28/08/2004 | 30 |
| EDEN  (17) | Scandal Beck | N 54:28:18 / 54.471619  W 02:24:35 / -2.409637 | 15/08/2004 | 31 |
| EDEN  (17) | Swindale Beck | N5 4:31:46 / 54.529413  W 02:43:54 / -2.731621 | 20/08/2004 | 30 |
| DERWENT (18) | Dash Beck | N 54:40:49 / 54.680320  W 03:11:36 / -3.193426 | 21/08/2004 | 25 |
| DERWENT  (18) | Marron | N 54:37:28 / 54.624377  W 03:27:35 / -3.459751 | 26/08/2004 | 30 |
| DERWENT  (18) | Newlands Beck | N 54:35:45 / 54.595970  W 03:10:48 / -3.180123 | 21/08/2004 | 32 |
| KENT  (19) | Sprint,  N.W. Kent | N 54:26:31 / 54.442016  W 02:47:52 / -2.797800 | 05/08/2004 | 41 |
| KENT  (19) | Stockdate Beck | N 54:26:26 / 54.440610  W 02:47:10 / -2.786116 | 17/08/2004 | 20 |
| LUNE  (20) | Birk Beck, Bretherale | N 54:26:34 / 54.442883  W 02:36:57 / -2.615972 | 11/10/2004 | 29 |
| LUNE  (20) | Luffman Farm, Howgill,  Chapel Beck | N 54:20:52 / 54.347852  W 02:34:06 / -2.568398 | 01/10/2004 | 28 |
| LUNE  (20) | A65 Bridge, Ingleton,  River Greta | N 54:08:59 / 54.149621  W 02:28:42 / -2.478408 | 13/10/2004 | 30 |
| RIBBLE  (21) | Broadsden Farm Hill,  River Hodder | N 53:56:44 / 53.945555  W 02:29:23 / -2.489781 | 19/07/2004 | 29 |
| RIBBLE  (21) | Cragg Hill Farm River Ribble | N 54:07:56 / 54.132233  W 02:17:46 / -2.296092 | 02/09/2004 | 29 |
| RIBBLE  (21) | Hammerton Hall, River Hodder | N 53:58:38 / 53.977228  W 02:26:01 / -2.433739 | 17/08/2004 | 31 |
| DEE  (22) | Abbey Brook | N 52:59:05 / 52.984764  W 03:11:08 / -3.185561 | 07/07/2005 | 24 |
| DEE  (22) | Ceiriog | N 52:54:46 / 52.912731  W 03:11:49 / -3.196976 | 07/07/2005 | 40 |
| TEIFI  (23) | Clettwr, Dolbantan | N 52:02:36 / 52.043290  W 04:16:05 / -4.268041 | 06/10/2005 | 27 |
| TEIFI  (23) | Nant Egnant | N 52:16:59/52.283170  W 03:46:57 / -3.782533 | 23/06/2005 | 24 |
| TEIFI  (23) | Lampeter | N 52:06:32 / 52.108934  W 04:04:22 / -4.072757 | 06/10/2005 | 27 |
| USK  (24) | River Bran | N 51:57:25 /51.956932  W 03:28:45 /-3.479218 | 13/08/2004 | 29 |
| USK  (24) | River Ysgir | N 51:57:55 / 51.965383  W 03:27:01 / -3.450384 | 13/08/2004 | 30 |
| USK  (24) | River Grwyrn | N 51:51:27 / 51.857510  W 03:07:07 / -3.118739 | 23/08/2004 | 29 |
| WYE  (25) | River Edw | N 52:07:48 / 52.129871  W 03:18:03 / -3.300861 | 13/09/2004 | 30 |
| WYE  (25) | River Llynfi | N 52:01:19 / 52.021953  W 03:13:08 / -3.219027 | 26/08/2004 | 30 |
| WYE  (25) | Garth Dulas | N 52:09:08 / 52.152149  W 03:32:28 / -3.541226 | 21/09/2004 | 27 |
| SEVERN  (26) | Cinderford Brook | N 51:46:59 / 51.783102  W 02:29:17 / -2.487996 | 03/09/2004 | 22 |
| TAW  (27) | Bray, Leworthy | N 51:07:35 / 51.126419  W 03:53:29 / -3.891255 | 08/09/2004 | 25 |
| TAW  (27) | Twitchen Stream, West Molland | N 51:02:05 /51.034608  W 03:43:19 /-3.722065 | 01/09/2004 | 32 |
| TORRIDGE  (28) | A30 Road Bridge, East Oakement | N 50:44:11 / 50.736378  W 03:58:59 / -3.983204 | 20/09/2004 | 21 |
| TORRIDGE  (28) | Oakhampton Castle,  West Oakement | N 50:43:53 / 50.731497  W 04:00:20 / -4.005671 | 16/09/2005 | 29 |
| CAMAL  (29) | Key Bridge,  De Lank | N 50:31:57 / 50.532555  W 04:42:07 / -4.701873 | 26/07/2005 | 30 |
| CAMAL  (29) | Gam | N 50:34:10 / 50.569490  W 04:41:59 / -4.699849 | 12/08/2005 | 30 |
| CAMAL  (29) | Kenning-stock | N 50:35:50 / 50.597245  W 04:41:24 / -4.689892 | 25/07/2005 | 30 |
| FOWEY  (30) | Margate Ford | N 50:28:06 / 50.468443  W 04:40:45 / -4.679175 | 15/08/2005 | 20 |
| FOWEY  (30) | Treverbyn | N 50:28:50 / 50.480568  W 04:31:46 / -4.529337 | 16/08/2004 | 36 |
| TAMAR  (31) | Gatherly, Lyd | N 50:38:03 / 50.634071  W 04:17:31 / -4.292062 | 08/08/2003 | 33 |
| TAMAR  (31) | Bealsmill, Inny | N 50:34:13 / 50.570155  W 04:19:03 / -4.317627 | 15/07/2003 | 33 |
| TAMAR  (31) | Trengune, Ottery | N 50:42:35 / 50.709844  W 04:33:43 / -4.562061 | 14/08/2003 | 30 |
| DART  (32) | Postbridge,  East Dart | N 50:35:44 / 50.595439  W 03:54:49 / -3.913689 | 03/08/2006 | 39 |
| DART  (32) | Postbridge,  East Dart | N 50:35:44 / 50.595439  W 03:54:49 / -3.913689 | 22/08/2005 | 45 |
| EXE  (33) | Slade Bridge, Danes Brook | N 51:03:33 / 51.059267  W 03:37:59 / -3.633077 | 14/07/2004 | 43 |
| EXE  (33) | Fernyball,  Sherdon Water | N 51:06:21 / 51.105823  W 03:42:38 / -3.710426 | 12/07/2004 | 31 |
| EXE  (33) | Simonsbath, Barle | N 51:08:22 / 51.139342  W 03:45:16 / -3.754550 | 01/07/2004 | 30 |
| EXE (33) | Simonsbath, Barle | N 51:08:22 / 51.139342  W 03:45:16 / -3.754550 | 04/08/2005 | 38 |
| AVON (Hampshire) (34) | Avon Bridge, Upper Avon | N 51:05:46 / 51.096112  W 01:49:06 / -1.818290 | 07/10/2004 | 23 |
| AVON (Hampshire) (34) | Bugmoor Hatches | N 51:00:28 / 51.007847  W 01:47:14 / -1.787263 | 14/10/2004 | 20 |
| ITCHEN (35) | Bishopstoke Barge | N 50:57:57 / 50.965754  W 01:20:16 / -1.337858 | 19/09/2005 | 29 |
| ITCHEN (35) | Bishopstoke Barge | N 50:57:57 / 50.965754  W 01:20:16 / -1.337858 | August 2006 | 24 |
| TEST (36) | Oakley | N 51:02:56 / 51.048915  W 01:31:44 / -1.528935 | 15/07/2004 | 1 |
| TEST (36) | Mottisfont | N 51:02:27 / 51.040753  W 01:32:03 / -1.534210 | 15/07/2004 | 7 |
| TEST (36) | Sheepbridge | N 51:05:05 / 51.084742  W 01:30:44 / -1.512096 | 17/07/2004 | 3 |
| TEST (36) | Kimbridge | N 51:02:09 / 51.035729  W 01:32:06 / -1.535016 | 15/07/2004 | 3 |
| TEST (36) | Kimbridge | N 51:02:09 / 51.035738  W 01:32:06 / -1.535016 | 15/07/2004 | 1 |
| TEST (36) | North Head | N 51:05:18 / 51.088239  W 01:30:34 / -1.509532 | 14/07/2004 | 1 |
| TEST (36) | Compton | N 51:03:53 / 51.064677  W 01:31:28 / -1.524337 | 16/07/2004 | 3 |
| TEST (36) | Memorial Park | N 50:59:15 / 50.987364  W 01:30:19 / -1.505267 | 26/07/2004 | 10 |
| TEST (36) | Linhay Meads | N 51:01:30 / 51.025062  W 01:31:31 / -1.525398 | 12/08/2004 | 1 |
| TEST (36) | Moorcourt Carrier | N 50:57:14 / 50.953838  W 01:29:47 / -1.496397 | 25/08/2004 | 20 |
| **France** |  |  |  |  |
| SEE  (37) | Across the catchment | River mouth:  N 48:40:08 / 48.668889  W 01:24:19 / -1.405278 | 13/03/2005 - 28/08/2005 | 50 |
| SELUNE  (38) | Across the catchment | River mouth:  N 48:38:59 / 48.649722  W 01:23:19 / -1.388611 | 12/03/2005 - 12/08/2005 | 50 |
| LEGUER  (39) | Across the catchment | River mouth:  N 48:43:45 / 48.729167  W 03:33:14 / -3.553889 | 07/03/2005 - 24/07/2005 | 49 |
| ELORN  (40) | Across the catchment | River mouth:  N 48:23:14 / 48.387222  W 04:24:01 / -4.400278 | 12/03/2005 - 29/08/2005 | 49 |
| AULNE  (41) | Across the catchment | River mouth:  N 48:18:12 / 48.303333  W 04:16:17 / -4.271389 | 12/03/2005 - 15/10/2005 | 39 |
| ELLE  (42) | Across the catchment | River mouth:  N 47:52:20 / 47.872222  W 03:32:42 / -3.545000 | 12/03/2005 - 11/10/2005 | 50 |
| SCORFF  (43) | Across the catchment | River mouth:  N 47:44:37 / 47.743611  W 03:20:54 / -3.348333 | 12/04/2005 - 16/10/2005 | 48 |
| BLAVET  (44) | Across the catchment | River mouth:  N 47:44:19 / 47.738611  W 03:20:11 / -3.336389 | 12/03/2005 - 29/07/2005 | 49 |
| NIVELLE  (45) | Across the catchment | River mouth:  N 43:23:15 / 43.3875  W 01:40:09 / -1.669167 | Summer 2004 | 50 |
| **Spain** |  |  |  |  |
| ASÓN  (46) | Across the catchment | River mouth:  N 43:23:45 / 43.395833  W 03:26:55 / -3.448611 | Summer 2004 | 49 |
| CARES  (47) | Across the catchment | River mouth:  N 43:19:00 / 43.151724  W 04:36:00 / -4.918442 | Summer 2004 | 25 |
| CARES  (47) | Across the catchment | River mouth:  N 43:19:00 / 43.151724  W 04:36:00 / -4.918442 | Spring  2002 | 50 |
| CARES (47) | Casaño | N 43:18:00 / 43.274206  W 04:37:00 / -4.957924 | Summer 2004 | 25 |
| SELLA (48) | Across the catchment | River mouth:  N 43:28:30 / 43.128301  W 05:04:30 / -5.025902 | Spring  2002 | 50 |
| SELLA (48) | Across the catchment | River mouth:  N 43:28:30 / 43.128301  W 05:04:30 / -5.025902 | Summer 2004 | 50 |
| SELLA (48) | Piloña | N 43:28:00 / 43.340800  W 05:06:00 / -5.334830 | Summer 2004 | 35 |
| NARCEA (49) | Across the catchment | River mouth:  N 43:28:00 / 43.4670  W 06:07:00 / -6.1170 | Summer 2004 | 50 |
| NARCEA  (49) | Across the catchment | River mouth:  N 43:28:00 / 43.4670  W 06:07:00 / -6.1170 | Spring  2002 | 50 |
| EO  (50) | Across the catchment | River mouth:  N 43:32:00 / 43.5333  W 07:01:30 / -7.0233 | Summer 2004 | 46 |
| ULLA  (51) | Across the catchment | River mouth  N 42:39:00 / 42.6500  W 08:44:30 / -8.7333 | Summer 2004 | 46 |
| **Ireland** |  |  |  |  |
| MOY  (52) | Trimoge, Kilkelly | N 53:52:12 / 53.869886  W 08:50:60 / -8.8498937 | 25/08/2004 | 42 |
| LAUNE  (53) | Cottoners | N 52:04:03 / 52.067549  W 08:38:35 / -8.643161 | 12/10/2004 | 47 |
| CORK BLACKWATER  (54) | Awnaskirtaun | N 52:03:54 / 52.065122  W 09:12:43 / -9.211922 | 20/10/2004 | 35 |
| CORK BLACKWATER  (54) | Clydagh | N 52:04:03 / 52.067549  W 08:38:35 / -8.643161 | 21/10/2004 | 35 |
| CORK BLACKWATER  (54) | Glen | N 52:06:54 / 52.115135  W 08:53:16 / -8.887664 | 21/10/2004 | 35 |
| BARROW  (55) | Ballyclare Bridge, Barrow | N 52:06:54 / 52.115135  W 08:53:16 / -8.887664 | 08/10/2004 | 40 |
| SUIR  (56) | Clodiagh | N 52:37:35 / 52.626501  W 07:55:29 / -7.924677 | 10/08/2004 | 28 |
| SUIR  (56) | Beakstown | N 52:39:09 / 52.652506  W 07:51:40 / -7.861087 | 11/08/2004 | 20 |
| BOYNE (57) | Raharney, Deel | N 53:31:24 / 53.523466  W 07:05:36 / -7.093464 | 28/09/2004 | 36 |
| BOYNE (57) | Borora, Moynalty | N 53:48:31 / 53.808631  W 06:54:43 / -6.912044 | 27/09/2004 | 35 |
| BOYNE (57) | Skane Lwr | N 53:36:24 / 53.606638  W 06:38:26 / -6.640565 | 27/09/2004 | 35 |
