# Supplementary material for: Genetic stock identification of Atlantic salmon (Salmo salar) populations in the southern part of the European range
Source: BMC Genet. 2010 Apr 29;11:31. doi: 10.1186/1471-2156-11-31 (PMC2882343; doi:10.1186/1471-2156-11-31)
Supplement: Additional file 2 — Estimated proportion (plus S.E.) of the simulated single-population mixtures that is apportioned back to the correct baseline sample using SPAM. Table shows (a) correct apportionment to individual sample sites within catchments, (b) the sum of the apportionment to all samples in a catchment, (c) the apportionment to catchment when all samples from within a catchment are pooled (i.e. the pool and allocate method), and (d) the sum of apportionment to all samples in a reporting region. In the individual sample simulations a 108 sample baseline was used. [file 1471-2156-11-31-S2.DOC]

**Additional File 2. Estimated proportion (plus Standard Error) of the simulated single-population mixtures that is apportioned back to the correct baseline sample (1 = correct) using SPAM.**

| Catchment | Tributary | a) Sample Allocation | b) Catchment Allocation | c) Pooled Catchment Allocation | d) Regional Allocation |
| --- | --- | --- | --- | --- | --- |
| BLACKWATER  (Scotland) | Tarbert Burn | 0.8637 (0.0406) | 0.8637 (0.0406) | 0.8403 (0.0430) | 0.9018 (0.0345) |
| CREED | Allt Ruadh | 0.8818 (0.0328) | 0.8818 (0.0328) | 0.8512 (0.0378) | 0.9056 (0.0296) |
| LAXFORD | Achfary | 0.8520 (0.0376) | 0.8662 (0.0361) |  | 0.9020 (0.0293) |
|  | Laxford | 0.8512 (0.0363) | 0.8540 (0.0364) | 0.9293 (0.0292) | 0.8904 (0.0317) |
|  | Thull | 0.8897 (0.0351) | 0.8966 (0.0329) |  | 0.9109 (0.0302) |
| EWE | Talladale | 0.8282 (0.0416) | 0.8287 (0.0415) | 0.8872 (0.0338) | 0.8919 (0.0337) |
|  | Kemsary | 0.8567 (0.0342) | 0.8582 (0.0346) |  | 0.8970 (0.0303) |
| GRUINARD | Abhainn | 0.8175 (0.0439) | 0.8192 (0.0435) | 0.8779 (0.0410) | 0.8721 (0.0375) |
|  | Ghiubhsachain | 0.8547 (0.0399) | 0.8615 (0.0399) |  | 0.8916 (0.0341) |
| LOCH LOCHY | Lochy | 0.8939 (0.0315) | 0.8946 (0.0310) | 0.8982 (0.0341) | 0.9159 (0.0282) |
|  | Lundy | 0.8207 (0.0435) | 0.8441 (0.0425) |  | 0.8798 (0.0382) |
| AWE | Clachan Dubh | 0.9156 (0.0322) | 0.9176 (0.0318) |  | 0.9236 (0.0306) |
|  | Awe | 0.8918 (0.0314) | 0.8927 (0.0311) | 0.9629 (0.0212) | 0.9005 (0.0301) |
|  | Braevallich | 0.9213 (0.0273) | 0.9247 (0.0265) |  | 0.9311 (0.0269) |
| LOCH LOMOND | Endrick | 0.8649 (0.0378) | 0.8789 (0.0388) | 0.9425 (0.0266) | 0.8969 (0.0364) |
|  | Fruin | 0.9264 (0.0219) | 0.9278 (0.0217) |  | 0.9372 (0.0217) |
| CLYDE | Boclair Bridge | 0.8765 (0.0327) | 0.8769 (0.0327) | 0.8936 (0.0343) | 0.8906 (0.0336) |
|  | Cart | 0.8639 (0.0367) | 0.8655 (0.0367) |  | 0.8816 (0.0328) |
| AYR | Dalblair | 0.8366 (0.0373) | 0.9210 (0.0275) | 0.9537 (0.0242) | 0.9255 (0.0269) |
|  | Howford | 0.8334 (0.0418) | 0.8679 (0.0375) | 0.9537 (0.0242) | 0.8749 (0.0380) |
|  | Lugar Water | 0.9324 (0.0276) | 0.9439 (0.0241) |  | 0.9484 (0.0218) |
| DOON | Muck Water | 0.8874 (0.0327) | 0.8957 (0.0321) |  | 0.9225 (0.0277) |
|  | Ness Glen | 0.8564 (0.0366) | 0.8799 (0.0312) | 0.9229 (0.0306) | 0.9188 (0.0266) |
|  | Skeldon Mills | 0.8522 (0.0364) | 0.8606 (0.0364) |  | 0.9026 (0.0335) |
| FLEET | Big Water | 0.7992 (0.0500) | 0.8034 (0.0503) | 0.8872 (0.0358) | 0.8288 (0.0491) |
|  | Little Water | 0.8432 (0.0431) | 0.8494 (0.0413) |  | 0.8641 (0.0387) |
| CREE | Whitehills | 0.8867 (0.0397) | 0.8867 (0.0397) | 0.8544 (0.0427) | 0.9003 (0.0376) |
| NITH | Cairn | 0.8383 (0.0460) | 0.8460 (0.0440) |  | 0.9296 (0.0300) |
|  | Main river | 0.8078 (0.0478) | 0.8187 (0.0451) | 0.8589 (0.0463) | 0.9072 (0.0370) |
|  | Scaur Water | 0.8470 (0.0413) | 0.8595 (0.0402) |  | 0.9451 (0.0231) |
| ANNAN | Birnock Water | 0.8634 (0.0360) | 0.8742 (0.0331) |  | 0.9269 (0.0265) |
|  | Evan Water | 0.8566 (0.0357) | 0.8632 (0.0358) | 0.8753 (0.0415) | 0.9385 (0.0229) |
|  | Wamphray | 0.8206 (0.0440) | 0.8358 (0.0434) |  | 0.9161 (0.0325) |
| ESK (Border) | Liddel Water | 0.8692 (0.0369) | 0.8786 (0.0361) |  | 0.9427 (0.0250) |
|  | Boyken Burn | 0.8182 (0.0420) | 0.8321 (0.0392) | 0.8770 (0.0400) | 0.9145 (0.0267) |
|  | Ewes Water | 0.8263 (0.0432) | 0.8370 (0.0406) |  | 0.9193 (0.0298) |
| EDEN | Darce Beck | 0.8667 (0.0361) | 0.8788 (0.0353) |  | 0.9496 (0.0215) |
|  | Scandal Beck | 0.8588 (0.0368) | 0.8670 (0.0355) | 0.9231 (0.0276) | 0.9386 (0.0252) |
|  | Swindale Beck | 0.8720 (0.0377) | 0.8865 (0.0359) |  | 0.9487 (0.0227) |
| DERWENT | Dash Beck | 0.7813 (0.0480) | 0.7949 (0.0476) |  | 0.8598 (0.0383) |
|  | Marron | 0.8296 (0.0430) | 0.8411 (0.0424) | 0.8725 (0.0370) | 0.8990 (0.0339) |
|  | Newlands Beck | 0.8456 (0.0401) | 0.8608 (0.0392) |  | 0.8987 (0.0307) |
| KENT | Sprint | 0.8848 (0.0359) | 0.8879 (0.0350) | 0.8619 (0.0428) | 0.9430 (0.0251) |
|  | Stockdate Beck | 0.7531 (0.0525) | 0.7722 (0.0503) |  | 0.8836 (0.0322) |
| LUNE | Birk Beck | 0.8393 (0.0403) | 0.8474 (0.0399) |  | 0.9182 (0.0313) |
|  | Chapel Beck | 0.8306 (0.0435) | 0.8382 (0.0418) | 0.9026 (0.0359) | 0.9120 (0.0319) |
|  | Greta | 0.8882 (0.0356) | 0.8979 (0.0348) |  | 0.9477 (0.0239) |
| RIBBLE | Broadsden Farm | 0.8546 (0.0416) | 0.8645 (0.0400) |  | 0.9182 (0.0329) |
|  | Cragg Hill Farm | 0.8596 (0.0366) | 0.8759 (0.0352) | 0.9293 (0.0332) | 0.9508 (0.0244) |
|  | Hammerton Hall | 0.8546 (0.0348) | 0.8741 (0.0302) |  | 0.9367 (0.0220) |
| DEE | Abbey Brook | 0.8526 (0.0449) | 0.8578 (0.0432) | 0.8972 (0.0302) | 0.8932 (0.0356) |
|  | Ceiriog | 0.8820 (0.0397) | 0.8839 (0.0401) |  | 0.9251 (0.0305) |
| TEIFI | Clettwr | 0.8398 (0.0414) | 0.8486 (0.0437) |  | 0.8892 (0.0387) |
|  | Nant Egnant | 0.8266 (0.0414) | 0.8464 (0.0360) | 0.8968 (0.0346) | 0.8966 (0.0328) |
|  | Lampeter | 0.8395 (0.0406) | 0.8515 (0.0401) |  | 0.8998 (0.0346) |
| USK | Bran | 0.8167 (0.0445) | 0.8528 (0.0396) |  | 0.9100 (0.0310) |
|  | Ysgir | 0.8259 (0.0390) | 0.8632 (0.0356) | 0.9118 (0.0377) | 0.9160 (0.0284) |
|  | Grwyrn | 0.8422 (0.0455) | 0.8846 (0.0347) |  | 0.9344 (0.0287) |
| WYE | Edw | 0.8545 (0.0420) | 0.8763 (0.0371) |  | 0.9432 (0.0263) |
|  | Llynfi | 0.8552 (0.0411) | 0.8806 (0.0369) | 0.9059 (0.0330) | 0.9473 (0.0223) |
|  | Garth Dulas | 0.8045 (0.0441) | 0.8292 (0.0404) |  | 0.9211 (0.0298) |
| SEVERN | Cinderford Br’k | 0.8135 (0.0471) | 0.8135 (0.0471) | 0.7752 (0.0530) | 0.9048 (0.0307) |
| TAW | Bray | 0.8641 (0.0389) | 0.8689 (0.0395) | 0.8714 (0.0438) | 0.9237 (0.0291) |
|  | Twitchen | 0.8449 (0.0404) | 0.8456 (0.0402) |  | 0.9255 (0.0271) |
| TORRIDGE | East Oakement | 0.7757 (0.0409) | 0.7761 (0.0411) | 0.8951 (0.0330) | 0.8633 (0.0330) |
|  | West Oakement | 0.8700 (0.0350) | 0.8717 (0.0355) |  | 0.9301 (0.0273) |
| CAMAL | De Lank | 0.8641 (0.0352) | 0.8679 (0.0352) |  | 0.9253 (0.0309) |
|  | Gam | 0.8495 (0.0343) | 0.8571 (0.0343) | 0.9048 (0.0287) | 0.9064 (0.0296) |
|  | Kenning-stock | 0.8286 (0.0442) | 0.8394 (0.0414) |  | 0.9133 (0.0327) |
| FOWEY | Margate Ford | 0.8340 (0.0390) | 0.8383 (0.0395) | 0.8769 (0.0433) | 0.9008 (0.0301) |
|  | Treverbyn | 0.8552 (0.0419) | 0.8556 (0.0418) |  | 0.9171 (0.0311) |
| TAMAR | Gatherly | 0.8611 (0.0366) | 0.8760 (0.0359) |  | 0.9287 (0.0276) |
|  | Inny | 0.8663 (0.0363) | 0.8866 (0.0331) | 0.9237 (0.0347) | 0.9205 (0.0289) |
|  | Trengune | 0.8622 (0.0350) | 0.8789 (0.0320) |  | 0.9220 (0.0258) |
| DART | Postbridge | 0.9605 (0.0178) | 0.9605 (0.0178) | 0.9534 (0.0238) | 0.9794 (0.0138) |
| EXE | Danes Brook | 0.9020 (0.0286) | 0.9351 (0.0224) |  | 0.9672 (0.0176) |
|  | Sherdon Water | 0.8324 (0.0360) | 0.8895 (0.0293) | 0.9487 (0.0265) | 0.9409 (0.0207) |
|  | Barle | 0.9334 (0.0259) | 0.9402 (0.0245) |  | 0.9628 (0.0189) |
| AVON | Avon Bridge | 0.8077 (0.0415) | 0.8495 (0.0390) | 0.8989 (0.0334) | 0.9064 (0.0321) |
|  | Bugmoor | 0.7950 (0.0400) | 0.8249 (0.0390) |  | 0.8800 (0.0303) |
| ITCHEN | Bishopstoke | 0.9347 (0.0284) | 0.9347 (0.0284) | 0.9261 (0.0301) | 0.9492 (0.0231) |
| TEST | COMBINED | 0.9295 (0.0281) | 0.9295 (0.0281) | 0.9187 (0.0275) | 0.9532 (0.0228) |
| SEE | Catchment-wide | 0.9098 (0.0294) | 0.9098 (0.0294) | 0.9021 (0.0336) | 0.9569 (0.0191) |
| SELUNE | Catchment-wide | 0.9091 (0.0290) | 0.9091 (0.0290) | 0.9003 (0.0332) | 0.9555 (0.0198) |
| LEUGER | Catchment-wide | 0.8946 (0.0369) | 0.8946 (0.0369) | 0.8746 (0.0440) | 0.9460 (0.0248) |
| ELORN | Catchment-wide | 0.8870 (0.0335) | 0.8870 (0.0335) | 0.8843 (0.0377) | 0.9661 (0.0205) |
| BLAVET | Catchment-wide | 0.9048 (0.0318) | 0.9048 (0.0318) | 0.8903 (0.0402) | 0.9634 (0.0168) |
| ELLE | Catchment-wide | 0.8861 (0.0367) | 0.8861 (0.0367) | 0.8735 (0.0436) | 0.9641 (0.0178) |
| AULNE | Catchment-wide | 0.8673 (0.0362) | 0.8673 (0.0362) | 0.8549 (0.0373) | 0.9376 (0.0247) |
| SCORFF | Catchment-wide | 0.8810 (0.0353) | 0.8810 (0.0353) | 0.8811 (0.0334) | 0.9671 (0.0170) |
| CARES | COMBINED | 0.9313 (0.0312) | 0.9316 (0.0312) | 0.9278 (0.0326) | 0.9707 (0.0159) |
|  | Casaño | 0.8453 (0.0368) | 0.9053 (0.0333) |  | 0.9561 (0.0214) |
| SELLA | COMBINED | 0.8613 (0.0420) | 0.8629 (0.0422) | 0.9176 (0.0358) | 0.9555 (0.0194) |
|  | Piloña | 0.8954 (0.0387) | 0.9161 (0.0332) |  | 0.9569 (0.0196) |
| NARCEA | COMBINED | 0.8923 (0.0316) | 0.8923 (0.0316) | 0.8654 (0.0414) | 0.9666 (0.0159) |
| EO | Catchment-wide | 0.9449 (0.0224) | 0.9449 (0.0224) | 0.9319 (0.0239) | 0.9480 (0.0217) |
| ULLA | Catchment-wide | 0.9216 (0.0288) | 0.9216 (0.0288) | 0.9180 (0.0291) | 0.9688 (0.0187) |
| MOY | Trimoge | 0.9063 (0.0256) | 0.9063 (0.0256) | 0.8802 (0.0321) | 0.9271 (0.0233) |
| LAUNE | Cottoners | 0.8934 (0.0371) | 0.8934 (0.0371) | 0.8645 (0.0390) | 0.9135 (0.0333) |
| CORK | Awnaskirtaun | 0.8472 (0.0371) | 0.8811 (0.0341) |  | 0.8939 (0.0321) |
| BLACKWATER | Clydagh | 0.8681 (0.0431) | 0.8903 (0.0347) | 0.9120 (0.0310) | 0.9017 (0.0327) |
|  | Glen | 0.8451 (0.0436) | 0.8629 (0.0417) |  | 0.8721 (0.0415) |
| BARROW | Ballyclare Brg. | 0.9053 (0.0297) | 0.9053 (0.0297) | 0.8672 (0.0415) | 0.9236 (0.0269) |
| SUIR | Clodiagh | 0.8150 (0.0418) | 0.8206 (0.0413) | 0.8490 (0.0385) | 0.8530 (0.0375) |
|  | Beakstown | 0.7701 (0.0464) | 0.8129 (0.0388) |  | 0.8608 (0.0363) |
| BOYNE | Deel | 0.8945 (0.0308) | 0.9103 (0.0318) |  | 0.9205 (0.0305) |
|  | Moynalty | 0.8715 (0.0397) | 0.8859 (0.0361) | 0.9433 (0.0224) | 0.8985 (0.0349) |
|  | Skane Lwr | 0.8960 (0.0319) | 0.9068 (0.0323) |  | 0.9178 (0.0301) |
| **Mean** |  | **0.8614** | **0.8732** | **0.8974** | **0.9197** |

Table shows a) correct apportionment to individual sample sites within catchments, b) the sum of the apportionment to all samples in a catchment, c) the apportionment to catchment when all samples from within a catchment are pooled (i.e. the pool and allocate method), and d) the sum of apportionment to all samples in a reporting region. In the case of the individual sample simulations a 108 sample baseline was used. One hundred fish were used in the mixture sample with 100 re-samplings of the mixture sample and baseline samples. Individual samples are identified by tributary name; see Additional File 1 for details of latitude and longitude, collection date and individual sample size.
