# Supplementary material for: Genetic stock identification of Atlantic salmon (Salmo salar) populations in the southern part of the European range
Source: BMC Genet. 2010 Apr 29;11:31. doi: 10.1186/1471-2156-11-31 (PMC2882343; doi:10.1186/1471-2156-11-31)
Supplement: Additional file 3 — Multi-sample simulations in ONCOR using unequal proportions of baseline samples. Four simulated mixtures are presented; each mixture comprised eight samples with a combination of unequal proportions making up each individual mixture, with mixtures 1 and 2 including a river from each reporting region, mixture 3 a combination of southern rivers and mixture 4 a combination of northern rivers. Stock compositions were estimated at the level of individual sample site and reporting region via the allocate and sum method. One-hundred fish were used in the mixture sample, with 100 simulations. [file 1471-2156-11-31-S3.DOC]

**Additional File 3. Multi-sample simulations in ONCOR using unequal proportions of baseline samples.**

| Baseline sample | Proportion | Sample estimate  (SD) | Regional estimate  (SD) |
| --- | --- | --- | --- |
| **Mixture 1** | | | |
| CARES (2002) | 0.10 | 0.0712 (0.0202) | 0.1035 (0.0107) |
| CORK BLACKWATER (Clydagh) | 0.15 | 0.0550 (0.0259) | 0.1148 (0.0336) |
| ELORN | 0.20 | 0.1212 (0.0251) | 0.2012 (0.0133) |
| FOWEY (Treverbyn) | 0.20 | 0.0945 (0.0293) | 0.1875 (0.0292) |
| GRUINARD (Ghiubhsachain) | 0.10 | 0.0468 (0.0161) | 0.0898 (0.0240) |
| LOCH LOMOND (Fruin) | 0.15 | 0.1392 (0.0122) | 0.1710 (0.0208) |
| NITH (River Cairn) | 0.05 | 0.0118 (0.0131) | 0.0834 (0.0264) |
| TEST | 0.05 | 0.0461 (0.0066) | 0.0490 (0.0033) |
| **Mixture 2** | | | |
| AVON (Bugmoor Hatches) | 0.10 | 0.0638 (0.0171) | 0.0977 (0.0047) |
| CORK BLACKWATER (Awnaskirtaun) | 0.10 | 0.0284 (0.0191) | 0.0889 (0.0277) |
| DOON (Ness Glen) | 0.10 | 0.0496 (0.0188) | 0.0822 (0.0236) |
| EO | 0.05 | 0.0466 (0.0058) | 0.0498 (0.0059) |
| LOCH LOCHY (Lundy Tributary) | 0.10 | 0.0466 (0.0058) | 0.0821 (0.0225) |
| EDEN (Swindale Beck) | 0.10 | 0.0499 (0.0183) | 0.1344 (0.0274) |
| SEE | 0.30 | 0.1957 (0.0335) | 0.2840 (0.0161) |
| WYE | 0.15 | 0.0660 (0.0233) | 0.1811 (0.0240) |
| **Mixture 3** | | | |
| CARES (Casano) | 0.20 | 0.1757 (0.0151) | 0.2973 (0.0075) |
| NARCEA (2002) | 0.10 | 0.0592 (0.0188) |
| SCORFF | 0.15 | 0.0717 (0.0249) | 0.2391 (0.0157) |
| SELUNE | 0.10 | 0.0601 (0.0198) |
| AVON (Avon Bridge) | 0.15 | 0.1185 (0.0201) | 0.1994 (0.0028) |
| ITCHEN | 0.05 | 0.0587 (0.0137) |
| DEE (Abbey Brook) | 0.10 | 0.0436 (0.0175) | 0.2045 (0.0233) |
| EXE (Fernyball, Sherdon Water) | 0.15 | 0.0668 (0.0229) |
| **Mixture 4** | | | |
| SUIR (Beakstown) | 0.10 | 0.0179 (0.0149) | 0.2054 (0.0427) |
| CORK BLACKWATER (Clydagh) | 0.10 | 0.0393 (0.0249) |
| ESK (Boyken Burn) | 0.15 | 0.0364 (0.0190) | 0.3418 (0.0444) |
| RIBBLE (Hammerton Hall, River Hodder) | 0.20 | 0.1043 (0.0275) |
| BOYNE (Skane Lwr) | 0.15 | 0.0880 (0.0244) | 0.1957 (0.0350) |
| AYR (Lugar Water) | 0.05 | 0.0470 (0.0171) |
| EWE (Talladale, Grudie Bay) | 0.10 | 0.0364 (0.0190) | 0.1972 (0.0395) |
| LAUNE (Cottoners) | 0.15 | 0.0842 (0.0274) |

Four simulated mixtures are presented; each mixture comprised eight samples with a combination of unequal proportions making up each individual mixture, with mixtures 1 and 2 including a river from each reporting region, mixture 3 a combination of southern rivers (incorporating two rivers from each reporting region) and mixture 4 a combination of northern rivers (incorporating two rivers from each reporting region). Stock compositions were estimated at the level of individual sample site (3rd column, ‘Sample Estimate’) and reporting region (4^th^ column, ‘Regional Estimate’) via the allocate and sum method. One-hundred fish were used in the mixture sample, with 100 simulations. Individual samples are identified by tributary name; see Additional File 1 for details of latitude and longitude, collection date and individual sample size.
