# Supplementary material for: Genetic stock identification of Atlantic salmon (Salmo salar) populations in the southern part of the European range
Source: BMC Genet. 2010 Apr 29;11:31. doi: 10.1186/1471-2156-11-31 (PMC2882343; doi:10.1186/1471-2156-11-31)
Supplement: Additional file 4 — Summary of population genetic statistics. Summary statisitcs for all popualtions, including: sample size, number of alleles, observed and expected heterozygosities, and probability of conformance to Hardy-Weinberg equilibrium. [file 1471-2156-11-31-S4.DOC]

**Additional File 4. Genetic summary statistics.** Number of observations (N), number of alleles, observed (H_O_) and expected (H_E_) heterozygosities and probability of conformance to Hardy-Weinberg equilibrium (HWE; where temporal samples were tested separately the lowest p-value from the individual tests is presented for each locus).

| **Catchment**  **Sample Site** |  | **2201** | **85** | **171** | **157** | **144** | **289** | **417** | **G7** | **197** | **202** | **1605** | **2210** | **Mean** |
| --- | --- | --- | --- | --- | --- | --- | --- | --- | --- | --- | --- | --- | --- | --- |
| **Scotland** |  |  |  |  |  |  |  |  |  |  |  |  |  |  |
| BLACKWATER  (1)  ROAG.Tar  Blackwater, Tarbert Burn | N  No. alleles  H_E_  H_O_  HWE | 33  20  0.9129  0.8485  0.0021 | 33  12  0.7860  0.8182  0.7967 | 33  12  0.8403  0.8485  0.8710 | 33  19  0.9085  0.9697  0.7366 | 33  20  0.9120  1.0000  0.0907 | 33  4  0.6194  0.6666  0.6616 | 33  15  0.7975  0.7575  0.1893 | 33  15  0.8669  0.7575  0.0156 | 33  16  0.8826  0.8788  0.4279 | 33  10  0.8132  0.8788  0.9219 | 33  8  0.8034  0.8788  0.3138 | 33  12  0.8278  0.7879  0.0693 | 33  13.5833  0.8309  0.8409 |
| CREED  (2)  CREED.All  Allt Ruadh | N  No. alleles  H_E_  H_O_  HWE | 36  18  0.9109  1.0000  0.5300 | 36  10  0.8182  0.8611  0.7639 | 36  12  0.8444  0.8333  0.1913 | 36  17  0.8921  0.9166  0.3050 | 36  24  0.9157  0.8888  0.2994 | 36  4  0.6629  0.7222  0.6204 | 36  13  0.8433  0.8055  0.2604 | 36  15  0.8335  0.7500  0.0099 | 36  21  0.9104  0.8611  0.0222 | 35  9  0.8510  0.7142  0.0026 | 36  8  0.7469  0.7778  0.7129 | 36  11  0.7717  0.8333  0.9041 | 35.9166  13.5000  0.8334  0.8303 |
| LAXFORD  (3)  LAX.Ach  Laxford (Achfary) | N  No. alleles  H_E_  H_O_  HWE | 32  21  0.9236  0.9688  0.2667 | 32  11  0.8257  0.9063  0.0750 | 32  11  0.7665  0.7813  0.4975 | 32  19  0.8981  1.0000  0.7531 | 32  15  0.8942  0.9687  0.0153 | 32  4  0.5777  0.7187  0.6274 | 32  13  0.8459  0.9062  0.6366 | 32  11  0.7887  0.8437  0.9898 | 32  15  0.8493  0.9063  0.3933 | 32  10  0.7996  0.7813  0.0259 | 32  7  0.8186  0.9063  0.4183 | 32  9  0.8109  0.7813  0.1534 | 32  12.1666  0.8165  0.8723 |
| LAXFORD  (3)  LAX.Mai  Laxford | N  No. alleles  H_E_  H_O_  HWE | 30  21  0.9105  0.9333  0.8688 | 30  13  0.8449  0.7333  0.0053 | 30  10  0.8306  0.9667  0.3336 | 30  21  0.9074  1.0000  0.3461 | 30  21  0.9218  0.9666  0.9747 | 30  4  0.6897  0.7000  0.9636 | 30  16  0.8332  0.9000  0.2767 | 30  14  0.8589  0.8666  0.3788 | 29  16  0.8876  0.6207  0.0003 | 30  9  0.8558  0.9333  0.1937 | 30  7  0.8085  0.8000  0.0952 | 30  15  0.8224  0.8667  0.3958 | 29.9166  13.9166  0.8476  0.8572 |
| LAXFORD  (3)  LAX.Thu  Thull Tributary | N  No. alleles  H_E_  H_O_  HWE | 32  9  0.7613  0.7500  0.1588 | 32  8  0.7798  0.8750  0.1413 | 32  7  0.7712  0.8438  0.0655 | 32  8  0.8035  0.9688  0.2089 | 32  7  0.7916  0.9062  0.0025 | 32  3  0.5181  0.5937  0.8568 | 32  8  0.5635  0.6250  0.2064 | 32  11  0.8563  0.8750  0.0046 | 31  11  0.7479  0.7742  0.0055 | 32  7  0.7803  0.9063  0.0007 | 31  6  0.7887  0.8065  0.1806 | 31  5  0.4638  0.4516  0.1145 | 31.7500  7.5000  0.7188  0.7813 |
| GRUINARD  (4)  GRU.Abh  Abhainn Glen na Muice | N  No. alleles  H_E_  H_O_  HWE | 27  22  0.9051  0.8889  0.2636 | 27  11  0.8549  0.8519  0.0082 | 27  10  0.8272  0.9259  0.4149 | 27  21  0.9065  0.8889  0.2721 | 27  24  0.9176  0.8888  0.2323 | 27  3  0.6256  0.6666  0.8741 | 27  9  0.7861  0.7407  0.2819 | 27  12  0.8363  0.7777  0.6503 | 27  13  0.8788  1.0000  0.9821 | 27  10  0.8378  0.8889  0.6922 | 27  8  0.8008  0.8519  0.0016 | 27  12  0.8220  0.9259  0.9253 | 27  12.9166  0.8332  0.8580 |
| GRUINARD  (4)  GRU.Ghi  Ghiubhsachain Burn | N  No. alleles  H_E_  H_O_  HWE | 27  17  0.8880  0.9259  0.2958 | 27  11  0.8001  0.7407  0.2321 | 27  12  0.8008  0.8148  0.2476 | 27  20  0.8907  1.0000  0.1672 | 27  19  0.8813  0.8518  0.0585 | 27  3  0.5669  0.7037  0.2196 | 27  13  0.8462  0.7777  0.0121 | 27  13  0.8601  0.7777  0.0003 | 27  13  0.8682  0.8889  0.2105 | 27  10  0.8305  0.8519  0.8025 | 27  7  0.7407  0.8148  0.1563 | 27  8  0.7690  0.7778  0.6431 | 27  12.1666  0.8118  0.8271 |
| EWE  (5)  EWE.Gru  Talladale (Grudie bay, small tributaries at Loch Maree shore) | N  No. alleles  H_E_  H_O_  HWE | 26  15  0.8758  0.8462  0.1067 | 26  11  0.8160  0.7308  0.3654 | 26  10  0.8203  0.7692  0.5558 | 26  18  0.8580  0.8462  0.3505 | 26  16  0.8679  0.8461  0.1704 | 26  4  0.6418  0.8076  0.5643 | 26  13  0.7989  0.8461  0.6593 | 26  12  0.8096  0.8076  0.4394 | 26  12  0.8558  0.7308  0.0801 | 26  8  0.8417  0.9615  0.2707 | 26  7  0.7314  0.7692  0.9458 | 26  11  0.7996  0.7308  0.2627 | 26  11.4166  0.8097  0.8076 |
| EWE  (5)  EWE.Kem  Kemsary, (D/S Loch Maree) | N  No. alleles  H_E_  H_O_  HWE | 23  12  0.8431  1.0000  0.7238 | 23  9  0.8359  1.0000  0.7905 | 23  11  0.7933  0.8261  0.8838 | 23  14  0.8593  0.9130  0.1920 | 23  16  0.8919  1.0000  0.5583 | 23  5  0.6177  0.6086  0.4687 | 23  12  0.7841  0.7826  0.6538 | 23  11  0.8095  0.8695  0.9086 | 23  14  0.8557  0.9130  0.0694 | 23  8  0.8285  0.8261  0.2485 | 23  7  0.7961  0.9565  0.1464 | 23  10  0.7436  0.8261  0.2177 | 23  10.7500  0.8049  0.8768 |
| LOCH LOCHY  (6)  LOC.loc  Lochy (Below Loch Lochy) | N  No. alleles  H_E_  H_O_  HWE | 46  23  0.9231  0.9348  0.4341 | 46  15  0.8561  0.9348  0.6792 | 46  11  0.8073  0.8696  0.7136 | 46  22  0.9160  0.9348  0.3324 | 46  24  0.9312  0.9565  0.6818 | 46  4  0.6334  0.7173  0.6761 | 46  16  0.8549  0.8695  0.1161 | 46  14  0.8764  0.8913  0.6992 | 46  18  0.9067  0.8696  0.0026 | 46  10  0.8713  0.8696  0.4454 | 45  10  0.7985  0.8222  0.0851 | 46  13  0.8466  0.8478  0.0573 | 45.9166  15  0.8518  0.8764 |
| LOCH LOCHY  (6)  LOC.lun  Lundy Tributary | N  No. alleles  H_E_  H_O_  HWE (p-value) | 26  16  0.9050  0.9231  0.2717 | 26  12  0.7926  0.8462  0.1089 | 26  12  0.8011  0.7692  0.3058 | 26  16  0.8801  0.9231  0.5866 | 26  18  0.9099  0.9230  0.0093 | 26  3  0.6076  0.6538  0.8588 | 26  13  0.8381  0.9230  0.0247 | 26  12  0.8345  0.9230  0.1060 | 26  15  0.8758  0.8846  0.2958 | 26  10  0.8332  0.9615  0.5066 | 26  7  0.7756  0.8846  0.8405 | 26  7  0.7627  0.7692  0.1392 | 26  11.7500  0.8180  0.8653 |
| AWE  (7)  AWE.Cla  Clachan Dubh Tributary | N  No. alleles  H_E_  H_O_  HWE | 35  15  0.8808  0.9714  0.7878 | 35  11  0.7709  0.7714  0.0463 | 35  10  0.8348  0.8857  0.8948 | 35  17  0.8748  0.9142  0.8539 | 35  16  0.8887  0.9428  0.7293 | 35  3  0.5556  0.6285  0.7762 | 35  13  0.7749  0.8285  0.4460 | 35  12  0.8442  0.7714  0.0663 | 35  11  0.8661  0.9428  0.5951 | 35  11  0.8236  0.8285  0.0429 | 35  7  0.8042  0.8857  0.5866 | 35  10  0.7507  0.82857  0.3892 | 35  11.3333  0.8058  0.8500 |
| AWE  (7)  AWE.Mai  Awe | N  No. alleles  H_E_  H_O_  HWE | 35  14  0.8423  0.8857  0.1245 | 35  8  0.7895  0.8000  0.0546 | 35  11  0.8284  0.8571  0.6344 | 35  17  0.8923  0.8857  0.1400 | 35  14  0.8828  0.9714  0.5351 | 35  4  0.4906  0.5714  0.0285 | 35  15  0.8712  0.8857  0.4645 | 35  11  0.7594  0.6857  0.0551 | 35  16  0.8768  0.8571  0.3452 | 35  9  0.8245  0.9142  0.6790 | 35  8  0.8106  0.8571  0.3907 | 35  8  0.7852  0.7428  0.1071 | 35  11.2500  0.8045  0.8261 |
| AWE  (7)  AWE.Bra  Braevallich Tributary | N  No. alleles  H_E_  H_O_  HWE | 35  14  0.8764  0.9428  0.6113 | 35  10  0.8368  0.9714  0.7884 | 35  12  0.8340  0.8571  0.0116 | 35  14  0.8641  0.9142  0.3583 | 35  17  0.9046  1.0000  0.5378 | 35  5  0.6892  0.6285  0.5816 | 35  13  0.7535  0.8571  0.2207 | 35  12  0.8352  0.8857  0.4299 | 35  16  0.8082  0.7714  0.1646 | 35  11  0.8407  0.9428  0.0952 | 35  8  0.8296  0.8571  0.9654 | 35  7  0.7514  0.6571  0.0145 | 35  11.5833  0.8186  0.8571 |
| LOCH LOMOND  (8)  LOM.End  Endrick | N  No. alleles  H_E_  H_O_  HWE | 25  17  0.8675  1.0000  0.0117 | 25  7  0.7446  0.8400  0.0725 | 25  7  0.7345  0.7200  0.1458 | 25  12  0.8552  1.0000  0.1707 | 25  15  0.8705  1.0000  0.0653 | 25  5  0.6683  0.6000  0.0640 | 25  9  0.7752  0.8000  0.0109 | 25  11  0.7245  0.7600  0.0057 | 25  10  0.8382  0.8800  0.0114 | 25  11  0.8051  0.6800  0.0060 | 25  7  0.7362  0.9600  0.0582 | 25  7  0.6569  0.7200  0.5108 | 25  9.8333  0.7730  0.8300 |
| LOCH LOMOND  (8)  LOM.Fru  Fruin | N  No. alleles  H_E_  H_O_  HWE | 51  19  0.9147  0.9608  0.0168 | 51  8  0.6659  0.7059  .5263 | 51  9  0.8169  0.6863  0.0077 | 51  15  0.8878  0.9608  0.2806 | 51  19  0.8947  0.9607  0.1227 | 51  4  0.6267  0.7058  0.2387 | 51  11  0.8138  0.8431  0.3640 | 51  17  0.8743  0.8039  0.0679 | 51  14  0.8540  0.9020  0.0912 | 51  10  0.8454  0.9804  0.0110 | 51  8  0.7294  0.6275  0.0031 | 51  10  0.6859  0.7059  0.3219 | 51  12  0.8008  0.8202 |
| CLYDE  (9)  CLY.Boc  River Kelvin (Allander Water, Boclair Bridge) | N  No. alleles  H_E_  H_O_  HWE | 30  17  0.8998  0.9666  0.5876 | 30  14  0.8208  0.8333  0.5864 | 30  14  0.8203  0.8666  0.6277 | 30  17  0.8933  0.9666  0.6767 | 30  19  0.8982  0.9666  0.7516 | 30  4  0.6408  0.6666  0.7912 | 30  13  0.8627  1.0000  0.1458 | 30  13  0.8266  0.7333  0.1035 | 30  17  0.8856  0.6333  0.0000 | 30  9  0.8433  0.8333  0.0296 | 30  9  0.8116  0.8000  0.9330 | 30  9  0.6570  0.7666  0.1071 | 30  12.9166  0.8217  0.8361 |
| CLYDE  (9)  CLY.Cal  River Cart (River Calder, Loch winnoch) | N  No. alleles  H_E_  H_O_  HWE | 25  16  0.8920  1.0000  0.0785 | 25  9  0.8275  0.9600  0.7474 | 25  12  0.8358  0.7200  0.0172 | 25  15  0.87589  0.9200  0.2111 | 25  13  0.8874  1.0000  0.8206 | 25  4  0.6676  0.6400  0.6168 | 25  10  0.8106  0.8800  0.7769 | 25  12  0.8281  0.8000  0.1458 | 25  17  0.9010  0.6800  0.0000 | 25  10  0.8220  0.8000  0.0044 | 25  7  0.7659  0.7600  0.0647 | 25  9  0.7651  0.7200  0.1548 | 25  11.1666  0.8232  0.8233 |
| AYR  (10)  AYR.Gle  Glenmuir Water (Dalblair) | N  No. alleles  H_E_  H_O_  HWE | 30  13  0.8761  0.9333  0.9092 | 30  11  0.8101  0.9000  0.9515 | 30  10  0.8262  0.8666  0.2613 | 30  16  0.8412  0.8000  0.1149 | 30  16  0.8535  0.8666  0.4668 | 30  4  0.6843  0.5666  0.0316 | 30  13  0.7864  0.7333  0.1338 | 30  14  0.8454  0.7666  0.0195 | 30  12  0.8165  0.9333  0.9495 | 30  10  0.8460  0.8000  0.0501 | 30  7  0.6473  0.7000  0.8396 | 30  9  0.7741  0.9666  0.1935 | 30  11.2500  0.8006  0.8194 |
| AYR  (10)  AYR.How  Ayr (Howford Bridge) | N  No. alleles  H_E_  H_O_  HWE | 30  17  0.8858  0.9333  0.3231 | 30  12  0.7928  0.8000  0.9252 | 30  13  0.8557  0.8000  0.1526 | 30  18  0.9148  0.9333  0.7993 | 30  16  0.8954  0.7333  0.0967 | 30  6  0.6784  0.7000  0.0343 | 30  12  0.8423  0.8666  0.2319 | 30  15  0.8697  0.9333  0.5270 | 30  14  0.8266  0.7666  0.0000 | 30  10  0.8579  0.9000  0.2178 | 30  7  0.7757  0.8666  0.9782 | 30  9  0.7069  0.8000  0.4897 | 30  12.4166  0.8252  0.8361 |
| AYR  (10)  AYR.Lug  Lugar Water (Near council) | N  No. alleles  H_E_  H_O_  HWE | 68  24  0.9196  0.9411  0.3589 | 68  11  0.8297  0.8235  0.0391 | 68  14  0.8520  0.8529  0.4957 | 68  20  0.8922  0.8970  0.1933 | 68  23  0.9009  0.9264  0.5689 | 68  4  0.6560  0.7352  0.8877 | 68  13  0.8589  0.8676  0.1428 | 68  15  0.8731  0.8088  0.4662 | 68  18  0.8831  0.7941  0.0000 | 68  12  0.8749  0.7794  0.0018 | 68  9  0.7556  0.7058  0.0038 | 68  12  0.8001  0.7941  0.1283 | 68  14.5833  0.8413  0.8272 |
| DOON  (11)  DOON.Muc  Muck Water | N  No. alleles  H_E_  H_O_  HWE | 30  19  0.9208  0.9667  0.8857 | 30  13  0.8773  0.9667  0.7791 | 30  14  0.8101  0.9000  0.2821 | 30  17  0.9025  0.9667  0.0478 | 30  20  0.8944  0.9666  0.7008 | 30  4  0.6511  0.7333  0.2540 | 30  10  0.8568  0.8666  0.6975 | 30  18  0.9002  0.7666  0.0045 | 30  18  0.8971  0.9667  0.5740 | 30  11  0.8584  0.9000  0.6464 | 30  9  0.8310  0.8333  0.4401 | 30  9  0.8133  0.8667  0.0243 | 30  13.5000  0.8510  0.8916 |
| DOON  (11)  DOON.Nes  Ness Glen | N  No. alleles  H_E_  H_O_  HWE | 27  17  0.9098  0.9630  0.0055 | 27  10  0.7665  0.9259  0.4845 | 27  12  0.8418  0.9259  0.0693 | 27  15  0.8630  1.0000  0.1472 | 27  16  0.8833  0.9259  0.0324 | 27  5  0.6760  0.8518  0.0877 | 27  14  0.8760  0.8888  0.2160 | 27  15  0.8754  0.9259  0.0648 | 27  16  0.8899  0.8889  0.0366 | 26  10  0.8260  0.8462  0.3455 | 27  9  0.7651  0.7778  0.4847 | 27  9  0.7942  0.8519  0.1821 | 26.9166  12.3333  0.8305  0.8976 |
| DOON  (11)  DOON.Ske  Skeldon Mills | N  No. alleles  H_E_  H_O_  HWE | 29  22  0.9153  0.9655  0.8978 | 29  11  0.8216  0.7241  0.0887 | 29  12  0.8205  0.8621  0.6774 | 29  20  0.9182  1.0000  1.0000 | 29  24  0.9302  0.9655  0.6472 | 29  5  0.6729  0.6551  0.6576 | 29  15  0.8693  0.8965  0.7528 | 29  16  0.8997  0.9310  0.8491 | 29  15  0.8705  0.8276  0.2832 | 29  9  0.8458  0.9310  0.8102 | 29  9  0.7901  0.7241  0.2905 | 29  9  0.8033  0.7931  0.1809 | 29  13.9166  0.8464  0.8563 |
| CREE  (12)  CREE.Whi  Whitehills | N  No. alleles  H_E_  H_O_  HWE | 39  21  0.9287  0.9487  0.3493 | 39  11  0.8303  0.8205  0.2979 | 39  18  0.9024  0.8974  0.4242 | 39  20  0.8982  0.9230  0.2674 | 39  21  0.9069  0.9230  0.0450 | 39  5  0.6785  0.7435  0.9100 | 39  17  0.8841  0.8461  0.0337 | 39  14  0.8322  0.8461  0.9595 | 38  14 0.8605  0.7105  0.0442 | 38  10  0.8420  0.8947  0.8647 | 39  8  0.7755  0.7948  0.2424 | 39  10  0.7598  0.7948  0.2189 | 38.8333  14.0833  0.8416  0.8453 |
| FLEET  (13)  FLE.Big  Big Water of Fleet | N  No. alleles  H_E_  H_O_  HWE | 21  18  0.8914  1.0000  0.5714 | 21  12  0.7845  0.9048  0.8325 | 21  12  0.8104  0.9048  0.7836 | 21  16  0.9022  1.0000  0.4065 | 21  18  0.8827  0.9523  0.7383 | 21  4  0.6114  0.5238  0.5153 | 21  10  0.8361  0.8095  0.4230 | 21  11  0.8687  0.9523  0.5829 | 21  10  0.8326  0.4286  0.0000 | 20  10  0.8295  0.9000  0.5879 | 21  6  0.7639  0.8571  0.1259 | 21  10  0.7291  0.6190  0.0239 | 20.9166  11.4166  0.8119  0.8210 |
| FLEET  (13)  FLE.Lit  Little Water of Fleet | N  No. alleles  H_E_  H_O_  HWE | 26  12  0.8644  0.8846  0.0357 | 26  10  0.8261  0.9615  0.4220 | 26  11  0.7911  0.7692  0.1271 | 26  16  0.8993  1.0000  0.1333 | 26  12  0.8652  1.0000  0.1974 | 26  4  0.6169  0.7692  0.1619 | 26  10  0.8154  0.9230  0.3497 | 26  14  0.8458  0.8076  0.1296 | 26  14  0.8608  0.8077  0.1402 | 25  8  0.8267  0.8400  0.3114 | 26  8  0.8040  0.8462  0.3849 | 26  11  0.7918  0.7308  0.0287 | 25.9166  10.8333  0.8173  0.8616 |
| NITH  (14)  NITH.RC  Nith, River Cairn | N  No. alleles  H_E_  H_O_  HWE | 30  17  0.9062  0.9333  0.2116 | 30  7  0.7827  0.7667  0.6165 | 30  13  0.8660  0.9333  0.3225 | 30  20  0.9132  0.9667  0.6332 | 30  21  0.9078  0.8666  0.1099 | 30  4  0.6171  0.5333  0.0787 | 30  13  0.8482  0.9666  0.7567 | 30  17  0.8916  0.8333  0.2723 | 30  18  0.8896  0.9667  0.2863 | 30  12  0.8686  0.9000  0.1472 | 30  8  0.7800  0.8000  0.1382 | 30  12  0.8187  0.9000  0.3661 | 30  13.5000  0.8408  0.8638 |
| NITH  (14)  NITH.NM  Nith, main, Buccleuch Estate | N  No. alleles  H_E_  H_O_  HWE | 30  25  0.9251  1.0000  0.9674 | 30  11  0.8074  0.8000  0.5375 | 30  13  0.8187  0.8333  0.6770 | 30  16  0.9068  0.9667  0.1954 | 30  19  0.9013  0.8666  0.1754 | 30  4  0.6108  0.6666  0.1740 | 30  13  0.8616  0.8333  0.7961 | 30  15  0.8632  0.8666  0.7589 | 30  17  0.8654  0.9333  0.8090 | 30  10  0.8315  0.7667  0.2842 | 30  6  0.7725  0.8000  0.8569 | 30  12  0.7833  0.8667  0.8799 | 30  13.4166  0.8289  0.8500 |
| NITH  (14)  NITH.NSC  Nith, Scaur Water | N  No. alleles  H_E_  H_O_  HWE | 30  18  0.9020  1.0000  0.0176 | 30  9  0.7655  0.8333  0.2972 | 30  9  0.7569  0.7333  0.4487 | 30  15  0.9036  0.9667  0.3104 | 30  17  0.8713  0.9000  0.4099 | 30  4  0.5817  0.5666  1.0000 | 30  12  0.8299  0.9000  0.1840 | 30  11  0.8219  0.8666  0.2225 | 30  14  0.8847  0.8667  0.2479 | 30  11  0.8143  0.7000  0.0312 | 30  7  0.8106  0.8667  0.3329 | 30  9  0.6725  0.6000  0.0305 | 30  11.3333  0.8012  0.8166 |
| ANNAN  (15)  ANN.Bk  Birnock Water | N  No. alleles  H_E_  H_O_  HWE | 29  19  0.8832  0.9655  0.9037 | 29  10  0.7177  0.6552  0.0613 | 29  11  0.7838  0.8621  0.7944 | 29  17  0.8912  0.9655  0.7839 | 29  17  0.8957  0.9310  0.3865 | 29  4  0.6368  0.7241  0.9413 | 29  15  0.8589  0.8620  0.0667 | 29  12.8333  0.8105  0.8183  0.1749 | 29  15  0.8428  0.5517  0.0011 | 28  11  0.8341  0.7857  0.5122 | 29  7  0.7551  0.7931  0.4396 | 29  9  0.7505  0.8276  0.0309 | 28.9166  12.8333  0.8105  0.8183 |
| ANNAN  (15)  ANN.Ev  Evan Water | N  No. alleles  H_E_  H_O_  HWE | 35  19  0.9153  0.9428  0.3570 | 35  12  0.8427  0.8857  0.6014 | 35  16  0.8844  1.0000  0.8866 | 35  23  0.9086  1.0000  0.5123 | 35  19  0.9053  0.9142  0.5213 | 35  4  0.5889  0.7428  0.2841 | 34  16  0.8611  0.8529  0.0335 | 35  15  0.8700  0.7714  0.0064 | 34  16  0.8594  0.6176  0.0007 | 34  12  0.8582  0.9117  0.7380 | 34  9  0.8066  0.8235  0.6655 | 34  12  0.8108  0.8235  0.4334 | 34.5833  14.4166  0.8426  0.8572 |
| ANNAN  (15)  ANN.Whi  Wamphray | N  No. alleles  H_E_  H_O_  HWE | 31  19  0.9065  0.9677  0.8234 | 31  10  0.7877  0.8387  0.8403 | 31  16  0.8450  0.7741  0.0100 | 31  16  0.8768  0.9032  0.3077 | 31  22  0.9226  0.9677  0.6718 | 31  5  0.6587  0.6774  0.2941 | 31  14  0.8526  0.9032  0.8415 | 31  17  0.8787  0.7741  0.0037 | 31  13  0.8772  0.7096  0.0071 | 31  12  0.8310  0.8387  0.3254 | 31  6  0.7826  0.7741  0.1805 | 31  11  0.7906  0.7741  0.3715 | 31  13.4166  0.8342  0.8252 |
| **England & Wales** |  |  |  |  |  |  |  |  |  |  |  |  |  |  |
| ESK (Border)  (16)  ESKB.Lid  Liddel Water | N  No. alleles  H_E_  H_O_  HWE | 35  16  0.8923  0.9714  0.1110 | 35  10  0.8162  0.8286  0.1355 | 35  15  0.8523  0.9714  0.7906 | 35  22  0.9197  0.9143  0.0010 | 35  18  0.8986  1.0000  0.0175 | 35  4  0.6821  0.7714  0.3009 | 35  14  0.8772  0.9714  0.1755 | 34  14  0.8674  0.8529  0.1191 | 35  14  0.8681  0.8571  0.5064 | 35  10  0.8439  0.8286  0.1157 | 35  8  0.7769  0.8286  0.1357 | 35  9  0.7987  0.7429  0.0302 | 34.9166  12.8333  0.8411  0.8782 |
| ESK (Border)  (16)  ESKB.Boy  Boyken Burn | N  No. alleles  H_E_  H_O_  HWE | 30  19  0.9159  0.9667  0.5671 | 30  12  0.8520  0.9333  0.0024 | 30  13  0.8337  0.9000  0.3761 | 30  16  0.8961  0.9667  0.9319 | 30  20  0.8782  0.8666  0.2319 | 30  5  0.6161  0.5333  0.2252 | 30  16  0.86756  0.9000  0.5232 | 30  18  0.8858  0.9000  0.5144 | 30  17  0.8997  0.7667  0.0071 | 30  9  0.8214  0.8667  0.4081 | 30  9  0.8063  0.8333  0.5290 | 30  11  0.8010  0.8667  0.4476 | 30  13.7500  0.8394  0.8583 |
| ESK (Border)  (16)  ESKB.Ewe  Ewes Water | N  No. alleles  H_E_  H_O_  HWE | 30  24  0.9207  0.9333  0.3546 | 30  8  0.7790  0.8667  0.6773 | 30  15  0.8601  0.9667  0.9594 | 30  21  0.9127  1.0000  0.6356 | 30  17  0.9008  0.9666  0.1033 | 30  5  0.6489  0.7666  0.9171 | 30  15  0.8670  0.9666  0.9319 | 30  18  0.8670  0.9000  0.9717 | 30  19  0.8934  0.9667  0.8580 | 30  12  0.8299  0.8333  0.5803 | 30  9  0.8058  0.8333  0.5479 | 30  12  0.7445  0.7667  0.0350 | 30  14.5833  0.8358  0.8972 |
| EDEN  (17)  EDEN.Dac  Darce Beck | N  No. alleles  H_E_  H_O_  HWE | 30  19  0.8966  0.9333  0.1416 | 30  9  0.6581  0.7000  0.6991 | 30  14  0.8660  0.9000  0.6010 | 30  15  0.8907  0.9667  0.6534 | 30  16  0.7714  0.8666  0.2910 | 30  4  0.6451  0.6000  0.0259 | 30  13  0.8578  0.8666  0.2011 | 31  15  0.8824  0.9677  0.5202 | 29  15  0.8463  0.7931  0.1981 | 30  11  0.8240  0.8000  0.2020 | 30  6  0.7627  0.6667  0.3442 | 31  10  0.8078  0.8065  0.3662 | 30.0833  12.2500  0.8090  0.8222 |
| EDEN  (17)  EDEN.Sca  Scandal Beck | N  No. alleles  H_E_  H_O_  HWE | 31  17  0.9030  0.9032  0.0666 | 31  9  0.7887  0.7097  0.0056 | 31  14  0.8400  0.8065  0.1329 | 31  15  0.8924  0.9032  0.6209 | 31  19  0.9065  0.9677  0.8307 | 31  4  0.6577  0.6774  0.7438 | 31  12  0.8354  0.7741  0.0145 | 20  17  0.8805  0.9500  0.6935 | 31  20  0.8904  0.9355  0.1398 | 25  10  0.8459  0.8800  0.7970 | 29  7  0.8039  0.8966  0.9220 | 29  11  0.8194  0.7931  0.1196 | 29.2500  12.9166  0.8386  0.8497 |
| EDEN  (17)  EDEN.Swin  Swindale Beck | N  No. alleles  H_E_  H_O_  HWE | 30  18  0.9079  0.9667  0.9119 | 30  9  0.7193  0.6667  0.4673 | 30  14  0.8526  0.9333  0.4806 | 30  16  0.8944  0.9333  0.0583 | 30  21  0.8686  0.9000  0.4814 | 30  4  0.6919  0.7666  0.8347 | 30  12  0.8358  0.9000  0.8279 | 28  14  0.8870  0.9285  0.6636 | 29  13  0.8234  0.8276  0.1960 | 30  12  0.8476  0.8000  0.1325 | 29  8  0.7964  0.7241  0.2477 | 30  11  0.8331  0.7667  0.2436 | 29.6666  12.6666  0.8298  0.8428 |
| DERWENT  (18)  DERW.Dash  Dash Beck | N  No. alleles  H_E_  H_O_  HWE | 25  19  0.9166  1.0000  0.1939 | 25  13  0.8191  0.8800  0.3568 | 25  12  0.8536  0.8400  0.4653 | 25  18  0.8897  0.9600  0.3082 | 25  23  0.9211  0.9200  0.1132 | 25  4  0.6692  0.7200  0.7679 | 25  16  0.8397  0.8800  0.9729 | 25  13  0.8597  0.8400  0.0331 | 25  18  0.9042  0.8000  0.0015 | 25  10  0.8328  0.8000  0.0644 | 25  9  0.7821  0.8000  0.0783 | 25  9  0.6362  0.6800  0.6520 | 25  13.6666  0.8270  0.8433 |
| DERWENT  (18)  DERW.Mar  Marron | N  No. alleles  H_E_  H_O_  HWE | 30  20  0.9180  0.9000  0.4740 | 30  9  0.7794  0.7667  0.0943 | 30  15  0.8708  0.8333  0.0199 | 30  21  0.9175  0.8667  0.1025 | 30  23  0.9202  0.9666  0.6699 | 30  5  0.63822  0.7333  0.6809 | 30  15  0.8375  0.9666  0.4817 | 30  14  0.8696  0.8000  0.0252 | 30  15  0.8761  0.7667  0.0880 | 30  12  0.8762  0.9667  0.9740 | 30  10  0.7889  0.7931  0.8346 | 30  9  0.7811  0.8667  0.8670 | 29.9166  14  0.8394  0.8522 |
| DERWENT  (18)  DERW.New  Newlands Beck | N  No. alleles  H_E_  H_O_  HWE | 32  19  0.9227  1.0000  0.7730 | 32  9  0.8185  0.8125  0.6312 | 32  13  0.8190  0.8125  0.5164 | 32  20  0.9160  1.0000  0.4728 | 32  20  0.9003  0.9062  0.2261 | 32  4  0.6032  0.5937  0.9708 | 32  13  0.8620  0.8437  0.4515 | 32  14  0.8511  0.8750  0.6232 | 31  15  0.8611  0.5484  0.0000 | 31  13  0.8759  0.9355  0.9147 | 31  9  0.8335  0.8710  0.0287 | 32  11  0.7002  0.6875  0.0944 | 31.7500  13.3333  0.8303  0.8238 |
| KENT  (19)  KENT.SpA  Sprint (Ref.  NWKEN/03) | N  No. alleles  H_E_  H_O_  HWE | 41  20  0.9261  0.9756  0.1518 | 41  12  0.8312  0.9268  0.4169 | 41  14  0.8632  0.9024  0.9308 | 41  21  0.9169  1.0000  0.7475 | 41  22  0.9142  1.0000  0.1207 | 41  4  0.6704  0.6097  0.3297 | 41  15  0.8811  0.9024  0.2101 | 41  17  0.8840  0.9268  0.0223 | 41  17  0.8939  0.8049  0.0012 | 41  12  0.8748  0.9756  0.7434 | 41  9  0.8338  0.8293  0.3120 | 41  12  0.7810  0.8293  0.7020 | 41  14.5833  0.8558  0.8902 |
| KENT  (19)  KENT.Sto  Stockdate Beck | N  No. alleles  H_E_  H_O_  HWE | 20  17  0.8031  0.9500  0.2675 | 20  9  0.7972  0.6500  0.0035 | 20  13  0.8722  0.7500  0.3937 | 20  17  0.8838  0.9500  0.0274 | 20  16  0.8936  0.9500  0.0723 | 20  4  0.6726  0.7500  0.9494 | 20  12  0.8483  0.8000  0.0934 | 20  16  0.8470  0.7000  0.0252 | 20  16  0.8520  0.7000  0.0000 | 20  12  0.8284  0.9000  0.3334 | 20  9  0.8317  1.0000  0.8124 | 20  12  0.8466  0.8500  0.2380 | 20  12.7500  0.8361  0.8291 |
| LUNE  (20)  LUNE.Birk  Birk Beck (D/S Bretherale) | N  No. alleles  H_E_  H_O_  HWE | 29  20  0.9153  0.9310  0.4730 | 29  9  0.8210  0.7241  0.0070 | 29  16  0.8912  0.9655  0.8272 | 29  20  0.9112  0.8621  0.1559 | 29  23  0.8515  0.9310  0.6054 | 29  4  0.6471  0.6206  0.3405 | 29  14  0.8411  0.8275  0.1124 | 29  15  0.8808  0.8965  0.1391 | 29  16  0.8022  0.8276  0.5252 | 29  11  0.8217  0.8276  0.8632 | 29  8  0.8171  0.7931  0.2885 | 29  12  0.6994  0.6897  0.7798 | 29  14  0.8249  0.8247 |
| LUNE  (20)  LUNE.Cha  Chapel Beck (Howgill, Luffman Farm) | N  No. alleles  H_E_  H_O_  HWE | 28  19  0.9055  0.9286  0.8357 | 28  9  0.8016  0.7857  0.2379 | 28  13  0.8834  0.8929  0.5694 | 28  17  0.9031  0.9643  0.3245 | 28  21  0.9147  0.9642  0.6596 | 28  4  0.6656  0.7500  0.9907 | 28  13  0.8643  0.9285  0.2611 | 28  12  0.8508  0.9642  0.7430 | 28  16  0.8742  0.8929  0.0356 | 28  11  0.8244  0.8929  0.5286 | 28  8  0.7985  0.7857  0.3014 | 28  10  0.7647  0.7857  0.5424 | 28  12.7500  0.8375  0.8779 |
| LUNE  (20)  LUNE.Ger  River Greta (Ingleton A65 Bridge) | N  No. alleles  H_E_  H_O_  HWE | 30  21  0.9256  1.0000  0.2994 | 30  9  0.8320  0.7333  0.0335 | 30  17  0.8955  0.9333  0.1798 | 30  17  0.9036  0.9333  0.4337 | 30  19  0.8927  0.9000  0.4094 | 30  4  0.6123  0.5666  0.6084 | 30  13  0.8052  0.8666  0.8702 | 30  13  0.8686  0.8666  0.6769 | 30  14  0.8644  0.9333  0.6186 | 30  11  0.8515  0.9000  0.4229 | 30  9  0.8257  0.9333  0.3007 | 30  11  0.8310  0.7333  0.2061 | 30  13.1666  0.8423  0.8583 |
| RIBBLE  (21)  RIB.Bro  River Hodder (D/S Broadsden Farm Hill) | N  No. alleles  H_E_  H_O_  HWE | 28  21  0.9093  0.9643  0.9318 | 28  12  0.8342  0.8571  0.5896 | 28  16  0.8982  1.0000  0.0303 | 28  20  0.9136  0.9643  0.4345 | 28  16  0.8828  0.9642  0.6397 | 28  4  0.5936  0.5714  0.1443 | 29  15  0.8256  0.7241  0.0333 | 27  15  0.8481  0.5925  0.0004 | 29  19  0.8682  0.8621  0.7972 | 29  12  0.8354  0.8276  0.2219 | 28  5  0.7387  0.6071  0.1051 | 27  12  0.8582  0.8148  0.2964 | 28.0833  13.9166  0.8338  0.8124 |
| RIBBLE  (21)  RIB.Cra  River Ribble (Cragg Hill Farm) | N  No. alleles  H_E_  H_O_  HWE | 29  17  0.8987  0.9655  0.6606 | 29  8  0.7889  0.7931  0.4036 | 29  13  0.8539  0.8966  0.5276 | 29  18  0.9032  0.8966  0.0005 | 29  15  0.8819  0.9310  0.8417 | 29  4  0.5851  0.6896  0.8066 | 29  12  0.7763  0.8275  0.8272 | 29  15  0.8118  0.6206  0.0001 | 29  17  0.8849  1.0000  0.7861 | 29  12  0.8654  0.9655  0.5771 | 29  6  0.7557  0.7931  0.1030 | 29  8  0.7883  0.6897  0.0480 | 29  12.0833  0.8161  0.8390 |
| RIBBLE  (21)  RIB.Ham  River Hodder (D/S Hammerton Hall) | N  No. alleles  H_E_  H_O_  HWE | 31  21  0.9030  0.9355  0.8649 | 31  9  0.8068  0.7419  0.3477 | 31  13  0.8446  0.8710  0.8618 | 31  18  0.9046  1.0000  0.7448 | 31  14  0.8607  0.8709  0.7840 | 31  4  0.6003  0.6774  0.6165 | 31  11  0.8537  0.9354  0.2395 | 31  11  0.8203  0.5806  0.0011 | 31  15  0.8769  0.9355  0.7946 | 31  10  0.8552  0.9355  0.0237 | 31  9  0.7530  0.8065  0.4535 | 31  12  0.7625  0.7419  0.4282 | 31  12.2500  0.8201  0.8360 |
| DEE  (22)  DEE.Abb  Abbey Brook | N  No. alleles  H_E_  H_O_  HWE | 24  19  0.8981  1.0000  0.0477 | 24  10  0.7915  0.8333  0.3315 | 24  14  0.8822  0.9167  0.1140 | 24  20  0.8680  0.8333  0.0490 | 24  19  0.9047  1.0000  0.1695 | 24  4  0.6175  0.6666  0.6447 | 23  12  0.8601  0.8260  0.2295 | 20  17  0.8805  0.9500  0.6521 | 20  11  0.7996  0.7500  0.5031 | 18  11  0.8695  1.0000  0.4610 | 18  6  0.7148  0.7778  0.9772 | 20  7  0.7700  0.8000  0.4933 | 21.9166  12.5000  0.8213  0.8628 |
| DEE  (22)  DEE.Cer  Ceiriog | N  No. alleles  H_E_  H_O_  HWE | 39  21  0.9085  0.8718  0.7266 | 39  12  0.8124  0.8718  0.8854 | 39  13  0.8544  0.9231  0.7613 | 39  17  0.9025  0.9487  0.6004 | 39  18  0.8998  0.9230  0.3266 | 39  4  0.5519  0.5128  0.8311 | 37  17  0.8401  0.7027  0.0169 | 37  16  0.8846  0.8108  0.0517 | 36  17  0.8749  0.8889  0.1172 | 33  13  0.8626  0.8788  0.2083 | 33  8  0.7561  0.6061  0.0114 | 36  10  0.7912  0.8333  0.5160 | 37.1666  13.8333  0.8282  0.8143 |
| TEIFI  (23)  TEIFI.Cle  Clettwr (Dolbantan) | N  No. alleles  H_E_  H_O_  HWE | 27  19  0.9025  0.9259  0.8330 | 27  12  0.8153  0.8519  0.1565 | 27  14  0.8543  0.9259  0.9895 | 27  16  0.8708  0.8519  0.7223 | 27  22  0.9209  0.8888  0.4090 | 27  4  0.6507  0.6296  0.9887 | 27  19  0.9177  1.0000  0.8139 | 27  13  0.7677  0.8148  0.9488 | 27  15  0.8873  0.9259  0.2404 | 27  12  0.8747  0.8519  0.3553 | 27  9  0.7922  0.8148  0.2424 | 27  12  0.8086  0.7407  0.0839 | 27  13.9166  0.8385  0.8518 |
| TEIFI  (23)  TEIFI.Egn  Nant Egnant | N  No. alleles  H_E_  H_O_  HWE | 24  17  0.8863  0.8333  0.3734 | 24  11  0.7907  0.8333  0.8831 | 24  10  0.8280  0.7500  0.3315 | 24  14  0.8530  0.7500  0.2792 | 24  14  0.8821  0.8750  0.5653 | 24  4  0.5976  0.7083  0.4882 | 24  15  0.8738  0.9166  0.5598 | 24  13  0.8413  0.7500  0.1488 | 24  11  0.8313  0.7500  0.0441 | 24  11  0.8413  0.7500  0.0905 | 24  8  0.7966  0.9167  0.2144 | 24  13  0.7973  0.8750  0.6101 | 24  11.7500  0.8182  0.8090 |
| TEIFI  (23)  TEIFI.Lam  Teifi (Lampeter) | N  No. alleles  H_E_  H_O_  HWE | 27  21  0.9157  0.9259  0.5601 | 27  14  0.8550  0.9630  0.2204 | 27  13  0.8768  1.0000  0.9431 | 27  17  0.8840  0.8889  0.1226 | 27  20  0.9083  0.8148  0.0082 | 27  4  0.6158  0.7037  0.2287 | 27  15  0.8641  0.7777  0.0927 | 27  17  0.8767  0.8518  0.1040 | 27  15  0.8569  0.8148  0.1222 | 27  11  0.8212  0.8148  0.8052 | 27  10  0.8192  0.8148  0.2031 | 27  12  0.8205  0.7778  0.1040 | 27  14.0833  0.8428  0.8456 |
| USK  (24)  USK.Bra  River Bran | N  No. alleles  H_E_  H_O_  HWE | 29  17  0.8964  0.9655  0.9960 | 29  15  0.8774  0.9655  0.9026 | 29  12  0.8808  0.9310  0.8009 | 29  19  0.9089  0.8621  0.0308 | 29  19  0.9210  0.9655  0.3512 | 29  4  0.62578  0.44827  0.0146 | 29  11  0.8153  0.8620  0.7555 | 29  17  0.8705  0.9310  0.1062 | 29  15  0.8613  0.8276  0.7906 | 29  10  0.8401  0.8966  0.9062 | 29  8  0.8011  0.9310  0.5255 | 29  13  0.8142  0.7241  0.1042 | 29  13.3333  0.8427  0.8591 |
| USK  (24)  USK.Gir  River Ysgir | N  No. alleles  H_E_  H_O_  HWE | 30  18  0.8970  0.8000  0.0471 | 30  11  0.8536  0.9000  0.2188 | 30  15  0.8751  0.9333  0.9112 | 30  18  0.9106  0.9667  0.0735 | 30  22  0.9094  0.8666  0.0399 | 30  4  0.5897  0.5000  0.3079 | 30  14  0.8691  0.9333  0.9611 | 30  18  0.8455  0.8333  0.4912 | 30  13  0.8236  0.9667  0.8685 | 30  10  0.8466  0.9000  0.4794 | 30  9  0.8015  0.7667  0.3230 | 30  12  0.7876  0.8667  0.2099 | 30  13.6666  0.8341  0.8527 |
| USK  (24)  USK.Grw  River Grwyrn | N  No. alleles  H_E_  H_O_  HWE | 29  18  0.9102  1.0000  0.0554 | 29  10  0.8205  0.8621  0.2241 | 29  12  0.8527  0.8966  0.5139 | 29  19  0.9021  0.9655  0.7870 | 29  19  0.9078  0.9310  0.4420 | 29  4  0.4610  0.4827  0.2375 | 29  11  0.8096  0.8620  0.7807 | 28  19  0.8618  0.8928  0.9143 | 29  11  0.8487  0.8621  0.4394 | 29  13  0.8860  0.8966  0.6180 | 29  9  0.8022  0.8621  0.4894 | 29  10  0.8067  0.7931  0.0917 | 28.9166  12.9166  0.8224  0.8588 |
| WYE  (25)  WYE.Edw  River Edw | N  No. alleles  H_E_  H_O_  HWE | 30  20  0.9063  0.9667  0.9830 | 30  13  0.8353  0.8333  0.7380 | 30  15  0.8627  0.8667  0.2454 | 30  14  0.8998  0.9000  0.5451 | 30  18  0.9121  0.9333  0.6460 | 30  4  0.5967  0.4667  0.0740 | 29  12  0.8601  0.8620  0.1680 | 29  17  0.8641  0.7586  0.0474 | 29  15  0.8722  0.8966  0.1824 | 29  11  0.8659  0.8966  0.5325 | 29  9  0.7935  0.7931  0.4535 | 29  11  0.8176  0.7931  0.7907 | 29.5  13.25  0.9833  0.8405 |
| WYE  (25)  WYE.Lly (Wye)  River Llynfi | N  No. alleles  H_E_  H_O_  HWE | 30  18  0.9143  0.9333  0.5839 | 30  14  0.8300  0.9667  0.2530 | 30  12  0.8445  0.9333  0.4235 | 30  19  0.9106  0.9667  0.9705 | 30  19  0.9180  1.0000  0.7598 | 30  4  0.5404  0.6333  0.8769 | 30  12  0.8653  0.8333  0.6052 | 30  16  0.7826  0.7333  0.1090 | 30  11  0.8509  0.8667  0.1758 | 30  11  0.8455  0.8667  0.5509 | 30  7  0.8107  0.9000  0.9512 | 30  9  0.7993  0.8333  0.4724 | 30  12.6666  0.8260  0.8722 |
| WYE  (25)  WYE.Gar.  Garth Dulas | N  No. alleles  H_E_  H_O_  HWE | 27  18  0.9111  1.0000  0.7113 | 27  11  0.8331  0.9259  0.4514 | 27  12  0.8464  1.0000  0.6906 | 27  19  0.9144  1.0000  0.0844 | 27  21  0.9065  0.9629  0.1232 | 27  3  0.574  0.5185  0.1334 | 27  12  0.8516  0.8148  0.0915 | 27  15  0.8597  1.0000  0.4141 | 27  12  0.8383  0.6667  0.0026 | 27  9  0.8417  0.7407  0.0178 | 27  10  0.8200  0.9259  0.1657 | 27  11  0.8536  0.8889  0.3654 | 27  12.75  0.8376  0.8704 |
| SEVERN  (26)  SEV.CinA  Cinderford Brook | N  No. alleles  H_E_  H_O_  HWE | 22  15  0.8780  0.8636  0.0017 | 22  9  0.7647  0.8636  0.7340 | 22  9  0.8446  0.9545  0.7338 | 22  13  0.8752  1.0000  0.00149 | 22  13  0.8535  0.9545  0.7319 | 22  4  0.6768  0.6818  0.0398 | 22  12  0.8347  0.9090  0.2451 | 22  12  0.7743  0.6818  0.0081 | 22  11  0.8040  0.7273  0.1421 | 22  10  0.8190  0.9545  0.7117 | 22  9  0.7923  0.8182  0.2992 | 22  11  0.8398  1.0000  0.4547 | 22  10.6666  0.8130  0.8674 |
| TAW  (27)  TAW.Bra  Bray (Leworthy) | N  No. alleles  H_E_  H_O_  HWE | 25  18  0.8905  0.9200  0.4199 | 25  10  0.7821  0.8000  0.1433 | 25  11  0.8559  0.8400  0.8175 | 25  19  0.9005  0.9600  0.0223 | 25  19  0.9066  0.9600  0.6344 | 25  4  0.7007  0.8000  0.9666 | 25  11  0.8567  0.9600  0.2764 | 25  12  0.8566  0.8400  0.2335 | 25  9  0.8206  0.8800  0.2360 | 25  11  0.8452  0.9600  0.4766 | 25  8  0.7675  0.7200  0.0728 | 25  11  0.8367  0.9200  0.5370 | 25  11.9166  0.8349  0.8800 |
| TAW  (27)  TAW.Twi  Twitchen Stream (U/S West Molland) | N  No. alleles  H_E_  H_O_  HWE | 32  18.0000  0.8819  0.9375  0.8053 | 32  9.0000  0.7906  0.7500  0.0569 | 32  12.0000  0.8649  0.8750  0.2397 | 31  20.0000  0.9071  0.9677  0.6021 | 32  25  0.9301  0.9375  0.1151 | 32  4  0.6326  0.7500  0.8706 | 32  15  0.8843  0.9375  0.3435 | 32  14  0.8696  0.8750  0.5713 | 32  17.0000  0.8933  0.9063  0.0186 | 31  10.0000  0.8673  0.9355  0.8708 | 32  9.0000  0.7546  0.7500  0.4296 | 32  10.0000  0.7741  0.8438  0.9444 | 31.8333  13.5833  0.8375  0.8721 |
| TORRIDGE  (28)  TOR.Eoak  East Oakement (D/S A30 Road Bridge) | N  No. alleles  H_E_  H_O_  HWE | 21  16  0.8871  1.0000  0.4398 | 21  10  0.8005  0.7619  0.2937 | 21  13  0.8752  0.9524  0.4002 | 21  16  0.8828  0.9524  0.4569 | 21  19  0.9097  0.9523  0.8605 | 21  4  0.5834  0.6190  0.5305 | 21  11  0.8578  0.9047  0.7236 | 21  11  0.8460  0.9047  0.0622 | 20  11  0.8210  0.7500  0.0602 | 20  10  0.8270  0.8500  0.1787 | 20  7  0.7761  0.9500  0.6796 | 20  9  0.7073  0.9000  0.7329 | 20.6666  11.4166  0.8145  0.8748 |
| TORRIDGE  (28)  TOR.Woak  West Oakement (Oakhampton Castle) | N  No. alleles  H_E_  H_O_  HWE | 29  12  0.8292  0.9310  0.4333 | 29  7  0.7999  0.8966  0.0444 | 29  10  0.8413  0.9655  0.0189 | 29  14  0.8768  0.9655  0.2436 | 29  12  0.8447  0.9655  0.3692 | 29  4  0.6581  0.7931  0.0860 | 29  10  0.8400  0.8965  0.0099 | 29  15  0.8366  0.8620  0.0120 | 29  8  0.8240  0.8966  0.3886 | 29  9  0.8011  0.8966  0.3924 | 29  7  0.6701  0.6552  0.4822 | 29  7  0.7069  0.8276  0.8201 | 29  9.5833  0.7940  0.8793 |
| CAMAL  (29)  CAM.Del  (Key Bridge, De Lank) | N  No. alleles  H_E_  H_O_  HWE | 30  22  0.9196  1.0000  0.2212 | 30  11  0.7794  0.7333  0.1022 | 30  13  0.8745  0.9094  0.5508 | 30  19  0.8906  0.8666  0.6783 | 30  18  0.9121  0.9000  0.0367 | 30  4  0.6279  0.6666  0.7944 | 30  12  0.8820  0.9666  0.4173 | 30  15  0.8514  0.9333  0.0576 | 26  16  0.8906  0.8666  0.6783 | 30  13  0.8379  0.7333  0.0023 | 30  7  0.7655  0.7692  0.3203 | 30  13  0.7606  0.7666  0.1218 | 29.6666  13.5833  0.8342  0.8418 |
| CAMAL  (29)  CAM.Gam  Gam | N  No. alleles  H_E_  H_O_  HWE | 30  17  0.8799  0.9000  0.4604 | 30  10  0.7907  0.9000  0.1546 | 30  12  0.8529  0.7666  0.017 | 30  16  0.8820  0.8000  0.0253 | 30  18  0.8826  1.0000  0.0185 | 30  4  0.6129  0.6000  0.6956 | 30  12  0.7735  0.8333  0.2323 | 30  13  0.8547  0.9333  0.2387 | 30  12  0.8475  0.6666  0.0729 | 30  11  0.8562  0.9000  0.401 | 30  7  0.7369  0.7142  0.096 | 28  10  0.7300  0.7666  0.5235 | 29.8333  11.8333  0.8083  0.8150 |
| CAMAL  (29)  CAM.Ken  (Kenning-stock) | N  No. alleles  H_E_  H_O_  HWE | 30  22  0.9164  0.9667  0.5208 | 30  12  0.8332  0.9000  0.2898 | 30  12  0.8611  0.9333  0.7252 | 30  16  0.8751  0.9333  0.9520 | 30  19  0.9180  0.9666  0.9146 | 30  4  0.5883  0.7333  0.7158 | 30  12  0.8363  0.8333  0.3485 | 30  12  0.7740  0.8000  0.2552 | 30  15  0.8589  0.8000  0.0944 | 30  11  0.8449  0.8667  0.0630 | 29  7  0.7562  0.8275  0.0438 | 30  10  0.6698  0.6333  0.1895 | 29.9166  12.6666  0.8110  0.8495 |
| FOWEY  (30)  FOW.Mar  (Margate Ford) | N  No. alleles  H_E_  H_O_  HWE | 20  13  0.8711  0.9500  0.1997 | 20  13  0.8628  1.0000  0.3290 | 20  12  0.8533  1.0000  0.8775 | 20  12  0.8521  0.9500  0.6920 | 20  16  0.9007  0.9500  0.0122 | 20  4  0.6632  0.8000  0.3705 | 20  10  0.8365  0.9000  0.9468 | 20  17  0.8959  0.9500  0.0193 | 20  11  0.8472  0.8500  0.0402 | 20  11  0.8615  0.9000  0.0946 | 20  8  0.7273  0.8000  0.8979 | 20  9  0.7260  0.7500  0.2009 | 20  11.3333  0.8248  0.9000 |
| FOWEY  (30)  FOW.Tre  Treverbyn | N  No. alleles  H_E_  H_O_  HWE | 36  21  0.8996  0.8889  0.0386 | 36  13  0.8103  0.8333  0.8500 | 36  12  0.8651  0.8889  0.4500 | 36  17  0.9034  0.9167  0.1705 | 36  20  0.8853  0.8888  0.2483 | 36  4  0.5349  0.5555  1.0000 | 36  11  0.8800  0.8333  0.0265 | 36  13  0.7968  0.8333  0.6950 | 36  16  0.8460  0.7778  0.1092 | 36  9  0.8268  0.8333  0.3747 | 36  8  0.7758  0.7500  0.1546 | 36  11  0.7488  0.8056  0.2469 | 36  12.9166  0.8144  0.8171 |
| TAMAR  (31)  TAM.Gat  Lyd  (Gatherly) | N  No. alleles  H_E_  H_O_  HWE | 33  25  0.9174  0.9697  0.7017 | 33  10  0.7901  0.9394  0.8572 | 33  14  0.8363  0.7576  0.4265 | 33  19  0.9049  0.9394  0.6625 | 33  20  0.9058  0.9696  0.7911 | 33  4  0.6413  0.8787  0.0826 | 33  14  0.8786  0.9696  0.2542 | 33  18  0.8831  0.9393  0.3850 | 33  18  0.8893  0.8485  0.3934 | 33  11  0.8345  0.8182  0.1132 | 32  9  0.8317  0.7500  0.4738 | 33  11  0.7682  0.7273  0.0876 | 32.9166  14.4166  0.8401  0.8756 |
| TAMAR  (31)  TAM.Ott  Inny  (Bealsmill) | N  No. alleles  H_E_  H_O_  HWE | 33  21  0.8902  0.8788  0.3564 | 33  12  0.8145  0.7879  0.2427 | 33  10  0.8554  0.7576  0.3532 | 33  17  0.8755  0.8182  0.0120 | 33  21  0.9177  0.8787  0.1146 | 33  4  0.6198  0.6060  0.7139 | 32  14  0.8881  1.0000  0.0622 | 33  15  0.8572  0.9090  0.3022 | 33  15  0.8737  0.8485  0.0835 | 33  10  0.8399  0.7879  00171 | 33  8  0.7900  0.8485  0.1748 | 33  12  0.7513  0.7576  0.6876 | 32.9166  13.2500  0.8311  0.8232 |
| TAMAR  (31)  TAM.Tre  Ottery  (Trengune) | N  No. alleles  H_E_  H_O_  HWE | 30  16  0.8891  1.0000  0.8305 | 30  12  0.7860  0.9000  0.4876 | 30  13  0.8649  0.8667  0.1378 | 30  17  0.8853  0.9000  0.3526 | 30  17  0.8911  0.9000  0.8636 | 30  4  0.6682  0.7000  0.6902 | 30  13  0.8149  0.9000  0.0090 | 30  15  0.7289  0.7333  0.5152 | 30  16  0.8696  0.6000  0.0001 | 30  12  0.8767  0.9333  0.3829 | 30  8  0.7783  0.7000  0.2211 | 30  12  0.6430  0.6667  0.3636 | 30  12.9166  0.8080  0.8166 |
| DART  (32)  DAR.Pos & Post 04&05 East Dart (Postbridge) | N  No. alleles  H_E_  H_O_  HWE | 84  25  0.9061  0.9524  0.0107 | 84  13  0.8773  0.8929  0.2608 | 84  12  0.8315  0.7976  0.1341 | 84  21  0.9169  0.9643  0.1814 | 84  21  0.9198  0.9523  0.1506 | 84  4  0.6507  0.6904  0.4827 | 84  20  0.8990  0.9404  0.2928 | 84  17  0.9064  0.8333  0.0013 | 83  18  0.8885  0.8554  0.0252 | 84  11  0.8486  0.8452  0.1637 | 84  9  0.8007  0.7262  0.0738 | 84  14  0.7905  0.8571  0.3237 | 83.9166  15.4166  0.8564  0.8589 |
| EXE  (33)  EXE.Dan  Danes Brook (Slade Bridge) | N  No. alleles  H_E_  H_O_  HWE | 43  17  0.8746  0.8837  .0171 | 43  10  0.8033  0.7907  0.6620 | 43  13  0.8118  0.8372  0.0369 | 43  15  0.8966  0.8837  0.0212 | 43  19  0.8897  1.0000  0.3147 | 43  4  0.5856  0.6511  0.0040 | 42  11  0.8659  0.8571  0.1283 | 41  18  0.8794  0.9756  0.3102 | 39  14  0.8428  0.8205  0.4188 | 42  10  0.8451  0.8095  0.3458 | 40  8  0.8237  0.9000  0.4127 | 42  9  0.7912  0.7619  0.7333 | 42  12.3333  0.8258  0.8476 |
| EXE  (33)  EXE.Sher  Sherdon Water (Fernyball) | N  No. alleles  H_E_  H_O_  HWE | 31  19  0.8869  0.9677  0.5842 | 31  10  0.7942  0.8065  0.7342 | 31  13  0.8345  0.8710  0.8757 | 31  15  0.8904  0.9355  0.2304 | 31  19  0.9060  0.9354  0.6552 | 31  4  0.5998  0.6129  0.8734 | 31  12  0.8708  0.9354  0.3640 | 31  12  0.8229  0.7741  0.1044 | 31  13  0.8531  0.7742  0.0000 | 31  11  0.7987  0.6774  0.0249 | 31  8  0.7923  0.8710  0.2855 | 31  9  0.8225  0.8710  0.7042 | 31  12.0833  0.8226  0.8360 |
| EXE  (33)  EXE.Sim  Barle (Simonsbath) | N  No. alleles  H_E_  H_O_  HWE | 68  19  0.8937  0.8235  0.0037 | 68  12  0.7655  0.7059  0.1555 | 68  16  0.8101  0.8088  0.1579 | 68  17  0.9003  0.8971  0.0079 | 68  24  0.9199  0.9852  0.0504 | 68  4  0.5362  0.5294  0.2310 | 68  18  0.8971  0.8235  0.3115 | 68  17  0.8943  0.8823  0.0217 | 68  16  0.8931  0.8088  0.0365 | 68  11  0.8300  0.7500  0.0276 | 66  9  0.7781  0.8636  0.2022 | 68  13  0.8485  0.8235  0.1839 | 67.8333  14.6667  0.8305  0.8084 |
| AVON (Hampshire)  (34)  AVON.Brd  Upper Avon (Avon Bridge) | N  No. alleles  H_E_  H_O_  HWE | 23  13  0.8503  0.9565  0.5873 | 23  5  0.7563  0.9130  0.8374 | 23  6  0.7082  0.7826  0.6642 | 23  9  0.8122  0.8260  0.1627 | 23  11  0.8503  1.0000  0.1772 | 23  4  0.5535  0.5652  0.7462 | 23  5  0.7481  0.9130  0.2992 | 23  7  0.7770  0.8260  0.0647 | 23  10  0.8078  0.9130  0.4398 | 23  5  0.6739  0.7391  0.0221 | 23  4  0.6259  0.6521  0.0957 | 23  5  0.5933  0.6086  0.3640 | 23  7  0.7297  0.8079 |
| AVON (Hampshire)  (34)  AVON.Bug  (Bugmoor Hatches) | N  No. alleles  H_E_  H_O_  HWE | 20  14  0.8613  0.7500  0.0788 | 20  5  0.7283  0.7000  0.1084 | 20  7  0.7651  0.7500  0.8986 | 20  10  0.7759  0.8000  0.6254 | 20  12  0.8711  1.0000  0.9978 | 20  4  0.4621  0.4500  0.1595 | 20  8  0.6724  0.6000  0.1134 | 20  5  0.7699  0.8000  0.5917 | 20  14  0.8889  1.0000  0.9729 | 20  6  0.7570  0.8500  0.0099 | 20  6  0.7069  0.7000  0.5529 | 20  5  0.5726  0.5000  0.0849 | 20  8  0.7360  0.7416 |
| ITCHEN  (35)  ITC.Bis  (Bishopstoke Barge) | N  No. alleles  H_E_  H_O_  HWE | 53  16  0.8520  0.9057  0.6301 | 53  6  0.7479  0.7925  0.0994 | 53  5  0.7460  0.7170  0.7419 | 53  13  0.8686  0.9057  0.2231 | 53  13  0.8509  0.8679  0.1938 | 53  4  0.5759  0.4716  0.0196 | 53  10  0.7988  0.7169  0.1531 | 53  15  0.7987  0.8113  0.1475 | 53  12  0.8572  0.7736  0.3193 | 53  8  0.7006  0.7358  0.2710 | 53  8  0.6487  0.5849  0.1794 | 53  10  0.6557  0.6604  0.0624 | 53  10  0.7584  0.7452 |
| TEST  (36)  TEST.31-50  (Moorcourt Carrier) | N  No. alleles  H_E_  H_O_  HWE | 49  18  0.8914  0.9592  0.0304 | 49  5  0.7585  0.7143  0.2871 | 49  7  0.7402  0.8163  0.9284 | 49  17  0.9113  0.9184  0.1662 | 49  16  0.8893  0.9795  0.4407 | 49  5  0.4935  0.4897  0.8777 | 49  11  0.7742  0.8571  0.3231 | 48  8  0.7034  0.6875  0.0063 | 50  13  0.8701  0.9200  0.0295 | 46  6  0.6989  0.8043  0.2408 | 47  8  0.6339  0.6809  0.2066 | 49  6  0.4798  0.4490  0.0081 | 48.5833  10  0.7370  0.7730 |
| **France** |  |  |  |  |  |  |  |  |  |  |  |  |  |  |
| SEE  (37)  SEE  Across the Catchment | N  No. alleles  H_E_  H_O_  HWE | 49  21  0.9140  0.8980  0.6555 | 49  17  0.8754  0.8163  0.2915 | 49  12  0.7934  0.8367  0.9993 | 50  16  0.8960  0.8600  0.4132 | 50  23  0.9279  0.9200  0.6517 | 50  4  0.6539  0.6600  0.9604 | 49  13  0.8411  0.7551  0.0036 | 49  15  0.7958  0.6122  0.0011 | 45  12  0.8055  0.8667  0.0138 | 49  12  0.8575  0.9388  0.3011 | 49  10  0.7610  0.8163  0.2517 | 50  9  0.7241  0.6800  0.2281 | 49  13.6666  0.8204  0.8050 |
| SELUNE  (38)  SELUNE  Across the Catchment | N  No. alleles  H_E_  H_O_  HWE | 50  22  0.9074  0.9400  0.0710 | 50  15  0.8811  0.9600  0.1492 | 50  14  0.8109  0.8800  0.4262 | 50  19  0.9111  0.9600  0.3264 | 50  25  0.9322  0.9400  0.2230 | 50  4  0.6439  0.6000  0.3635 | 50  16  0.8456  0.8600  0.7584 | 50  13  0.8352  0.7200  0.0000 | 49  10  0.7406  0.6531  0.0279 | 50  13  0.8599  0.8200  0.2269 | 50  8  0.7555  0.7800  0.1226 | 50  14  0.6957  0.7400  0.3652 | 49.9167  14.4166  0.8182  0.8210 |
| LEUGER  (39)  LEUGER  Across the Catchment | N  No. alleles  H_E_  H_O_  HWE | 48  21  0.9066  0.9792  0.9677 | 48  12  0.8596  0.7917  0.0636 | 48  16  0.8620  0.8542  0.0616 | 48  19  0.8917  0.9167  0.4677 | 48  24  0.9318  0.9375  0.1603 | 48  4  0.5043  0.5833  0.4267 | 49  16  0.8795  0.8571  0.2093 | 49  11  0.7483  0.5918  0.0004 | 49  15  0.8342  0.6327  0.0110 | 49  11  0.8422  0.8776  0.9321 | 49  8  0.7906  0.7959  0.4536 | 49  13  0.7677  0.7755  0.1291 | 48.5000  14.1666  0.8182  0.7994 |
| ELORN  (40)  ELORN  Across the Catchment | N  No. alleles  H_E_  H_O_  HWE | 49  19  0.9187  0.8980  0.0680 | 49  15  0.8352  0.8163  0.4505 | 49  14  0.8663  0.8980  0.0125 | 49  22  0.9183  0.8980  0.0403 | 49  24  0.9168  0.9183  0.0163 | 49  4  0.4986  0.4081  0.0478 | 49  12  0.7632  0.7551  0.6305 | 49  11  0.7389  0.7346  0.2408 | 49  14  0.7659  0.6531  0.0043 | 49  9  0.7675  0.7755  0.2907 | 49  9  0.7912  0.7755  0.4317 | 49  9  0.5154  0.5510  0.5563 | 49  13.5000  0.7746  0.7568 |
| BLAVET  (41)  BLAVET  Across the Catchment | N  No. alleles  H_E_  H_O_  HWE | 49  21  0.8931  0.8775  0.2560 | 49  14  0.8356  0.9591  0.6924 | 49  16  0.8634  0.8775  0.0970 | 49  19  0.8911  0.9795  0.8028 | 49  25  0.9187  1.0000  0.7055 | 49  4  0.6047  0.5714  0.6246 | 49  15  0.8556  0.8979  0.5100 | 49  14  0.7373  0.7142  0.6867 | 49  13  0.8429  0.8163  0.2873 | 49  10  0.7864  0.7959  0.0891 | 49  9  0.7632  0.7142  0.0316 | 49  10  0.7091  0.7346  0.0524 | 49  14.1666  0.8084  0.8282 |
| ELLE  (42)  ELLE  Across the Catchment | N  No. alleles  H_E_  H_O_  HWE | 50  22  0.9168  0.9800  0.4457 | 50  11  0.8574  0.8400  0.1158 | 50  14  0.8536  0.7800  0.0353 | 50  22  0.9205  1.0000  0.9309 | 50  23  0.9185  0.9400  0.3747 | 50  4  0.5115  0.4400  0.0710 | 50  17  0.8869  0.7600  0.0046 | 50  11  0.7479  0.6200  0.0018 | 50  14  0.8386  0.9000  0.5928 | 50  12  0.8078  0.8000  0.6269 | 50  10  0.7682  0.7200  0.0620 | 50  11  0.7466  0.5600  0.0000 | 50  14.2500  0.8145  0.7783 |
| AULNE  (43)  AULNE  Across the Catchment | N  No. alleles  H_E_  H_O_  HWE | 38  20  0.8814  0.9473  0.4071 | 38  12  0.8008  0.7368  0.5661 | 38  14  0.8541  0.8421  0.1752 | 38  24  0.9229  0.9210  0.2446 | 38  26  0.9229  0.9210  0.2197 | 38  4  0.5864  0.6315  0.4689 | 39  15  0.8706  0.7179  0.0019 | 39  13  0.7944  0.6410  0.018 | 39  15  0.8207  0.8205  0.1461 | 39  10  0.8527  0.7179  0.0206 | 39  9  0.7931  0.7179  0.0561 | 39  10  0.6006  0.6666  0.5585 | 38.5000  14.3333  0.8084  0.7735 |
| SCORFF  (44)  SCORFF  Across the Catchment | N  No. alleles  H_E_  H_O_  HWE | 47  21  0.9168  0.9149  0.3658 | 47  12  0.8067  0.7872  0.2567 | 47  15  0.8539  0.8723  0.6086 | 47  19  0.9110  0.8298  0.1363 | 47  25  0.9105  0.9148  0.6793 | 47  4  0.5933  0.6382  0.3264 | 48  15  0.8511  0.8541  0.3156 | 48  12  0.7238  0.6875  0.0531 | 48  14  0.8341  0.7292  0.0049 | 48  11  0.8277  0.7917  0.3102 | 48  10  0.7591  0.7083  0.4068 | 48  8  0.6977  0.5208  0.0169 | 47.5000  13.8333  0.8071  0.7707 |
| **Spain** |  |  |  |  |  |  |  |  |  |  |  |  |  |  |
| CARES  (47)  CARES02 | N  No. alleles  H_E_  H_O_  HWE | 72  24  0.9281  0.8611  0.0100 | 74  15  0.8282  0.8513  0.7716 | 75  14  0.7964  0.7333  0.2015 | 73  26  0.9312  0.8630  0.0732 | 75  26  0.9254  0.9466  0.0495 | 75  5  0.6142  0.6000  0.5325 | 75  16  0.8599  0.92  0.9381 | 74  12  0.8062  0.7162  0.1258 | 73  16  0.8661  0.8493  0.5244 | 72  10  0.7254  0.8194  0.2936 | 67  7  0.5894  0.6119  0.7141 | 74  10  0.8071  0.8648  0.1320 | 73.2500  15.0833  0.8065  0.8031 |
| CARES  (47)  CAR.Casano | N  No. alleles  H_E_  H_O_  HWE | 24  12  0.8597  0.9583  0.0038 | 25  7  0.6760  0.6000  0.1494 | 25  8  0.7691  0.8400  0.2334 | 25  16  0.8605  0.9600  0.1744 | 25  11  0.8489  0.8800  0.1160 | 25  4  0.5786  0.7200  0.3985 | 25  8  0.7539  0.9090  0.1469 | 20  8  0.7296  0.8000  0.0441 | 24  8  0.7997  0.7500  0.1193 | 23  6  0.7271  0.6521  0.0052 | 23  4  0.5210  0.5652  1.0000 | 24  6  0.7914  0.8333  0.3830 | 23.7500  8.1666  0.7430  0.7890 |
| SELLA  (48)  SELLA02 | N  No. alleles  H_E_  H_O_  HWE | 48  20  0.9280  0.8958  0.1371 | 46  13  0.8078  0.8043  0.3787 | 49  13  0.8104  0.8367  0.6079 | 47  22  0.9166  0.8936  0.3256 | 48  23  0.9187  0.9375  0.6649 | 49  5  0.6475  0.5918  0.7581 | 50  18  0.8612  0.7800  0.0285 | 50  16  0.7803  0.7800  0.1171 | 50  16  0.8580  0.8800  0.0757 | 50  7  0.7165  0.7200  0.2404 | 50  8  0.6353  0.6000  0.0015 | 50  12  0.8027  0.7600  0.2713 | 48.9166  14.4166  0.8069  0.7899 |
| SELLA  (48)  SELLA.Pig | N  No. alleles  H_E_  H_O_  HWE | 34  17  0.8939  0.8529  0.0588 | 35  9  0.7701  0.6571  0.0025 | 34  13  0.7789  0.8529  0.1505 | 28  18  0.8613  0.9286  0.8715 | 31  15  0.8888  0.7419  0.0132 | 28  4  0.3716  0.4642  0.7718 | 35  14  0.8272  0.8571  0.0918 | 35  12  0.8204  0.7428  0.1186 | 34  12  0.7520  0.7941  0.0126 | 34  7  0.6378  0.6176  0.6233 | 34  8  0.5731  0.5294  0.0012 | 34  13  0.8104  0.7353  0.2006 | 33  11.8333  0.7488  0.7311 |
| NARCEA  (49)  NARCEA02 | N  No. alleles  H_E_  H_O_  HWE | 40  22  0.8993  0.9500  0.7319 | 42  14  0.8081  0.7857  0.3295 | 42  12  0.7071  0.7381  0.6818 | 42  18  0.8764  0.8333  0.5049 | 45  17  0.9001  0.8666  0.0327 | 44  4  0.5558  0.5681  0.7569 | 50  15  0.8175  0.7000  0.1495 | 50  11  0.8071  0.6600  0.0171 | 48  14  0.8309  0.8333  0.4779 | 39  8  0.6731  0.6923  0.5169 | 47  7  0.5204  0.5319  0.1382 | 49  11  0.8306  0.8163  0.1577 | 44.8333  12.7500  0.7688  0.7479 |
| EO  (50)  EO | N  No. alleles  H_E_  H_O_  HWE | 46  17  0.8965  0.9348  0.3191 | 46  10  0.7345  0.7391  0.8525 | 46  12  0.8427  0.8667  0.3528 | 46  15  0.9095  0.8696  0.5513 | 46  18  0.8960  0.9565  0.4208 | 45  5  0.5715  0.6000  0.4633 | 46  18  0.8923  0.8695  0.0381 | 46  17  0.8662  0.8043  0.4903 | 46  12  0.7611  0.7609  0.0679 | 46  10  0.7546  0.7391  0.4797 | 45  8  0.6657  0.5778  0.0000 | 45  10  0.7995  0.8000  0.0056 | 45.6666  12.6666  0.7991  0.7931 |
| ULLA  (51)  ULLA | N  No. alleles  H_E_  H_O_  HWE | 46  16  0.8716  0.8913  0.9575 | 46  10  0.6445  0.6522  0.6411 | 46  11  0.5750  0.5870  0.5707 | 46  14  0.8672  0.8478  0.3274 | 46  18  0.9041  0.93478  0.4580 | 46  5  0.4901  0.5434  0.0993 | 46  8  0.5617  0.6304  0.1209 | 46  12  0.7603  0.7608  0.0505 | 46  11  0.7546  0.8043  0.7585 | 46  7  0.7144  0.7391  0.4288 | 46  7  0.6096  0.6304  0.0383 | 46  8  0.6815  0.6957  0.2388 | 46  10.5833  0.7029  0.7264 |
| **Ireland** |  |  |  |  |  |  |  |  |  |  |  |  |  |  |
| MOY  (54)  MOY.TriL  Trimoge (Kilkelly) | N  No. alleles  H_E_  H_O_  HWE | 42  18  0.9063  0.9762  0.9148 | 42  13  0.8587  0.8810  0.8383 | 42  12  0.7967  0.8571  0.9181 | 42  24  0.9293  0.9524  0.5064 | 42  20  0.9140  0.9285  0.1676 | 42  4  0.5374  0.5476  0.0613 | 41  16  0.8677  0.8780  0.0465 | 42  15  0.8185  0.7857  0.0443 | 40  18  0.8983  0.7500  0.0035 | 40  12  0.8588  0.9500  0.6031 | 40  9  0.8222  0.9000  0.6470 | 42  11  0.8130  0.7381  0.1214 | 41.4166  14.3333  0.8351  0.8453 |
| LAUNE  (58)  LAU.Cot  Cottoners | N  No. alleles  H_E_  H_O_  HWE | 47  21  0.9179  0.9574  0.2363 | 47  12  0.7923  0.7447  0.3300 | 47  15  0.8543  0.9362  0.1712 | 47  24  0.9228  0.9362  0.8317 | 47  27  0.9320  0.9361  0.6740 | 47  4  0.6732  0.6382  0.4551 | 47  14  0.8671  0.7872  0.1642 | 47  17  0.8529  0.8085  0.0993 | 47  15  0.8833  0.8723  0.5245 | 47  10  0.8452  0.7872  0.1550 | 47  9  0.7437  0.6809  0.0131 | 47  10  0.6948  0.6596  0.2668 | 47  14.8333  0.8319  0.8156 |
| CORK BLACKWATER  (61)  COR.Awn  Awnaskirtaun | N  No. alleles  H_E_  H_O_  HWE | 35  22  0.9236  0.9142  0.2243 | 35  13  0.7463  0.7428  0.9031 | 35  12  0.8578  0.8857  0.8860 | 35  21  0.9185  1.0000  0.8145 | 35  21  0.9152  0.8571  0.4325 | 35  5  0.6238  0.5714  0.7198 | 35  15  0.8645  0.8857  0.8855 | 35  14  0.7780  0.7714  0.1755 | 35  16  0.8882  0.6571  0.0000 | 35  11  0.8641  0.8857  0.8748 | 35  9  0.7899  0.8000  0.2264 | 35  11  0.7641  0.7142  0.1107 | 35  14.1666  0.8278  0.8071 |
| CORK BLACKWATER  (61)  COR.Cly  Clydagh | N  No. alleles  H_E_  H_O_  HWE | 35  21  0.9196  0.9142  0.3425 | 35  14  0.7816  0.7142  0.1014 | 35  13  0.8443  0.9142  0.9775 | 35  20  0.9216  0.9714  0.8873 | 35  20  0.9053  0.8857  0.8837 | 35  4  0.6274  0.6571  0.9827 | 35  15  0.8689  0.8857  0.3945 | 35  17  0.8811  0.8571  0.3302 | 35  17  0.8831  0.7428  0.0021 | 35  10  0.8408  0.9714  0.1626 | 35  10  0.7642  0.7428  0.3447 | 35  13  0.8419  0.8285  0.1758 | 35  14.5000  0.8400  0.8404 |
| CORK BLACKWATER  (61)  COR.Gle  Glen | N  No. alleles  H_E_  H_O_  HWE | 35  23  0.9284  1.0000  0.1758 | 35  17  0.8328  0.8571  0.4009 | 35  12  0.8729  0.9428  0.6534 | 35  21  0.9173  0.9428  0.2831 | 35  21  0.9208  0.9142  0.0901 | 35  4  0.6678  0.6571  0.2737 | 35  14  0.8791  0.8285  0.1462 | 35  15  0.8542  0.8285  0.2266 | 35  19  0.9089  0.9714  0.8544 | 35  11  0.8729  0.9428  0.6063 | 35  10  0.8185  0.8285  0.5288 | 35  10  0.7292  0.6857  0.5495 | 35  14.7500  0.8502  0.8666 |
| BARROW  (62)  BAR.Bal  Barrow (Ballyclare Br) | N  No. alleles  H_E_  H_O_  HWE | 40  21  0.9227  0.9750  0.7922 | 40  13  0.8468  0.9000  0.8119 | 40  20  0.9029  0.9500  0.8781 | 39  19  0.8893  0.8974  0.9770 | 40  20  0.9005  0.9500  0.4431 | 40  4  0.6968  0.6000  0.1036 | 40  18  0.8986  0.9250  0.3684 | 39  22  0.8844  0.8205  0.0006 | 40  18  0.9056  0.9250  0.6281 | 39  14  0.8822  0.7948  0.0067 | 39  9  0.7752  0.7948  0.6354 | 40  12  0.7776  0.7750  0.0659 | 39.8333  15.6666  0.8567  0.8616 |
| SUIR  (63)  SUIR.Clo  Clodiagh | N  No. alleles  H_E_  H_O_  HWE | 28  21  0.9124  1.0000  0.1401 | 28  11  0.8385  0.8929  0.2512 | 28  14  0.8760  0.9286  0.4743 | 28  19  0.9080  0.9643  0.9458 | 28  21  0.9184  0.9285  0.5839 | 28  5  0.7031  0.7857  0.5839 | 28  12  0.8600  0.9285  0.2437 | 28  18  0.8723  0.9285  0.7898 | 28  16  0.8771  0.6071  0.0000 | 28  11  0.8644  0.9286  0.7736 | 28  9  0.7621  0.6429  0.1492 | 28  11  0.7326  0.6786  0.1063 | 28  14  0.8437  0.8511 |
| SUIR  (63)  SUIR.MCb  Suir (Beakstown) | N  No. alleles  H_E_  H_O_  HWE | 20  15  0.8806  1.0000  0.9048 | 20  10  0.8103  0.7500  0.1164 | 20  11  0.8365  0.8500  0.8682 | 20  14  0.8591  0.9000  0.7338 | 20  17  0.8769  0.9500  0.2583 | 20  4  0.5774  0.5500  0.6290 | 20  12  0.8495  0.8500  0.2323 | 20  14  0.8364  0.8000  0.0552 | 20  13  0.8554  0.8000  0.0123 | 20  9  0.8295  0.9500  0.8755 | 20  7  0.7520  0.6500  0.0282 | 20  9  0.7796  0.9000  0.9378 | 20  11.2500  0.8119  0.8291 |
| BOYNE  (65)  BOY.Dee  Deel (Raharney) | N  No. alleles  H_E_  H_O_  HWE | 36  19  0.9060  0.9722  0.9801 | 36  15  0.8155  0.7222  0.1295 | 36  12  0.8666  0.9167  0.9294 | 36  17  0.8966  0.9167  0.0567 | 36  19  0.9026  0.9166  0.6351 | 36  4  0.6253  0.5833  0.3542 | 36  14  0.7503  0.7777  0.5101 | 36  12  0.8654  0.8333  0.8087 | 36  16  0.8789  0.8055  0.013 | 36  10  0.7844  0.6388  0.0307 | 36  7  0.7885  0.8055  0.3273 | 36  10  0.7716  0.6666  0.3201 | 36  12.9166  0.8210  0.7962 |
| BOYNE  (65)  BOY.Moy  Moynalty (Borora) | N  No. alleles  H_E_  H_O_  HWE | 35  19  0.9232  0.9142  0.0238 | 35  12  0.7851  0.6857  0.0083 | 35  12  0.8617  0.8571  0.2942 | 35  17  0.8523  0.9714  0.3483 | 35  19  0.9065  0.9428  0.3665 | 35  4  0.6797  0.7428  0.9629 | 35  9  0.7871  0.7428  0.2060 | 35  13  0.8732  0.7428  0.0076 | 35  16  0.8676  0.8000  0.0479 | 35  10  0.8530  0.9142  0.8355 | 35  7  0.7916  0.9142  0.4273 | 35  9  0.7154  0.6571  0.0560 | 35  12.2500  0.8247  0.8238 |
| BOYNE  (65)  BOY.Ska  Skane Lwr | N  No. alleles  H_E_  H_O_  HWE | 35  20  0.9216  1.0000  0.7439 | 35  13  0.8558  0.9428  0.5571 | 35  17  0.8911  0.8857  0.0790 | 35  20  0.9030  0.9142  0.0919 | 35  18  0.8768  0.9142  0.7046 | 35  4  0.6646  0.6571  0.3515 | 35  14  0.8129  0.7428  0.3508 | 35  15  0.8915  0.8857  0.6654 | 35  15  0.8858  0.6857  0.0004 | 35  10  0.8478  0.8000  0.1937 | 35  9  0.8106  0.8857  0.6412 | 35  15  0.7467  0.7142  0.2234 | 35  14.1666  0.8424  0.8357 |
| Total | N  No. alleles  H_E_  H_O_ | 3704  38  0.9525  0.9357 | 3708  29  0.8663  0.8220 | 3710  31  0.9025  0.8563 | 3701  40  0.9454  0.9270 | 3711  40  0.9539  0.9323 | 3707  8  0.6614  0.6342 | 3714  33  0.9131  0.8506 | 3692  32  0.8993  0.8085 | 3692  32  0.9141  0.8142 | 3673  26  0.8836  0.8402 | 3669  13  0.8238  0.7741 | 3706  18  0.8164  0.7590 |  |
