# Supplementary material for: Genetic stock identification of Atlantic salmon (Salmo salar) populations in the southern part of the European range
Source: BMC Genet. 2010 Apr 29;11:31. doi: 10.1186/1471-2156-11-31 (PMC2882343; doi:10.1186/1471-2156-11-31)
Supplement: Additional file 6 — STRUCTURE analysis of baseline samples. STRUCTURE analysis [53], demonstrating clustering of baseline samples into regional groupings. [file 1471-2156-11-31-S6.DOC]

**Additional File 6.** Structure analysis, demonstrating clustering of baseline samples into regional groupings. Each individual is represented by a thin horizontal line which is partitioned into K coloured segments, representing an individual’s estimated membership fractions in *K* clusters. A ‘hierarchical’ approach (Vaha *et. al.* 2007), with two rounds of analysis was employed in order to capture the major structure within the data (run-lengths varied from 50,000 to 200,000 burn-in and 100,000 to 400,000 total length, with four iterations). To judge the correct *K*, the *ΔK* method of Evanno *et al.* (2005) was applied. In the analysis of each cluster, plots of the absolute values of ln Pr(*X | K)* and *ΔK* were generated by Structure Harvester (Earl, 2009).


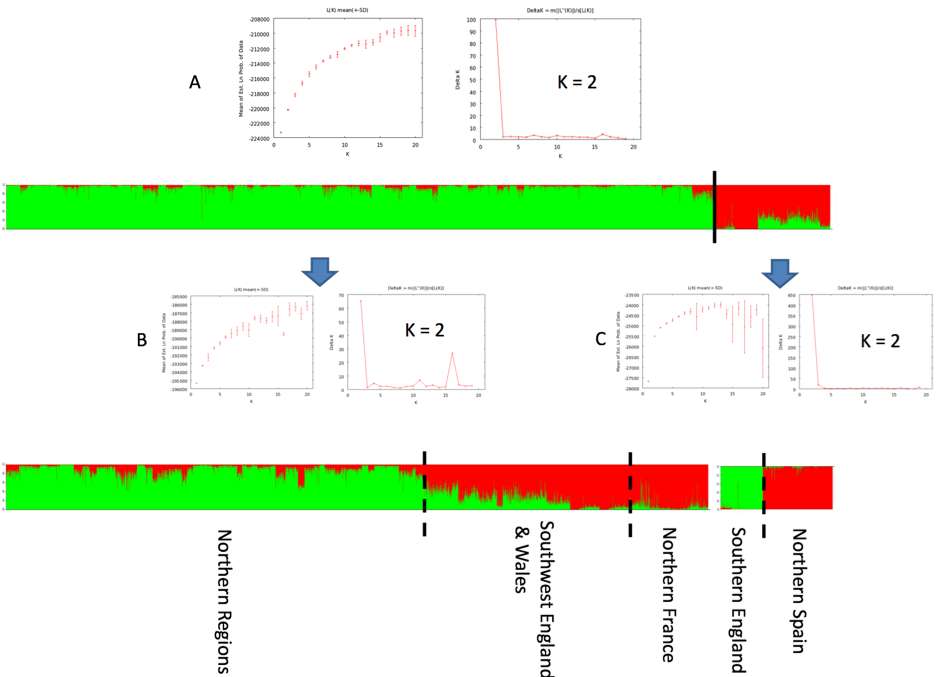


**Northern Spain** consists of samples sets from the rivers: Eo Ulla, Sella, Narcea and Cares.

**Southern England** consists of samples sets from the rivers: Avon, Itchen and Test.

**Northern France** consists of samples sets from the rivers: See, Selune, Leuger, Elorn, Aulne, Scorff and Blavet

**Southwest England and Wales** consisted of sample sets from the rivers: Teifi, Usk, Wye Severn, Taw, Torridge, Camel, Fowey, Tamar, Dart and Exe.

**Northern Regions** consists of the remaining samples originating from: Ireland, northern England and Scotland.

**References**

Earl, D. A. (2009) Structure Harvester v0.3, from website: <http://users.soe.ucsc.edu/~dearl/software/struct_harvest/>

Evanno, G., Regnaut, S. and Goudet, J. (2005). Detecting the number of clusters of individuals using the software STRUCTURE: a simulation study. *Molecular Ecology*, **14**, 2611 - 2620

Pritchard, J.K., Stephens, M. and Donnelly, P. (2000) Inference of population structure using multilocus genotype data. *Genetics*, **155**, 945–959.

Vähä, J.-P., Erkinaro, J. ,Niemelä, E. and Primmer, C.R. (2007) Life–history and habitat features influence the within–river genetic structure of Atlantic salmon. *Molecular Ecology,* **16**, 2638–2654.
